# The impact of perceived social support on college athletes’ academic burnout: The moderating role of cognitive reappraisal within a serial mediation model

**DOI:** 10.1371/journal.pone.0361222

**Published:** 2026-10-22

**Authors:** Guihua Xu, Xirui Yang, Qiyong Zhang

**Affiliations:** 1 College of Physical Education, Yangzhou University, Yangzhou, China; 2 School of Educational Science, Yangzhou University, Yangzhou, China; Universiti Pertahanan Nasional Malaysia, MALAYSIA

## Abstract

College athletes face substantial challenges from the combined demands of academic study and athletic training, placing them at elevated risk for academic burnout (AB). This study examined how perceived social support (PSS) is associated with AB by testing the mediating roles of mental toughness (MT) and athlete burnout (ATHB) and the moderating effect of cognitive reappraisal (CR). Using a cross-sectional design, 1,918 college athletes from over 20 Chinese universities completed standardized questionnaires assessing PSS, MT, ATHB, CR, and AB. Serial and moderated serial mediation were analyzed with the PROCESS macro (Models 6 and 89) while controlling for demographic and sport-related variables. PSS was directly and negatively associated with AB (β = −0.122, p < 0.001), with significant indirect associations separately through MT and ATHB and sequentially through MT and ATHB. CR showed path-specific moderation: at higher levels of CR, the negative PSS–AB association was stronger, but so was the positive ATHB–AB association, whereas the MT–AB association did not differ significantly across CR levels. Thus, CR should not be interpreted as having a uniformly buffering role. These findings may inform future multicomponent support approaches aimed at strengthening PSS and MT and identifying and addressing ATHB, while applying CR-related training cautiously and with attention to the specific pathway involved. The effectiveness and boundary conditions of such approaches require longitudinal or experimental testing.

## 1. Introduction

With the ongoing reforms in China’s higher education system, college students are experiencing growing academic demands, and academic burnout (AB) has drawn increasing attention as a prominent focus in educational psychology research [1,2]. AB is characterized by students’ experience of negative emotions and withdrawal tendencies that develop under sustained academic stress or declining engagement in learning [3,4], often manifested through emotional exhaustion, a sense of detachment, and reduced perceived academic competence [5,6]. A substantial body of research indicates that learning burnout is closely linked to poorer academic achievement [7], heightened intentions to withdraw from school, and increased risks of depressive symptoms, anxiety, and suicidal ideation [8–11]. However, college athletes constitute a distinctive group who must juggle academic responsibilities alongside competitive sport. In addition to the academic workload, social demands, and financial pressures faced by non-athlete students, they devote substantial time to training, competitions, team meetings, and injury management. The accumulation of these sport-related obligations on top of routine university stressors creates a dual academic–athletic load, thereby markedly increasing vulnerability to psychological strain and learning burnout [12]. Therefore, identifying psychosocial resources associated with lower AB among college athletes and clarifying how and under what conditions such resources are associated with AB carry important theoretical and practical implications.

Among various candidate protective factors, social support has been identified as a key resource that may buffer AB [1,13]. In particular, perceived social support (PSS) captures individuals’ appraisal of how accessible and how helpful support is, and it shows robust associations with psychological adjustment and stress-related responses [14,15]. Grounded in the stress-buffering model, social support is thought to attenuate the detrimental consequences of stress exposure. By easing negative affect and internal psychological strain, supportive resources can weaken the pathway through which stress undermines both mental and physical health [16]. In educational settings, accumulating evidence suggests that PSS is inversely associated with AB, such that students who perceive greater support tend to report fewer burnout symptoms [13,17]. Beyond academics, research in sport contexts further indicates that social support helps athletes cultivate stronger relational networks and bolsters their capacity to manage psychological and physical demands, competitive stressors, and everyday difficulties [18]. However, for college student-athletes, although social support has potential value in promoting psychological adaptation, existing research has paid relatively limited attention to the role of PSS in AB, and related conclusions still need further verification. In addition, examining only the overall association between PSS and AB is insufficient to clarify the potential psychological pathways linking them or the conditions under which the strength of this association may vary.

Conservation of Resources (COR) theory posits that individuals are motivated to acquire, retain, and protect valued resources. Repeated resource gains may generate a “gain spiral” through which existing resources facilitate the acquisition of additional resources and reduce vulnerability to subsequent resource loss and exhaustion [19]. Within this framework, PSS reflects individuals’ subjective perceptions of the availability of external social resources, whereas mental toughness, as manifested in the sport context, may be viewed as an internal psychological resource. Mental toughness (MT) refers to a natural or developed psychological advantage that enables athletes to cope effectively with the demands of competition, training, and the athletic lifestyle while maintaining determination, focus, confidence, and control under pressure [20]. Crust and Clough (2011) suggested that challenging yet supportive environments created by coaches and parents provide important developmental conditions for mental toughness in young athletes [21]. Consistent with this view, perceived support from family members and significant others was positively associated with MT among university students [22]. In addition, higher MT was associated with lower levels of learning burnout among junior middle school students [23]. Cheung and Li also found that students characterized by relatively low academic burnout reported higher MT than those with moderate or high burnout levels [24]. Importantly, PSS from parents and classmates was indirectly associated with better school well-being through MT [25]. Although school well-being and AB are conceptually distinct, both reflect students’ psychological adjustment within the school context. This finding therefore provides related evidence that external social resources may be associated with academic adjustment outcomes through internal psychological resources. Taken together, these findings support a theoretically plausible resource-based pathway of “perceived external resources → internal psychological resources → academic burnout”, in which MT may mediate the association between PSS and AB.

Interpreting AB solely through the lens of general psychological resources may understate the dominant stressor for college student-athletes: sustained, high-intensity depletion arising from prolonged training and competitive demands. Athlete Burnout (ATHB) describes a maladaptive psychological syndrome that develops under prolonged high-intensity training and sustained competitive pressure, typically characterized by emotional and physical exhaustion, a reduced sense of accomplishment, and sport devaluation [26]. Existing research has linked PSS to lower levels of ATHB and its related dimensions [27,28]. In addition, research on young athletes with dual academic and athletic identities has shown a strong positive correlation between school burnout and sport burnout [29], indicating that burnout experiences in the school and sport domains are not independent of each other. Our preliminary work on college athletes also found that ATHB was significantly associated with AB [30]. Together, these findings suggest that sport-domain burnout may be linked to AB, providing a rationale for considering ATHB as a potential mediator between PSS and AB. Further empirical support comes from a previous study showing that MT mediated the association between social support and ATHB [31]. Although few studies have directly tested the full “PSS → MT → ATHB → AB” serial pathway, existing evidence provides an empirical basis for integrating these related associations. Grounded in COR theory, the present study examines “PSS → MT → ATHB → AB” as a serial pathway to clarify the potential links among external social resources, internal psychological resources, athletic-domain depletion, and academic-domain depletion under the dual academic–athletic demands faced by college athletes.

Although this proposed resource-to-depletion pathway is theoretically plausible, the strength of its component associations may vary across individuals. The process model of emotion regulation suggests that the strategies people deploy when encountering stress can shape their emotional experiences and, consequently, their downstream behavioral responses [32]. In particular, cognitive reappraisal (CR) is an antecedent-focused strategy that involves reframing the meaning of a situation to regulate emotional responses, and it has been linked to more favorable patterns of psychological adjustment [33]. Previous studies have provided preliminary support for this boundary-condition role. For example, CR has been found to moderate the association between intrusive rumination and AB [34], as well as the associations between stress and occupational burnout and between depression and job burnout [35,36]. These findings suggest that CR may be associated with variations in the strength of associations among burnout-related variables. However, direct evidence on whether CR moderates the associations among PSS, MT, ATHB, and AB in college athletes remains limited. Given that CR primarily reflects the reinterpretation of stressful situations and emotional experiences, the present study conceptualized it as a proximal moderating resource in relation to AB and examined its moderating role in the associations between PSS and AB, MT and AB, and ATHB and AB.

In summary, grounded primarily in COR theory, this study intends to construct a moderated serial mediation model to systematically test the independent and serial mediating roles of MT and ATHB in the relationship between PSS and AB, and to examine the moderating effect of CR on key paths (see Fig 1). Within this framework, the stress-buffering model and the process model of emotion regulation were used as complementary perspectives to explain the external-resource nature of PSS and the boundary-condition role of CR, respectively. Although the present study used survey data that partially overlapped with those used in our previously published article, it addressed a distinct theoretical question. In the published article, ATHB was treated as the antecedent variable, and the study examined the burnout-spillover process through which ATHB was associated with AB via CR and sleep quality. In contrast, grounded in COR theory, the present study conceptualized PSS as the antecedent variable and examined its association with AB through MT and ATHB, as well as the moderating role of CR. The model was developed and specified after completion of the analyses reported in the earlier publication but before the analyses reported in the present study were conducted. Therefore, the incremental contribution of the present study lies in shifting the focus from “how ATHB is associated with AB” to “how PSS and MT are associated with AB at different levels of CR.” The two studies were not integrated into a single comprehensive model in order to avoid excessive model complexity and to maintain a clear theoretical focus on the distinct research questions and hypothesized pathways addressed in each study.

**Fig 1:**
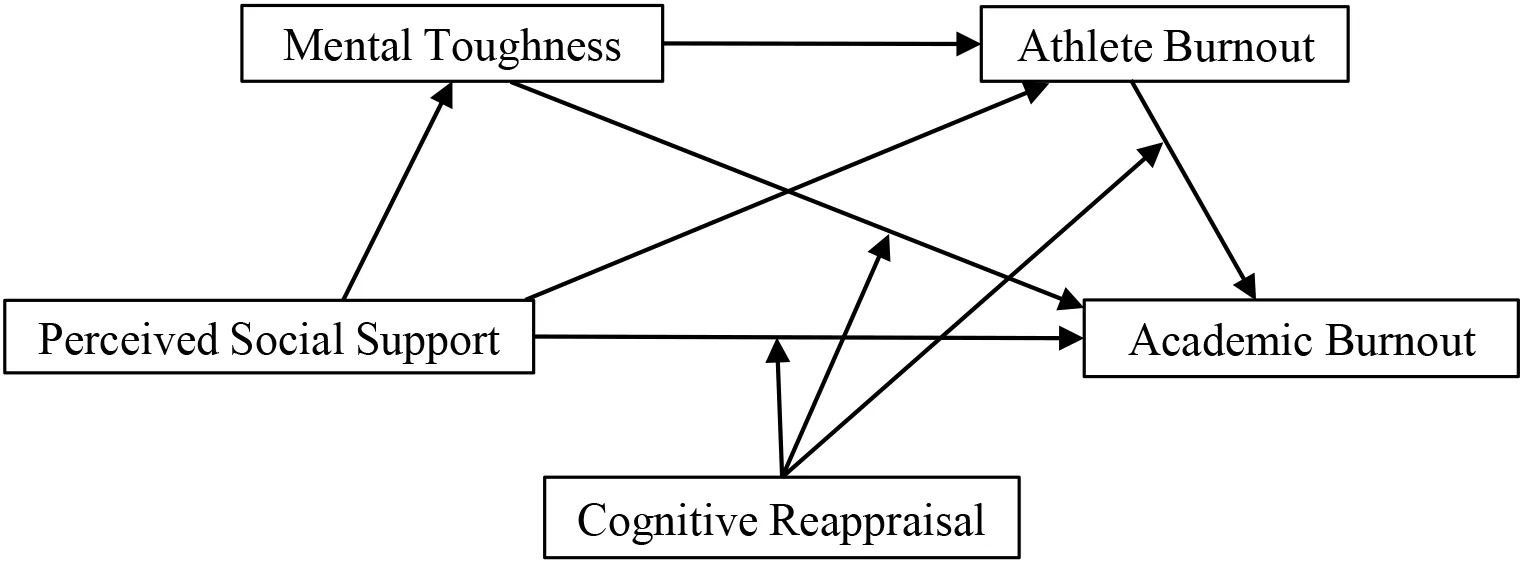
Conceptual model: A moderated serial mediation model.

Accordingly, the following hypotheses are proposed

H1 PSS is negatively correlated with AB among college student-athletes.
H2 MT mediates the relationship between PSS and AB.
H3 ATHB mediates the relationship between PSS and AB.
H4 MT and ATHB play a serial mediating role between PSS and AB.
H5 CR moderates the relationship between PSS and AB.
H6 CR moderates the relationship between MT and AB.
H7 CR moderates the relationship between ATHB and AB.
H8 : CR moderates the serial indirect effect of PSS on AB via MT and ATHB.

By constructing and testing the proposed moderated serial mediation model, this study aims to examine the potential indirect pathways and boundary conditions linking PSS with AB in the dual academic–athletic context and, on this basis, to propose preliminary, evidence-informed recommendations for psychological support and educational practice for college athletes.

## 2. Methods

This study was conducted from November to December 2024 using a cross-sectional design. Participants were recruited through convenience sampling from university sports teams across more than 20 universities in China. To ensure high response quality and participant engagement, we collaborated with faculty coordinators at each institution to distribute paper-based questionnaires directly to student-athletes. Investigators provided on-site guidance and addressed any questions participants had during the survey process. Prior to participation, all individuals were informed of the study’s purpose and procedures, as well as their right to withdraw at any time, and written informed consent was obtained from all participants. Ethical approval for this study was obtained from the Ethics Committee of the School of Educational Science, Yangzhou University (Approval No. JKYLL-2024–118). Of the 2,115 questionnaires distributed, 1,967 were returned (return rate: 93.00%). During data-quality screening, 49 questionnaires were excluded because of incomplete information, repetitive patterns (i.e., invariant responding within all multi-item scales included in the questionnaire), or logical inconsistencies (e.g., reporting no weekly sport-specific training but also reporting at least one training day per week or at least 5 hours of weekly sport-specific training). The final sample comprised 1,918 respondents, accounting for 97.51% of returned questionnaires and yielding an overall usable response rate of 90.69%. The mean age of the participants was 19.968 years (SD = 1.400). A summary of their demographic information is presented in Table 1. The dataset used in this study was collected from the same cohort of college athletes as in our previously published research [30]. The two studies share several variables including AB, ATHB and CR, but address different theoretical questions and test different analytical models.

**Table 1:** Demographic Characteristics of Participants (N = 1,918).

Variables	Category	n	%
Gender	Male	1262	65.80
	Female	656	34.20
Residence	Rural	500	26.07
	Township	218	11.37
	County-Level City	288	15.02
	Urban	912	47.55
Family Structure	Intact Family	1660	86.55
	Reconstituted Family	88	4.59
	Single-Parent Family	161	8.39
	Orphaned Family	9	0.47
Sport Item	Combat Sports (e.g., Wushu, boxing, taekwondo)	1089	56.78
	Ball Sports (e.g., soccer, basketball, volleyball)	494	25.76
	Track and Field (e.g., javelin throw, 800-meter run, long jump)	213	11.11
	Aquatic Sports (e.g., swimming, canoeing/kayaking)	56	2.92
	Other Sports (e.g., archery, cycling)	66	3.44
Sport Level	International-Level Elite Athlete	25	1.30
	National-Level Elite Athlete	78	4.07
	National Level-1 Athlete	555	28.94
	National Level-2 Athlete	1260	65.69

### 2.1 Instruments

#### 2.1.1 Perceived social support.

PSS among college athletes was measured through the Multidimensional Scale of Perceived Social Support (MSPSS), originally proposed by Zimet and colleagues. The MSPSS comprises 12 statements across three domains—family, friends, and significant others. Participants responded using a 7-point Likert format, where higher scores indicated stronger agreement [37]. The Chinese version of the MSPSS has been extensively validated in samples of Chinese college students and college athletes, demonstrating consistently strong reliability and validity [38,39]. In this study, the scale exhibited high internal reliability (Cronbach’s α = 0.950).

#### 2.1.2 Academic burnout.

The Learning Burnout Scale for College Students (LBUS), developed by Lian Rong and colleagues (2005), was employed to assess college athletes’ levels of AB [40]. The LBUS is composed of 20 questions categorized into three dimensions: emotional exhaustion (8 items, total score range 8–40), inappropriate behavior (6 items, 6–30), and diminished sense of accomplishment (6 items, 6–30). Participants responded using a five-point Likert scale ranging from 1 (completely inconsistent) to 5 (completely consistent), where higher scores represented greater levels of AB. In the present study, the total score was used because the proposed model focused on the overall level of academic burnout rather than its specific dimensions. The LBUS was originally developed in China and has been repeatedly validated in undergraduate samples, demonstrating strong reliability and validity. It has been widely adopted as a standard instrument for assessing learning burnout among Chinese college students [41]. In this study, the instrument showed satisfactory internal reliability (Cronbach’s α = 0.906).

#### 2.1.3 Emotion regulation questionnaire.

The Emotion Regulation Questionnaire (ERQ), developed by Gross and John in 2003, was used to assess participants’ habitual use of emotion-regulation strategies [33]. The ERQ comprises two dimensions: CR (6 items) and expressive suppression (4 items). Each statement is scored on a seven-point Likert scale ranging from 1 (strongly disagree) to 7 (strongly agree), with higher scores indicating more frequent use of the corresponding emotion-regulation strategy. The Chinese version of the ERQ has been validated by Zhang Chunqing and colleagues in samples of Chinese athletes and students, and has been widely utilized in research involving Chinese athletic populations [42,43]. Given the focus of this study on the moderating role of CR, only the CR subscale was included in the analyses. In this study, Cronbach’s α for the CR subscale (items 1, 3, 5, 7, 8, and 10) was 0.894.

#### 2.1.4 Athlete Burnout Questionnaire.

The Athlete Burnout Questionnaire (ABQ), originally developed by Raedeke and Smith (2001) and later localized by Zhang Liwei et al., was applied in this study to evaluate ATHB [44,45]. The ABQ comprises 15 items distributed across three dimensions: emotional/physical exhaustion, diminished sense of accomplishment, and sport devaluation, each containing five statements. Responses were rated on a five-point scale (1 = never, 5 = always), with reverse scoring applied to Items 1 and 14. Composite scores were obtained by summing all items, where higher totals reflect more severe ATHB. In this study, the total score was used because the hypothesized model examined the overall level of ATHB rather than its specific dimensions. The Chinese version of the ABQ has been widely employed in studies involving Chinese athlete populations [46]. Reliability analysis in this research showed acceptable internal consistency (Cronbach’s α = 0.869).

#### 2.1.5 Sports mental toughness questionnaire.

The SMTQ, originally developed by Sheard et al. and later adapted and validated in China by Wang Bin and colleagues, was used in this study to assess college athletes’ MT in coping with competitive stress and performance-related challenges [47,48]. The 12-item Chinese version comprises three dimensions—confidence, constancy, and control—with each item rated on a five-point scale ranging from 1 (strongly disagree) to 5 (strongly agree). Items 2, 4, 7, 8 and 9 were reverse-coded, and the item scores were summed to obtain a total MT score, with higher scores indicating stronger MT. The Chinese version of the SMTQ has been widely applied in Chinese athlete samples and has consistently demonstrated strong reliability and validity [49]. In this study, the scale showed acceptable reliability (Cronbach’s α = 0.851).

### 2.2 Statistical analysis

Data analyses were performed with SPSS version 26.0 and the PROCESS macro. The initial phase involved data cleaning and basic descriptive analysis, followed by the assessment of reliability and Pearson correlations among the study variables. The CFA model comparisons and ULMC analysis used the same 18 indicators: three established dimension scores from each of the MSPSS, SMTQ, ABQ, and LBUS, together with the six individual cognitive-reappraisal items. These indicators were based on the original scale structures; no item parcels or data-driven item groupings were used. Composite reliability, AVE, and HTMT were calculated using the same indicators. The serial mediation effects were examined with the PROCESS macro (Model 6), aiming to determine the pathways through which PSS is indirectly associated with AB via MT (M1) and ATHB (M2). The mediation effects were examined using a bias-corrected bootstrapping method with 5,000 resamples and 95% confidence intervals to derive estimates of total, direct, and indirect effects. Subsequently, the PROCESS macro (Model 89) was used to test whether CR moderated both the direct association between PSS and AB and the mediated pathways via MT and ATHB. Statistical significance was set at p < 0.05. Bootstrap effects were considered significant when the 95% confidence interval excluded zero.

Gender, age, family structure, residence, sport item, and sport level were included as covariates. Gender was binary coded, and multicategorical covariates were dummy coded using k-1 indicator variables, with male, intact family, urban residence, combat sports, and national level-2 athlete as reference categories. The focal variables were z-standardized prior to the mediation and moderation analyses.

The reported analyses were conducted to test the previously specified model and hypotheses (H1–H8) and were not conducted as post hoc exploratory analyses.

## 3. Results

### 3.1 Common method bias

Because the study relied on self-reported data, potential common method bias was examined following Podsakoff’s guidelines. Measures such as anonymous responses and mixed item wording were implemented to mitigate this issue. Additionally, Harman’s single-factor test using unrotated principal component analysis was conducted to evaluate common method variance [50]. The result yielded nine factors with eigenvalues greater than 1. The first factor explained 25.214% of the variance, far below the 40% reference value, indicating that common method variance was unlikely to substantially affect this study. To further assess common method bias, we conducted CFA-based alternative model comparisons and tested an unmeasured latent method construct (ULMC) model. As shown in S1 Table, the hypothesized five-factor CFA model showed better fit than the alternative models, with acceptable fit indices, χ^2^(125) = 1127.001, CFI = 0.952, TLI = 0.941, RMSEA = 0.065 and SRMR = 0.050. A freely loading ULMC model was subsequently estimated by specifying a latent method factor with freely estimated loadings on all 18 observed indicators. The variance of the method factor was fixed to 1, and its covariances with the five substantive factors were constrained to zero. The ULMC model showed improved fit, χ^2^(107) = 701.894, CFI = 0.971, TLI = 0.959, RMSEA = 0.054, and SRMR = 0.036, indicating the presence of additional shared covariance not accounted for by the baseline five-factor model. However, inspection of the parameter estimates revealed substantial variance overlap between the method factor and the substantive CR factor. Therefore, the improvement in model fit could not be attributed uniquely to method-related variance. Moreover, the latent correlations among PSS, MT, ATHB and AB retained their theoretically expected directions after the inclusion of the method factor, although their magnitudes were generally attenuated. Taken together, some shared method-related covariance cannot be completely ruled out; however, the available evidence does not indicate that a common method factor dominated the overall measurement structure, and its influence appeared to vary across the substantive constructs.

### 3.2 Correlation analysis and discriminant validity assessment

As shown in Table 2, PSS was negatively correlated with AB (r = −0.327, *p* < 0.01), supporting H1. PSS was also positively correlated with MT and CR and negatively correlated with ATHB (all *p* values < 0.01). MT was positively associated with CR and negatively associated with both ATHB and AB, whereas ATHB was negatively related to CR and positively related to AB (all *p* values < 0.01). Overall, higher PSS, MT, and CR were linked to lower levels of AB, whereas higher ATHB was associated with higher AB, providing preliminary evidence supporting the subsequent analyses of serial mediation and moderation effects.

**Table 2:** Descriptive Statistics and Pearson Correlations Among Variables (N = 1,918).

Variables	M ± SD	PSS	MT	ATHB	CR	AB
PSS	60.874 ± 14.824	1				
MT	40.287 ± 7.787	0.567^**^	1			
ATHB	36.966 ± 9.918	−0.356^**^	−0.451^**^	1		
CR	29.299 ± 6.989	0.658^**^	0.560^**^	−0.326^**^	1	
AB	53.199 ± 13.897	−0.327^**^	−0.369^**^	0.436^**^	−0.323^**^	1

Note: PSS = Perceived social support, MT = Mental toughness, ATHB = Athlete burnout, CR = Cognitive reappraisal, AB = Academic burnout, **p < 0.01.

As shown in S2 Table and S3 Table, composite reliability values ranged from 0.770 to 0.916, and AVE values ranged from 0.533 to 0.784. For each construct, the square root of AVE exceeded its correlations with the other constructs, and all HTMT ratios ranged from 0.379 to 0.727, below the commonly used threshold of 0.85. These results supported the convergent and discriminant validity of the five constructs.

Given the moderate correlations among several study variables, multicollinearity was further examined by entering all focal variables and covariates simultaneously into the same regression model. The VIF values ranged from 1.016 to 1.686, and tolerance values ranged from 0.593 to 0.984, all within acceptable ranges. These results indicated that multicollinearity was unlikely to substantially influence the regression estimates.

### 3.3 Test of the serial mediation effect

Using PROCESS Model 6 in SPSS, we tested the serial mediation effects. As shown in Fig 2, Table 3 and S4 Table, PSS showed a significant negative direct association with AB after accounting for the proposed mediators (MT and ATHB) and covariates (direct effect = −0.122, 95% CI [−0.170, −0.073]). In addition, three indirect paths were significant: via MT (effect = −0.087, 95% CI [−0.119, −0.056]), via ATHB (effect = −0.044, 95% CI [−0.064, −0.026]), and via the serial path MT → ATHB (effect = −0.069, 95% CI [−0.085, −0.056]). Together, these indirect effects accounted for the majority of the total effect of PSS on AB, providing support for H2–H4.

**Table 3:** Results of the Serial Mediation Analysis (N = 1,918).

Path	Effect	Proportion	SE	95% CI	
				Boot LLCL	Boot ULCL
PSS → AB	−0.122	37.89%	0.025	−0.170	−0.073
PSS → MT → AB	−0.087	27.02%	0.016	−0.119	−0.056
PSS → ATHB → AB	−0.044	13.66%	0.010	−0.064	−0.026
PSS → MT → ATHB → AB	−0.069	21.43%	0.007	−0.085	−0.056
Total indirect effect	−0.200	62.11%	0.018	−0.237	−0.166
Total effect	−0.322	100.00%	0.022	−0.364	−0.279

Note: PSS = Perceived social support, MT = Mental toughness, ATHB = Athlete burnout, AB = Academic burnout.

**Fig 2:**
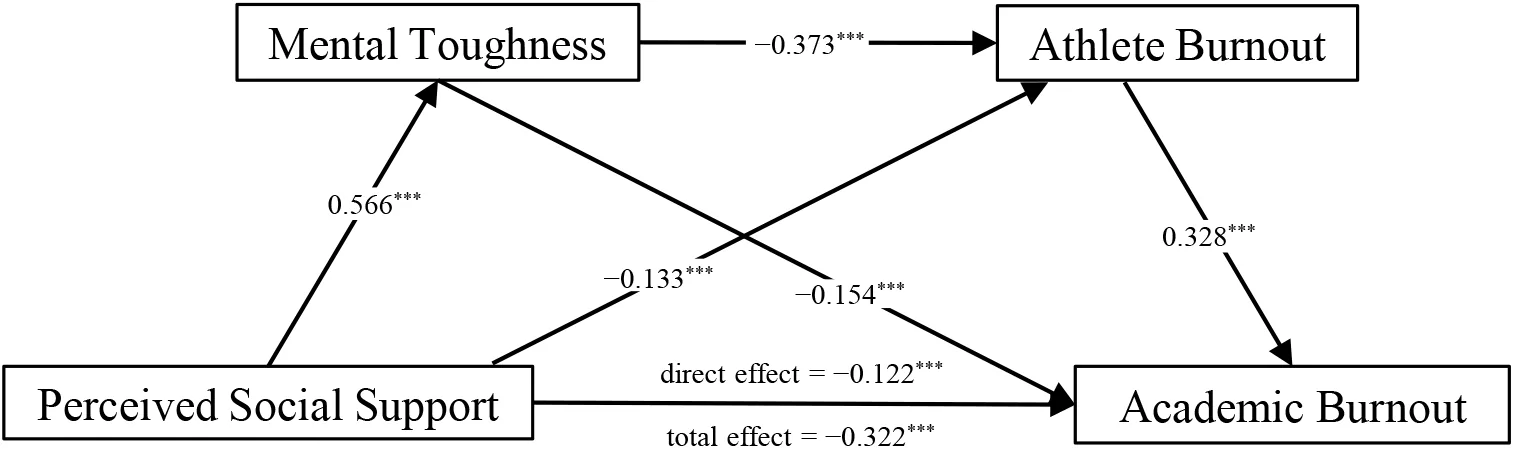
Serial mediation model (N = 1,918). Note: ***p < 0.001. Standardized path coefficients are reported.

### 3.4 Test of the moderated serial mediation model

To test whether CR moderated the hypothesized paths, we used PROCESS Model 89 (see Table 4 and Fig 3). All models included gender, age, family structure, residence, sport item, and sport level as covariates, with detailed coefficients reported in S5 Table. The AB equation explained 26.5% of the variance, R^2^ = 0.265, F(22, 1895) = 30.988, *p* < 0.001, corresponding to Cohen’s f^2^ = 0.361 and indicating a large overall explanatory effect. Predictor-specific local f^2^ values are reported in Table 4. Regarding the direct path from PSS to AB, CR was negatively associated with AB (β = −0.131, *p* < 0.001) and the PSS × CR interaction was significant (β = −0.050, *p* = 0.012). Simple slope tests (see Fig 4) showed that PSS was negatively associated with AB at high CR (M + 1 SD; effect = −0.158, *p* < 0.001) but not at low CR (M − 1 SD; effect = −0.057, *p* = 0.073), supporting H5. In contrast, the MT × CR interaction on AB was not significant (β = −0.001, *p* = 0.981), indicating that CR did not moderate the MT → AB path (H6 not supported). The ATHB × CR interaction on AB was significant (β = 0.080, *p* < 0.001), indicating that CR moderated the ATHB → AB path. Simple slope tests (see Fig 5) showed that ATHB was positively associated with AB at both low (M − 1 SD; β = 0.223, *p* < 0.001) and high levels of CR (M + 1 SD; β = 0.383, *p* < 0.001), with a stronger association under higher CR levels. Thus, H7 was supported. The conditional indirect effect of PSS on AB through ATHB was significant at both low CR (M − 1 SD; effect = −0.030, 95% CI [−0.044, −0.016]) and high CR (M + 1 SD; effect = −0.051, 95% CI [−0.076, −0.029]). The index of moderated mediation was −0.011 (95% CI [−0.020, −0.004]), indicating that the indirect association through ATHB varied across levels of CR. Finally, the conditional serial indirect effect was stronger at high CR (M + 1 SD; effect = −0.081, 95% CI [−0.101, −0.062]) than at low CR (M − 1 SD; effect = −0.047, 95% CI [−0.062, −0.033]). The index of moderated mediation was −0.017 (95% CI [−0.027, −0.007]), indicating that the strength of the PSS → MT → ATHB → AB serial indirect pathway varied as a function of CR, supporting H8.

**Table 4:** Results of the moderated serial mediation model analysis (N = 1,918).

Antecedents	Consequent									
	M1 (MT)			M2 (ATHB)			Y (AB)			
	β	SE	*p*	β	SE	*p*	β	SE	*p*	local f²
Constant	0.038	0.276	0.890	−0.784	0.295	0.008	0.098	0.291	0.735	
PSS	0.566	0.019	< 0.001	−0.133	0.025	< 0.001	−0.107	0.029	< 0.001	0.007
MT				−0.373	0.025	< 0.001	−0.113	0.027	< 0.001	0.009
ATHB							0.303	0.023	< 0.001	0.092
CR							−0.131	0.029	< 0.001	0.011
PSS × CR							−0.050	0.020	0.012	0.003
MT × CR							−0.001	0.021	0.981	0.000
ATHB × CR							0.080	0.021	< 0.001	0.007
Model statistics	R² = 0.335,			R² = 0.239,			R² = 0.265,			
	F(16, 1901) = 59.867, *p* < 0.001			F(17, 1900) = 35.037, *p* < 0.001			F(22, 1895) = 30.988, *p* < 0.001			

Note: PSS = Perceived social support, MT = Mental toughness, ATHB = Athlete burnout, CR = Cognitive reappraisal, AB = Academic burnout.

**Fig 3:**
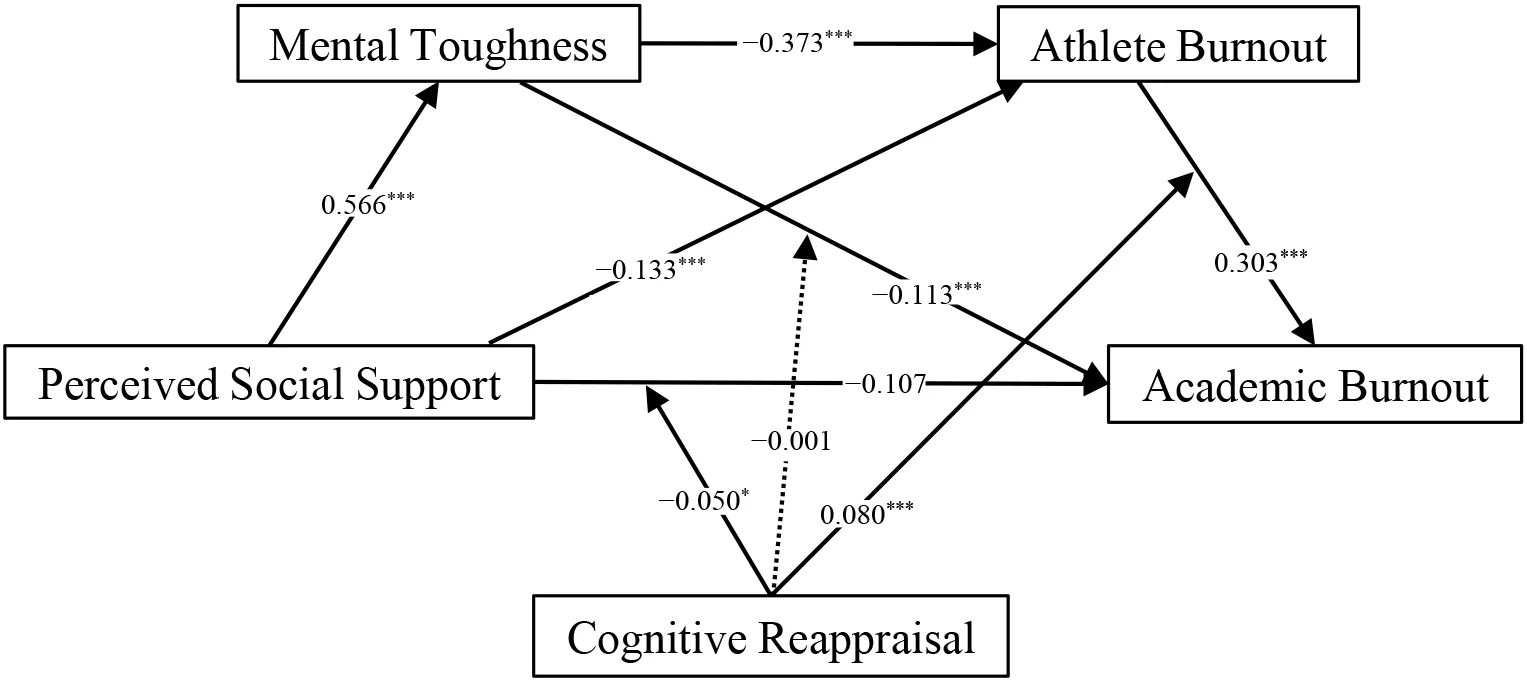
Moderated serial mediation model (N = 1,918). Note: *p < 0.05, ***p < 0.001. Values on arrows are standardized regression coefficients. The dashed arrow indicates a non-significant moderation effect.

**Fig 4:**
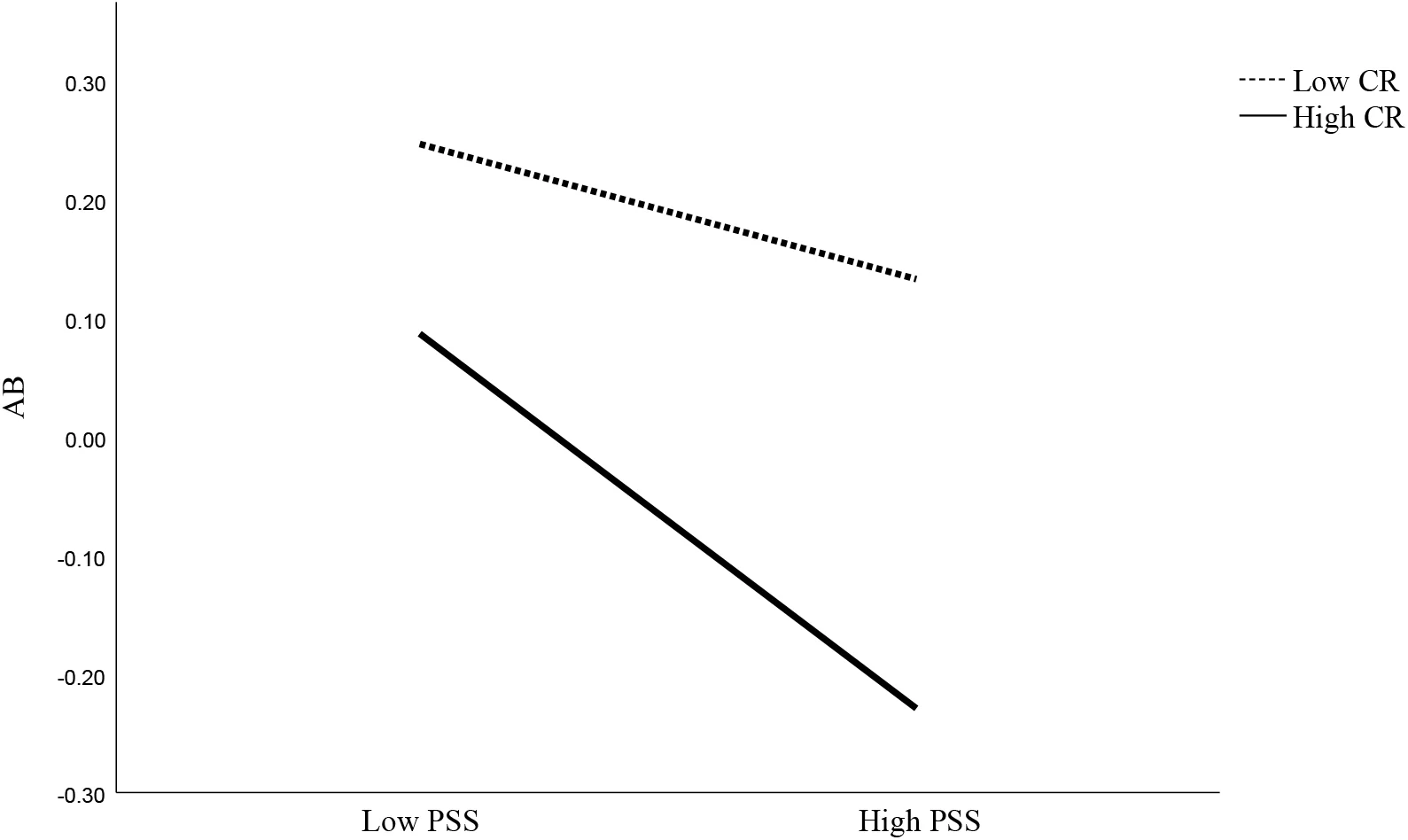
Moderating effect of CR on the relationship between PSS and AB: Simple slope plot. Note: PSS = Perceived social support, CR = Cognitive reappraisal, AB = Academic burnout.

**Fig 5:**
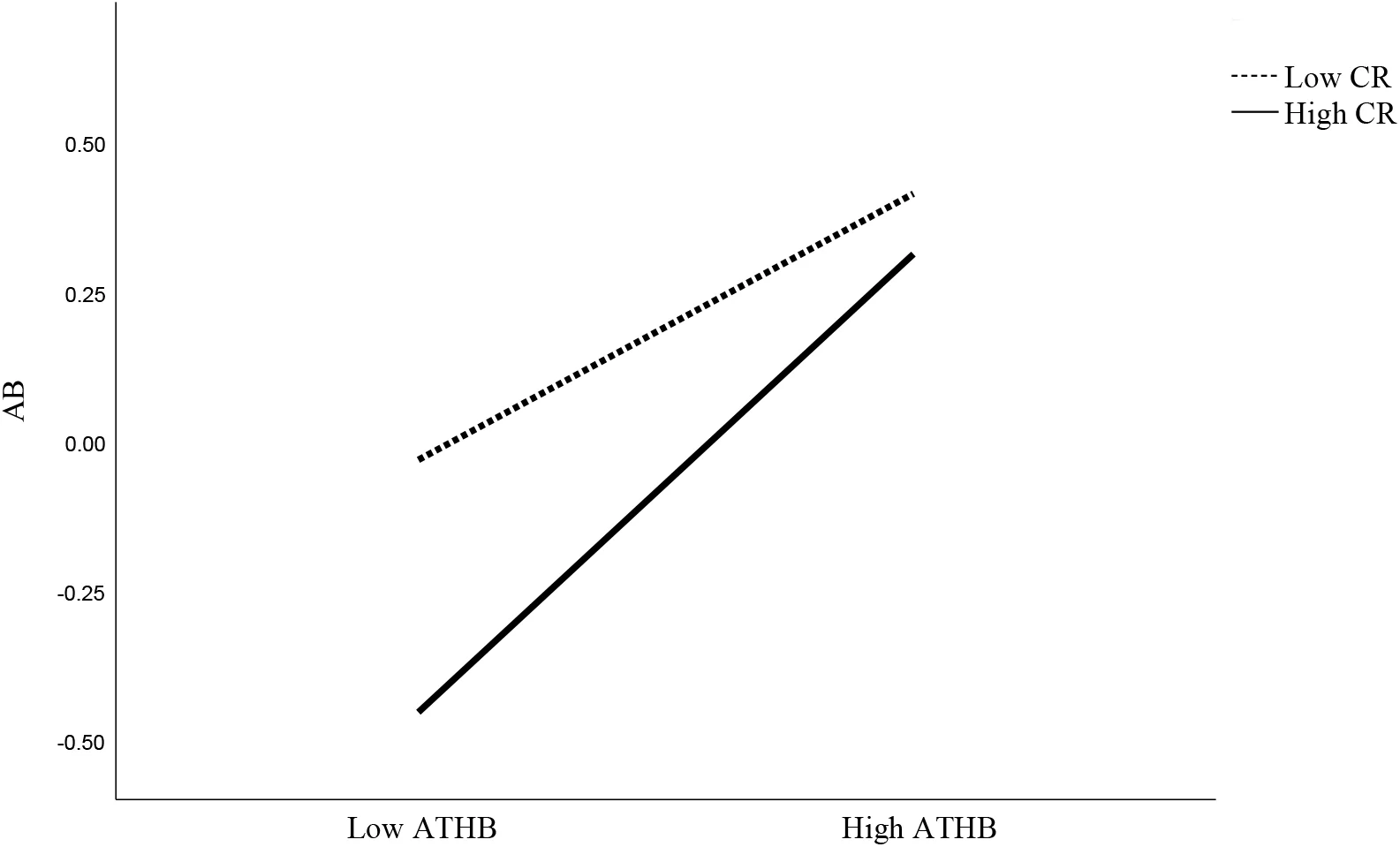
Moderating effect of CR on the relationship between ATHB and AB: Simple slope plot. Note: ATHB = Athlete burnout, CR = Cognitive reappraisal, AB = Academic burnout.

## 4. Discussion

This study examined how PSS relates to AB among college athletes facing dual academic and athletic demands. After controlling for relevant demographic and sport-related variables, PSS was negatively associated with AB, with this link mediated by MT and ATHB, while CR showed a path-specific moderating pattern. Although the present study was based on the same cohort as our previously published article and both studies involved ATHB, AB, and CR, the current study further situates ATHB within the pathway linking PSS and MT to AB, and examines CR as a moderating resource.

### 4.1 The relationship between perceived social support and academic burnout

The analysis revealed a significant negative association between PSS and AB. This finding supports H1 and adds to evidence of an inverse association between PSS and AB. Consistent with prior research, insufficient social support has been associated with greater chronic stress—a major antecedent of burnout—whereas higher PSS has been associated with lower levels of exhaustion and disengagement related to AB [1,51]. Based on COR theory, social support represents an important external social resource, whereas PSS reflects college athletes’ subjective appraisal of the availability and usability of this resource. For college athletes facing both academic and athletic demands, higher PSS may indicate greater perceived access to emotional, informational, and instrumental support from family members, teachers, peers, and significant others. Positive perceptions of these support resources may help athletes cope with academic stress and role demands, and may be associated with lower levels of AB. From a practical perspective, universities may enhance college athletes’ perception and use of social support by fostering supportive teacher–student relationships, strengthening family communication, and creating a positive peer climate. At the same time, teachers and academic support staff should attend to athletes’ need to coordinate training and academic roles, helping them translate external support into more specific resources for academic adjustment and better cope with dual academic and athletic demands.

### 4.2 The mediating role of mental toughness

The results indicated a significant indirect association between PSS and AB through MT, supporting H2. This finding is broadly consistent with related evidence. In sport settings, greater PSS was associated with higher MT among athletes [52]. A prospective study further showed that young elite athletes with higher MT reported lower levels of burnout and depressive symptoms under conditions of elevated stress [53]. Related evidence from university students also showed that resilience mediated the association between social support and AB [54]. Although resilience is conceptually distinct from MT, this finding provides complementary support for the broader resource-based perspective that social support may be associated with lower AB through internal psychological resources that facilitate adaptation to demanding conditions. The present findings extend this broader resource-based pattern to college athletes by suggesting that, under concurrent academic and athletic demands, MT may function as an internal psychological resource linking PSS to lower AB. From the COR perspective, external resources such as PSS may contribute to resource-gain processes that enhance internal psychological resources, enabling individuals to handle stress more effectively and sustain adaptive functioning [19]. Within this process, PSS may provide favorable social-resource conditions for the development and maintenance of MT. The confidence, constancy, and control reflected in MT may help college athletes remain committed to their academic and training goals and cope more adaptively with setbacks and uncertainty, thereby limiting sustained strain under concurrent demands and contributing to lower AB. Accordingly, universities may consider integrating MT-development components into structured psychological skills training and stress-coping interventions to help reduce the risk and severity of AB and support more balanced academic and athletic development.

### 4.3 The mediating role of athlete burnout

ATHB served as a significant mediator between PSS and AB. This result supports H3 and echoes earlier findings that PSS was associated with better mental health partly through lower levels of ATHB [55]. COR theory suggests that personal resources are finite. Under demanding athletic conditions, athletes may expend considerable psychological resources, leaving fewer available for academic responsibilities. This resource strain can undermine motivation and concentration while increasing fatigue and anxiety, ultimately raising the likelihood of AB [19]. Against this background, perceived support may provide athletes with external resources that help them manage sport-related demands and limit further resource strain. Consistent with this interpretation, prior research found that perceived support availability was negatively associated with specific dimensions of ATHB, while perceived emotional support buffered the positive association between stress and sport devaluation [27]. Furthermore, chronic burnout has been linked to higher cortisol concentrations and poorer executive functioning, including impairments in memory, attention, and cognitive performance [56]. When burnout experienced in the athletic domain is not effectively alleviated, its associated negative emotions and cognitive burden may extend into the academic domain and be associated with higher levels of AB. In college athletes, higher ATHB may therefore be associated with greater negative emotions and cognitive burden, which are also relevant to higher levels of AB. Thus, ATHB functions not only as a risk factor within the competitive sports context but also as a psychological drain that weakens individuals’ resource reserves for coping with academic stress, making it an important factor associated with AB [57]. This study identified a significant indirect association between PSS and AB through ATHB, highlighting the importance of considering ATHB under concurrent academic and athletic demands. This finding may inform future multicomponent support approaches that emphasize the early identification and management of ATHB, recovery enhancement, stress-management training, and coordinated academic and training demands among college athletes.

### 4.4 The serial mediating role of mental toughness and athlete burnout

This study further identified a significant serial indirect association between PSS and AB through MT and ATHB. Rather than directly replicating an established serial mediation model, this finding extends previous evidence concerning the adjacent associations among these constructs. Consistent with existing research, MT mediated the association between social support and ATHB [31], and ATHB was positively associated with AB [30]. The present findings extend this evidence by indicating that these adjacent associations may operate sequentially in linking external social resources to burnout in the academic domain. Based on the COR perspective of resource gain, conservation, and loss, the serial mediation found in this study can be understood as a process in which external social resources are linked to academic-domain depletion through internal MT and athletic-domain resource loss. Under dual academic and athletic demands, higher PSS may correspond to stronger MT, which in turn may be associated with lower ATHB. When athletic-domain resource loss is lower, athletes may retain more psychological and cognitive resources for academic tasks, thereby reporting lower levels of AB. Thus, PSS and MT may be linked to lower academic-domain depletion through lower athletic-domain resource loss. In this study, college athletes demonstrated high levels of MT, which may be associated with more effective self-regulation and a greater capacity to draw on PSS when managing emotional exhaustion in athletic contexts. This pattern may inform future efforts aimed at preserving resources across domains and addressing AB among college athletes.

### 4.5 The moderating role of cognitive reappraisal

This study revealed a path-specific moderating pattern of CR across the proposed associations with AB.

First, CR played a significant moderating role in the association between PSS and AB (supporting H5). The simple slope analysis showed that the protective association between PSS and lower AB was mainly observed among athletes with higher CR. This finding is consistent with previous research [33,34], and aligns with the Process Model of Emotion Regulation [58]. From a COR perspective, this pattern may reflect a resource-gain process whereby higher CR enables athletes to interpret and use perceived support more adaptively, which may help explain the stronger inverse association between PSS and AB observed at higher levels of CR. By contrast, athletes with lower CR may find it more difficult to cognitively reframe PSS as a positive resource for coping with academic stress, even when they perceive such support. This may explain why the negative association between PSS and AB was relatively weaker among these athletes. This conditional effect suggests that efforts to address AB among college athletes should not rely solely on increasing the availability of social support, but should also address athletes’ CR capacity. In addition, because PSS is inherently an interpersonal resource, future research could further examine whether interpersonal emotion-regulation strategies and PSS are jointly associated with lower levels of AB. Recent evidence among college students has shown that interpersonal emotion regulation and social self-efficacy play mediating roles in the association between autistic traits and depression [59], suggesting that interpersonal emotion-regulation processes may help explain how social resources are translated into better mental health outcomes. Accordingly, future studies could investigate whether interpersonal emotion regulation interacts with or complements CR in helping college athletes use perceived support in relation to emotional recovery and coping.

Secondly, CR significantly moderated the ATHB → AB path, supporting H7. Simple slope tests showed that the positive association between ATHB and AB was stronger at higher than at lower levels of CR. The serial indirect effect of PSS on AB through MT and ATHB also varied as a function of CR, supporting H8. Moreover, both the indirect effect through ATHB and the serial indirect effect through MT and ATHB were more negative at higher levels of CR, consistent with the stronger ATHB–AB association observed at higher CR. These findings indicate that CR may not function as a uniformly protective resource across burnout-related pathways. Several tentative explanations may account for this complex moderation pattern. First, CR may be adaptive under mild or moderate stress, but when burnout-related strain is severe, repeated cognitive reframing may become less effective or more cognitively costly. Athletes with higher CR may also be more sensitive to the mismatch between their reframing efforts and their declining athletic or academic adjustment, making the association between burnout experiences and AB more pronounced. In addition, high-CR athletes may over-rely on cognitive reframing while engaging less in problem-focused coping, such as adjusting training load, seeking instrumental support, or improving recovery routines. When ATHB accumulates, this over-reliance may contribute to strategy exhaustion. Third, among those reporting elevated ATHB, the boundary between CR and rumination may become blurred. In this context, higher self-reported CR may partly reflect repetitive cognitive processing of burnout-related negative experiences rather than solely adaptive reinterpretation, which could help explain the stronger positive association between ATHB and AB at higher levels of CR. Overall, the moderating role of CR may be path-specific and context-dependent. In this study, both the negative PSS–AB association and the positive ATHB–AB association were stronger at higher levels of CR. Therefore, CR should not be interpreted as having a uniformly buffering role across stress-related pathways. This finding also has practical implications. Because the ATHB–AB association was stronger among athletes with higher CR, CR training should not be treated as a stand-alone solution when athletes already experience elevated burnout.

However, within the indirect pathway of “PSS → MT → AB”, CR did not significantly moderate the link between MT and AB, which is inconsistent with H6. This finding indicates that the magnitude of the negative association between MT and AB did not differ significantly across levels of CR. A partially analogous finding was reported among psychiatric nurses, where CR did not significantly moderate the association between natural expression and burnout [60]. These two findings suggest that its moderating role may be context-dependent rather than universally applicable.

Overall, the moderating role of CR appears to be path-specific and context-dependent, suggesting that intervention design should consider the relative magnitude of effects across pathways. Although the AB equation showed a relatively strong overall explanatory effect, local f^2^ values indicated that ATHB had the largest unique association with AB, whereas the unique associations of PSS, MT, CR, and the interaction terms were small. Thus, ATHB may be a priority area for interventions addressing AB among college athletes, while PSS enhancement, MT development, and CR training may serve as complementary components of a multicomponent intervention. From a practical perspective, colleges, athletic departments, teachers, coaches, and counselors may consider combining CR-related training with problem-focused and context-focused support, such as academic guidance, emotional support, ATHB monitoring, training-load adjustment, recovery management, instrumental academic support, and academic–athletic role coordination. This multicomponent approach may help athletes reinterpret academic stress and support-related information while also reducing contextual sources of burnout, thereby making external support more likely to function as an adaptive coping resource. These findings provide preliminary evidence for targeted intervention design and should be further validated through longitudinal or experimental studies.

### 4.6 Limitations and future directions

While this study systematically examined the interrelations among PSS, MT, ATHB, CR, and AB in college athletes—and identified significant indirect associations through MT and ATHB as well as path-specific moderation by CR —several limitations should be noted. First, this study adopted a cross-sectional design, which precludes strict causal inferences and makes it difficult to reveal the potential developmental dynamics among the variables. Therefore, the proposed PSS → MT → ATHB → AB pathway should be understood as a theory-driven statistical model rather than as evidence of a strict causal chain. Other alternative pathways are also theoretically plausible. Future studies could adopt longitudinal, cross-lagged, or experimental designs to further examine the temporal ordering among variables and compare the robustness of different competing models. Second, this study used convenience sampling, and participants were mainly recruited from Chinese university sports teams, which may limit the representativeness and generalizability of the findings. Future research should include more diverse and representative samples and further examine whether the model applies across different sports, competitive levels, and cultural contexts. Third, all variables were assessed using self-report measures, which may have introduced social desirability bias and common method variance. Harman’s single-factor test did not indicate that a single factor dominated the overall covariance structure, whereas the ULMC analysis suggested nontrivial method-related shared covariance. Therefore, the potential influence of common method variance on some of the estimated associations cannot be completely excluded. Future studies could use multi-informant data, behavioral indicators, temporal separation, or theoretically appropriate marker variables to further evaluate the robustness of the findings. Fourth, the relatively high correlation between PSS and CR, together with the limited empirical separation between the method factor and the substantive CR factor in the ULMC analysis, may affect the stability and interpretation of estimates involving CR. Although the collinearity diagnostics did not indicate severe multicollinearity, some conceptual or measurement overlap between PSS and CR cannot be ruled out. Accordingly, the moderation findings involving CR should be interpreted cautiously and replicated in independent samples using alternative measures to assess their robustness. Finally, this study examined CR as the sole intrapersonal emotion-regulation moderator, whereas PSS represents an interpersonal resource. The current model may therefore not fully capture how interpersonal emotion-regulation strategies may help college athletes mobilize perceived support from others and thereby strengthen its association with lower burnout. Future studies should incorporate interpersonal emotion regulation and examine whether it complements or interacts with PSS in relation to AB.

## 5. Conclusion

PSS was significantly and negatively associated with AB among college athletes. Path analyses identified a direct negative association and three significant indirect associations: separately through MT and ATHB and sequentially through MT and ATHB. These theory-consistent indirect associations do not establish temporal ordering or causality. CR showed a path-specific moderating pattern: at higher levels of CR, the negative PSS–AB association was stronger, but the positive ATHB–AB association was also stronger, whereas the MT–AB association did not differ significantly across CR levels. Thus, CR should not be interpreted as having a uniformly buffering role. These findings suggest that future support programs may consider enhancing PSS, fostering MT, and identifying and addressing ATHB, while incorporating CR-related training cautiously and with attention to the specific pathway involved. The effectiveness and boundary conditions of such approaches require further evaluation through longitudinal or experimental studies.

## Supporting information

S1 Table CFA Alternative Model Comparisons and ULMC Test. (DOCX)

S2 Table Construct Reliability, Convergent Validity and Discriminant Validity. (DOCX)

S3 Table Heterotrait-Monotrait Ratios (HTMT) among Constructs. (DOCX)

S4 Table Regression Coefficients for the Serial Mediation Model. (DOCX)

S5 Table Regression Coefficients of Covariates in the Moderated Serial Mediation Model. (DOCX)
