# Supplementary material for: The impact of perceived social support on college athletes’ academic burnout: The moderating role of cognitive reappraisal within a serial mediation model
Source: PLoS One. 2026 Oct 22;21(10):e0361222. doi: 10.1371/journal.pone.0361222 (PMC13690224; doi:10.1371/journal.pone.0361222)
Supplement: Supplementary file 1 [file pone.0361222.s006.pdf]

PEER REVIEW REPORT

Manuscript Number: PONE-D-26-07482

Title: The Impact of Perceived Social Support on College Athletes' Academic Burnout: The Moderating Role of Cognitive Reappraisal within a Serial Mediation Model

Overall Assessment

This study investigates how perceived social support (PSS) influences academic burnout (AB) among college athletes in China, with psychological resilience (PR) and athlete burnout (ATHB) proposed as serial mediators and cognitive reappraisal (CR) as a moderator. The research topic is practically relevant, the sample size is substantial (N = 1,918), and the analytical framework carries some theoretical novelty. However, after a thorough reading of the manuscript, I have identified a number of concerns regarding the transparency of data disclosure, internal consistency of reported results, construct validity, statistical methodology, and the theoretical depth of the discussion. These concerns, detailed below, require substantive revision before this manuscript can be considered for publication.

Specific Comments

Q1. The authors acknowledge in Lines 206–209 that the dataset used in the present study was collected from the same cohort of college athletes as their previously published study (Reference 33: Xu et al., 2025, Frontiers in Psychology). The earlier study and the present manuscript appear to share several closely related elements: academic burnout serves as the dependent variable in both papers, athlete burnout is a key variable in both, and emotion regulation/cognitive reappraisal is involved in both analytical frameworks.

I do not suggest that the use of the same cohort across multiple studies is inherently inappropriate. However, when the same participant cohort and closely related psychological variables are used across separate manuscripts, the relationship between the studies should be disclosed with greater transparency. At present, the manuscript addresses this issue only briefly, stating that the research objectives, variable selection, and analytical framework are different. This statement is helpful, but it does not provide enough detail for readers to evaluate the degree of overlap and the distinct contribution of the present manuscript.

I recommend that the authors add a brief clarification in the Methods section, or in the supplementary materials if space is limited, explaining the relationship between the present study and the previously published paper. This clarification should specify which variables overlap across the two studies, whether the relevant measures were collected during the same survey administration, and what distinct theoretical or analytical contribution the present manuscript makes beyond the prior publication.

Q2. Lines 258–268 describe the Sports Mental Toughness Questionnaire (SMTQ), including its origin, dimensional structure, scoring method, and internal reliability. However, in the correlation matrix and subsequent mediation and moderation models, the corresponding construct is labeled as psychological resilience (PR), rather than mental toughness. This creates conceptual and methodological ambiguity.

The authors should explicitly state whether the SMTQ total score was used as the operational indicator of PR. If so, this operational decision requires clear justification, because mental toughness and psychological resilience are related but not identical constructs. The Methods section should explain why a measure originally designed to assess sports mental toughness can validly represent psychological resilience in the present model. The Results section should also make the naming consistent, so that readers can clearly understand whether the construct being tested is mental toughness, psychological resilience, or a theoretically justified proxy for resilience.

At present, the manuscript appears to move from the measurement label " sports mental toughness" to the analytical label "psychological resilience" without sufficient explanation. This should be clarified before readers can properly evaluate the validity of the mediation model.

Q3. Related to the previous point, the manuscript repeatedly frames the relevant mediator as "psychological resilience," citing literature that defines resilience as the capacity for recovery, adaptation, and sustained functioning under adversity. However, the actual measurement instrument appears to be the SMTQ, which was designed to assess sports mental toughness rather than resilience per se.

Although mental toughness and psychological resilience overlap in sport psychology, they should not be treated as fully interchangeable without theoretical or empirical justification. Resilience generally emphasizes recovery, adaptation, and positive functioning after adversity or stress exposure, whereas mental toughness more strongly emphasizes confidence, constancy, persistence, and perceived control under pressure. These conceptual differences matter because the manuscript interprets the mediator as an internal psychological resource that is built through

perceived social support and then reduces athlete burnout and academic burnout.

I recognize that the data have already been collected and that it may not be feasible to re-administer an alternative resilience measure, such as the Connor-Davidson Resilience Scale, to the same 1,918 participants. Therefore, I do not require the authors to replace the measure. However, the authors should address this construct-validity issue in three specific ways.

First, in the Methods section, the authors should add a paragraph justifying the use of the SMTQ to operationalize psychological resilience. This justification should cite sport psychology literature showing the conceptual and empirical overlap between mental toughness and resilience, especially in athlete populations.

Second, in the Limitations section, the authors should explicitly acknowledge that the SMTQ was not originally designed to measure psychological resilience per se. The authors should explain that this operationalization choice may affect the interpretation of the PR-related findings and that future research using dedicated resilience measures would be necessary to verify the robustness of the current mediation results.

Third, the authors should provide additional evidence that the construct measured by the SMTQ is empirically distinguishable from the other constructs in the model. This is especially important given the relatively high correlations among some self-report variables, such as the correlation between PR and CR. A confirmatory factor analysis, or another appropriate test of discriminant validity, would help demonstrate that the SMTQ-based mediator captures a distinct construct rather than reflecting a broader general psychological adjustment factor.

Without these clarifications, the current manuscript risks overstating the evidence for a psychological resilience mechanism when the empirical measure may more accurately reflect sports mental toughness. This does not necessarily invalidate the findings, but it does require a more cautious and transparent interpretation.

Q4. The study employs five self-report instruments simultaneously, with notably high inter-correlations among several variables. For example, Table 2 reports relatively high correlations between PSS and CR ($r = 0.658$) and between PR and CR ($r = 0.560$). Under these circumstances, it would be important to provide CFA results, or other appropriate evidence of discriminant validity, to confirm that the five constructs are empirically distinguishable.

At present, the manuscript reports internal consistency coefficients and bivariate correlations, but it does not provide sufficient evidence that the measured constructs are distinct from one another. The particularly high correlation between PSS and CR raises the question of whether these two variables may partly reflect overlapping aspects of a broader positive psychological adjustment

factor. I recommend that the authors report additional evidence of construct distinctiveness, such as confirmatory factor analysis, comparison of alternative factor models, AVE-based discriminant validity, HTMT, or another appropriate approach.

Q5. Table 3 reports the 95% confidence interval for the PSS → ATHB → AB indirect effect as [−0.065, −0.028], whereas Line 312 in the text reports the same result as [−0.064, −0.027]. Both the lower and upper bounds differ. Although the discrepancy is numerically small, reporting different values for the same result within the same manuscript undermines the credibility of the data. The authors should verify the original PROCESS output and ensure consistency throughout.

Q6. Figure 2 presents standardized path coefficients from the serial mediation model (Model 6), such as PR → AB = −0.150 and ATHB → AB = 0.327. These values differ from those in Table 4, which reports Model 89 coefficients (−0.108 and 0.302, respectively). I understand this discrepancy arises because the two models include different sets of predictors, which is expected. However, the authors never provide the full regression coefficient table for Model 6. Only the indirect effects appear in Table 3, leaving the path coefficients shown in Figure 2 unverifiable against any tabulated data. I recommend adding a complete Model 6 regression table in the main text or supplementary materials.

Q7. Table 4 reports the p-value for the PSS × CR interaction term as $p = 0.010$, while Line 327 describes the same result as "$p < 0.05$." The manuscript alternates between exact p-values and inequality notation throughout. I recommend adopting a uniform reporting standard.

Q8. When the correlation between an independent variable and a moderator reaches 0.66, interaction term estimates may become unstable. The authors note in Lines 283–284 that variables were mean-centered prior to computing interaction terms, which is appropriate. However, mean-centering only reduces collinearity between the interaction term and the constituent main effects; it does not address collinearity between the main effects themselves. I recommend reporting VIF (Variance Inflation Factor) values for each predictor across all models.

Q9. Lines 287–294 report that the first unrotated factor explained 23.769% of the variance, below the 40% threshold. However, Harman's test has been widely criticized in the methodological literature for its low sensitivity and limited statistical power. I recommend that the authors at minimum acknowledge this limitation, and ideally supplement the test with a more robust approach, such as adding a common method factor within the CFA framework.

Q10. The moderated serial mediation model tested in the manuscript specifies CR as a moderator of paths leading to AB, including the PSS → AB, PR → AB, and ATHB → AB paths. This specification is theoretically plausible, but the manuscript does not sufficiently justify why CR

was modeled only at these later-stage paths rather than at earlier stages of the serial chain, such as PSS → PR, PSS → ATHB, or PR → ATHB.

Given that the Introduction describes CR as a factor that may influence the transformation of external support into internal psychological resources, one might also expect CR to moderate earlier resource-transformation paths. I recommend that the authors provide a clearer theoretical rationale for the chosen PROCESS Model 89 specification and acknowledge that alternative moderated mediation models could test different boundary conditions within the serial pathway.

Q11. Table 4 and Figure 5 show that CR positively moderates the ATHB → AB path ($\beta = 0.078$, $p < 0.001$). The simple slope analysis indicates that the association between ATHB and AB is stronger among athletes with higher CR ($\beta = 0.380$) than among those with lower CR ($\beta = 0.223$). In other words, higher CR is associated with a stronger ATHB–AB link, rather than a weaker one.

This finding complicates, and partly contradicts, the manuscript's general framing of CR as a uniformly protective factor. Although CR appears to strengthen the protective association between PSS and AB, its moderating role in the ATHB → AB path operates in the opposite direction from what a simple protective-factor interpretation would predict.

The authors briefly touch on this result in Lines 476–479 but do not meaningfully engage with its theoretical implications. I believe this finding warrants a more thorough discussion. Possible explanations might include: (a) cognitive reappraisal may be effective under mild stress but may fail or even backfire when athletic burnout reaches severe levels, with high-CR individuals becoming more acutely aware of their deteriorating state; (b) high-CR individuals may over-rely on reappraisal strategies while neglecting problem-focused coping, leading to strategy exhaustion when burnout accumulates; or (c) there may be blurred boundaries between reappraisal and rumination, such that some individuals' "reappraisal" effectively constitutes repeated cognitive processing of negative experiences. These possibilities deserve serious discussion rather than a cursory mention.

In its current form, the Discussion risks overstating the protective role of CR and underinterpreting a theoretically important, counterintuitive moderation effect.

Q12. The simple slope analysis for the PSS × CR interaction suggests that, among athletes with lower CR, the protective association between PSS and AB is weaker and does not reach conventional statistical significance (effect = −0.058, $p = 0.066$). By contrast, the association is stronger and statistically significant among athletes with higher CR (effect = −0.162, $p < 0.001$).

This pattern has important implications for intervention design. It suggests that enhancing social support alone may be less effective for athletes who have lower levels of cognitive reappraisal, and that social support interventions may need to be combined with emotion regulation training to produce more consistent benefits for reducing academic burnout.

However, the manuscript's discussion of this point is too brief. I recommend that the authors elaborate on the practical implications of this conditional effect, especially with regard to how universities, coaches, counselors, and academic support staff might design interventions that integrate social support enhancement with emotion regulation training.

Q13. The AB equation in Table 4 explains 25.9% of the variance in academic burnout. While this represents a meaningful proportion of explained variance in psychological research, the manuscript does not report individual predictor effect sizes or discuss the practical magnitude of the observed associations. I recommend that the authors supplement the statistical significance tests with effect size indicators where appropriate, such as semi-partial effects, standardized effect interpretation, or f^2 values. More importantly, the Discussion should clarify what the observed effects mean for practical intervention design. For example, the authors should explain whether the effects of PSS, ATHB, and CR are sufficiently large to support targeted intervention programs, or whether the findings should be interpreted as preliminary evidence requiring further validation.

Q14. The authors successively draw upon the stress-buffering model (Line 91), Conservation of Resources theory (Line 106), the process model of emotion regulation (Line 151), and later in the Discussion introduce the Job Demands–Resources model (Line 448) and self-regulation theory (Line 470). While each framework has some relevance, the current presentation lacks a unifying logical thread and reads more like a list of theories than an integrated framework. I recommend anchoring the paper in COR theory as the primary framework and clearly positioning the other theories in supporting roles.

Q15. The proposed sequence assumes that social support builds resilience, which in turn reduces athletic burnout, and the reduction in athletic burnout then lowers academic burnout. However, under a cross-sectional design, alternative causal orderings are equally plausible. For example, ATHB → PR → AB (athletic burnout erodes resilience, which then exacerbates academic burnout) is theoretically defensible. I recommend that the authors provide more thorough theoretical justification for the chosen ordering in the Introduction, and indicate whether any competing models were tested. If not, this should be acknowledged in the Limitations section.

Q16. H7 reads "CR moderates the relationship between ABTH and AB." The abbreviation

should be "ATHB," not "ABTH." Please also check the x-axis label of Figure 5 and all related figure labels for abbreviation consistency, and correct this issue throughout the manuscript.

Q17. Lines 386–389 discuss how PSS may modulate the sympathetic nervous system and the HPA axis to reduce physiological stress responses. However, the present study collected no physiological data whatsoever. While citing relevant literature as theoretical background is acceptable, the current phrasing risks misleading readers into thinking these mechanisms are findings of the present study. I recommend adjusting the wording to clearly mark these as speculative interpretations based on prior literature.

Q18. The Limitations discussion (Lines 496-509) covers cross-sectional design, self-report instruments, and the non-significant PR × CR interaction, which is reasonable. However, it omits several points worth discussing: (a) the sample was recruited through convenience sampling from Chinese university sports teams, and the representativeness of the sample may be limited, particularly given the uneven distribution of sport types and athlete levels; (b) using the SMTQ to operationalize psychological resilience may introduce construct validity concerns (see Q3); and (c) the high collinearity between PSS and CR ($r = 0.658$) may affect the stability of interaction term estimates (see Q8).

Q19. The reference list uses journal abbreviations for some entries and full journal names for others; some entries include complete page information while others provide only article numbers; and the formatting of translated Chinese-language references is inconsistent with other entries. Please revise in accordance with PLOS ONE reference formatting requirements.

Q20. After a careful reading of the Introduction, I find that the empirical grounding for the hypotheses is uneven. H1 and H2 are reasonably well supported by cited literature. H3, however, relies on the ATHB → AB spillover assumption, for which the only cited evidence is the authors' own prior work (Reference 33, Line 142: "our preliminary work suggests"). A single self-citation is insufficient to anchor a mediation hypothesis; the authors should supplement this with independent evidence on cross-domain burnout transfer.

H4, the core serial mediation hypothesis, lacks direct empirical precedent. No cited study has tested a sequential PSS → PR → ATHB → AB pathway, and the critical PR → ATHB link receives only a brief mention at Lines 136–138 (Reference 29), citing a correlational association rather than a mediation relationship. A four-variable serial mediation hypothesis requires stronger literature support than what is currently provided.

Most critically, the moderation hypotheses H5 through H8 (Lines 176–179) suffer from a systematic logical gap. The authors' argument in Lines 154–161 proceeds as follows: CR is

related to AB (Reference 36), CR is related to resilience (Reference 37), and CR is related to ATHB (Reference 38). From these bivariate associations, the authors derive hypotheses that CR moderates specific paths (PSS → AB, PR → AB, ATHB → AB, and the serial indirect effect). However, "CR correlates with variable X" does not logically entail "CR moderates the path from X to Y." Correlation and moderation are distinct statistical and conceptual relationships. Furthermore, Reference 36 actually examines CR as a mediator between social support and academic burnout, not as a moderator. Using a mediation finding to justify a moderation hypothesis conflates two different theoretical mechanisms. I recommend that the authors either locate prior studies that have specifically tested CR (or related emotion regulation strategies) as a moderator of burnout pathways, or reframe the moderation hypotheses as more exploratory in nature and adjust the language in the Introduction accordingly.
