# Supplementary material for: The impact of perceived social support on college athletes’ academic burnout: The moderating role of cognitive reappraisal within a serial mediation model
Source: PLoS One. 2026 Oct 22;21(10):e0361222. doi: 10.1371/journal.pone.0361222 (PMC13690224; doi:10.1371/journal.pone.0361222)
Supplement: Supplementary file 2 [file pone.0361222.s001.docx]

**S1 Table.** CFA Alternative Model Comparisons and ULMC Test (N = 1,918).

| Model | χ² | df | CFI | TLI | RMSEA | SRMR |
| --- | --- | --- | --- | --- | --- | --- |
| Five-factor model  (PSS, MT, ATHB, CR, AB) | 1127.001 | 125 | 0.952 | 0.941 | 0.065 | 0.050 |
| Four-factor model  (PSS+MT, ATHB, CR, AB) | 2063.628 | 129 | 0.907 | 0.889 | 0.088 | 0.072 |
| Three-factor model  (PSS+MT+ATHB, CR, AB) | 4448.388 | 132 | 0.792 | 0.759 | 0.131 | 0.098 |
| Two-factor model  (PSS+MT+ATHB+CR, AB) | 5987.710 | 134 | 0.718 | 0.678 | 0.151 | 0.106 |
| One-factor model  (PSS+MT+ATHB+CR+AB) | 7998.226 | 135 | 0.621 | 0.571 | 0.174 | 0.119 |
| Five-factor model with ULMC  (PSS, MT, ATHB, CR, AB, M) | 701.894 | 107 | 0.971 | 0.959 | 0.054 | 0.036 |

Note: PSS = Perceived Social Support, MT = Mental Toughness, ATHB = Athlete Burnout, CR = Cognitive Reappraisal, AB = Academic Burnout, M = an unmeasured latent method factor.
