# Supplementary material for: The impact of perceived social support on college athletes’ academic burnout: The moderating role of cognitive reappraisal within a serial mediation model
Source: PLoS One. 2026 Oct 22;21(10):e0361222. doi: 10.1371/journal.pone.0361222 (PMC13690224; doi:10.1371/journal.pone.0361222)
Supplement: Supplementary file 3 [file pone.0361222.s002.docx]

**S2 Table.** Construct Reliability, Convergent Validity and Discriminant Validity (N = 1,918).

| Construct | CRc | AVE | PSS | ATHB | MT | CR | AB |
| --- | --- | --- | --- | --- | --- | --- | --- |
| PSS | 0.916 | 0.784 | **0.885** |  |  |  |  |
| ATHB | 0.866 | 0.684 | −0.385^***^ | **0.827** |  |  |  |
| MT | 0.770 | 0.533 | 0.658^***^ | −0.483^***^ | **0.730** |  |  |
| CR | 0.895 | 0.588 | 0.723^***^ | −0.352^***^ | 0.675^***^ | **0.767** |  |
| AB | 0.837 | 0.639 | −0.342^***^ | 0.485^***^ | −0.427^***^ | −0.335^***^ | **0.799** |

Note: CRc = composite reliability; AVE = average variance extracted. The bold values on the diagonal represent the square root of AVE. PSS = Perceived Social Support, MT = Mental Toughness, ATHB = Athlete Burnout, CR = Cognitive Reappraisal, AB = Academic Burnout, ***p < 0.001.
