# Supplementary material for: The impact of perceived social support on college athletes’ academic burnout: The moderating role of cognitive reappraisal within a serial mediation model
Source: PLoS One. 2026 Oct 22;21(10):e0361222. doi: 10.1371/journal.pone.0361222 (PMC13690224; doi:10.1371/journal.pone.0361222)
Supplement: Supplementary file 4 [file pone.0361222.s003.docx]

**S3 Table.** Heterotrait–Monotrait Ratios (HTMT) among Constructs (N = 1,918).

| Construct | PSS | ATHB | MT | CR | AB |
| --- | --- | --- | --- | --- | --- |
| PSS | — |  |  |  |  |
| ATHB | 0.408 | — |  |  |  |
| MT | 0.685 | 0.577 | — |  |  |
| CR | 0.727 | 0.379 | 0.689 | — |  |
| AB | 0.380 | 0.513 | 0.472 | 0.380 | — |

Note: PSS = Perceived Social Support, MT = Mental Toughness, ATHB = Athlete Burnout, CR = Cognitive Reappraisal, AB = Academic Burnout.
