# Supplementary material for: The impact of perceived social support on college athletes’ academic burnout: The moderating role of cognitive reappraisal within a serial mediation model
Source: PLoS One. 2026 Oct 22;21(10):e0361222. doi: 10.1371/journal.pone.0361222 (PMC13690224; doi:10.1371/journal.pone.0361222)
Supplement: Supplementary file 5 [file pone.0361222.s004.docx]

**S4 Table.** Regression Coefficients for the Serial Mediation Model (N = 1,918).

| Antecedents | Consequent | | | | | | | | | | | | | |
| --- | --- | --- | --- | --- | --- | --- | --- | --- | --- | --- | --- | --- | --- | --- |
|  | M1 (MT) | | | |  | M2 (ATHB) | | | |  | Y (AB) | | | |
|  | Coeff. | SE | *p* | 95% CI |  | Coeff. | SE | *p* | 95% CI |  | Coeff. | SE | *p* | 95% CI |
| Constant | 0.038 | 0.276 | 0.890 | [−0.503, 0.579] |  | −0.784 | 0.295 | 0.008 | [−1.363, −0.205] |  | 0.085 | 0.294 | 0.774 | [−0.493, 0.662] |
| PSS | 0.566 | 0.019 | < 0.001 | [0.529, 0.603] |  | −0.133 | 0.025 | < 0.001 | [−0.181, −0.085] |  | −0.122 | 0.025 | < 0.001 | [−0.170, −0.073] |
| MT |  |  |  |  |  | −0.373 | 0.025 | < 0.001 | [−0.421, −0.324] |  | −0.154 | 0.026 | < 0.001 | [−0.205, −0.103] |
| ATHB |  |  |  |  |  |  |  |  |  |  | 0.328 | 0.023 | < 0.001 | [0.283, 0.373] |
| Gender  (ref: Male) |  |  |  |  |  |  |  |  |  |  |  |  |  |  |
| Female | −0.153 | 0.040 | < 0.001 | [−0.232, −0.074] |  | −0.135 | 0.043 | 0.002 | [−0.220, −0.050] |  | 0.034 | 0.043 | 0.433 | [−0.051, 0.119] |
| Age | −0.002 | 0.014 | 0.899 | [−0.029, 0.025] |  | 0.042 | 0.015 | 0.005 | [0.013, 0.071] |  | −0.007 | 0.015 | 0.619 | [−0.036, 0.022] |
| Family Structure  (ref: Intact Family) |  |  |  |  |  |  |  |  |  |  |  |  |  |  |
| Reconstituted Family | 0.001 | 0.091 | 0.994 | [−0.177, 0.178] |  | 0.028 | 0.097 | 0.771 | [−0.162, 0.218] |  | 0.007 | 0.097 | 0.943 | [−0.182, 0.196] |
| Single-Parent Family | 0.138 | 0.068 | 0.042 | [0.005, 0.272] |  | −0.110 | 0.073 | 0.132 | [−0.252, 0.033] |  | 0.158 | 0.073 | 0.030 | [0.016, 0.300] |
| Orphaned Family | −0.062 | 0.280 | 0.825 | [−0.611, 0.487] |  | −0.029 | 0.300 | 0.924 | [−0.616, 0.559] |  | −0.334 | 0.298 | 0.263 | [−0.919, 0.251] |
| Residence (ref: Urban) |  |  |  |  |  |  |  |  |  |  |  |  |  |  |
| Rural | 0.035 | 0.047 | 0.459 | [−0.058, 0.127] |  | 0.034 | 0.050 | 0.500 | [−0.065, 0.133] |  | 0.006 | 0.050 | 0.905 | [−0.093, 0.105] |
| Township | −0.120 | 0.063 | 0.055 | [−0.243, 0.003] |  | 0.189 | 0.067 | 0.005 | [0.057, 0.321] |  | 0.038 | 0.067 | 0.573 | [−0.094, 0.169] |
| County-Level City | 0.013 | 0.056 | 0.815 | [−0.097, 0.123] |  | 0.045 | 0.060 | 0.458 | [−0.073, 0.162] |  | 0.033 | 0.060 | 0.582 | [−0.084, 0.150] |
| Sport Item  (ref: Combat Sports) |  |  |  |  |  |  |  |  |  |  |  |  |  |  |
| Ball Sports | 0.008 | 0.046 | 0.864 | [−0.082, 0.098] |  | −0.084 | 0.049 | 0.088 | [−0.181, 0.012] |  | −0.027 | 0.049 | 0.579 | [−0.124, 0.069] |
| Track and Field | 0.130 | 0.062 | 0.035 | [0.009, 0.251] |  | 0.130 | 0.066 | 0.050 | [0.000, 0.259] |  | 0.047 | 0.066 | 0.473 | [−0.082, 0.177] |
| Aquatic Sports | −0.224 | 0.115 | 0.051 | [−0.449, 0.001] |  | −0.133 | 0.123 | 0.278 | [−0.374, 0.108] |  | 0.129 | 0.122 | 0.292 | [−0.111, 0.369] |
| Other Sports | 0.205 | 0.104 | 0.050 | [0.000, 0.410] |  | −0.063 | 0.112 | 0.575 | [−0.282, 0.157] |  | 0.250 | 0.111 | 0.025 | [0.031, 0.469] |
| Sport Level  (ref: National Level-2 Athlete) |  |  |  |  |  |  |  |  |  |  |  |  |  |  |
| International-Level Elite Athlete | −0.041 | 0.170 | 0.808 | [−0.375, 0.292] |  | 0.057 | 0.182 | 0.752 | [−0.300, 0.415] |  | −0.093 | 0.181 | 0.608 | [−0.449, 0.263] |
| National-Level Elite Athlete | 0.099 | 0.097 | 0.306 | [−0.091, 0.290] |  | 0.058 | 0.104 | 0.575 | [−0.146, 0.262] |  | 0.024 | 0.104 | 0.817 | [−0.179, 0.227] |
| National Level-1 Athlete | 0.068 | 0.044 | 0.117 | [−0.017, 0.154] |  | −0.072 | 0.047 | 0.125 | [−0.163, 0.020] |  | 0.058 | 0.047 | 0.216 | [−0.034, 0.149] |
| Model Statistics | R² = 0.335 F(16, 1901) = 59.867, p < 0.001 | | | |  | R² = 0.239 F(17, 1900) = 35.037, p < 0.001 | | | |  | R² = 0.246 F(18, 1899) = 34.361, p < 0.001 | | | |

Note: PSS = Perceived Social Support, MT = Mental Toughness, ATHB = Athlete Burnout, CR = Cognitive Reappraisal, AB = Academic Burnout. All models controlled for gender, age, family structure, residence, sport item, and sport level. Gender was binary coded, whereas multicategorical covariates were dummy coded using k−1 indicator variables, with male, intact family, urban residence, combat sports, and national level-2 athlete as reference categories.
