# Supplementary material for: The impact of perceived social support on college athletes’ academic burnout: The moderating role of cognitive reappraisal within a serial mediation model
Source: PLoS One. 2026 Oct 22;21(10):e0361222. doi: 10.1371/journal.pone.0361222 (PMC13690224; doi:10.1371/journal.pone.0361222)
Supplement: Supplementary file 6 [file pone.0361222.s005.docx]

**S5 Table.** Regression Coefficients of Covariates in the Moderated Serial Mediation Model (N = 1,918).

| Antecedents | Consequent | | | | | | | | | | |
| --- | --- | --- | --- | --- | --- | --- | --- | --- | --- | --- | --- |
|  | M1 (MT) | | |  | M2 (ATHB) | | |  | Y (AB) | | |
|  | β | SE | *p* |  | β | SE | *p* |  | β | SE | *p* |
| Gender (ref: Male) |  |  |  |  |  |  |  |  |  |  |  |
| Female | −0.153 | 0.040 | < 0.001 |  | −0.135 | 0.043 | 0.002 |  | 0.038 | 0.043 | 0.380 |
| Age | −0.002 | 0.014 | 0.899 |  | 0.042 | 0.015 | 0.005 |  | −0.005 | 0.015 | 0.716 |
| Family Structure (ref: Intact Family) |  |  |  |  |  |  |  |  |  |  |  |
| Reconstituted Family | 0.001 | 0.091 | 0.994 |  | 0.028 | 0.097 | 0.771 |  | 0.024 | 0.095 | 0.804 |
| Single-Parent Family | 0.138 | 0.068 | 0.042 |  | −0.110 | 0.073 | 0.132 |  | 0.137 | 0.072 | 0.057 |
| Orphaned Family | −0.062 | 0.280 | 0.825 |  | −0.029 | 0.300 | 0.924 |  | −0.351 | 0.296 | 0.235 |
| Residence (ref: Urban) |  |  |  |  |  |  |  |  |  |  |  |
| Rural | 0.035 | 0.047 | 0.459 |  | 0.034 | 0.050 | 0.500 |  | 0.010 | 0.050 | 0.841 |
| Township | −0.120 | 0.063 | 0.055 |  | 0.189 | 0.067 | 0.005 |  | 0.035 | 0.066 | 0.598 |
| County-Level City | 0.013 | 0.056 | 0.815 |  | 0.045 | 0.060 | 0.458 |  | 0.038 | 0.059 | 0.525 |
| Sport Item (ref: Combat Sports) |  |  |  |  |  |  |  |  |  |  |  |
| Ball Sports | 0.008 | 0.046 | 0.864 |  | −0.084 | 0.049 | 0.088 |  | −0.028 | 0.049 | 0.559 |
| Track and Field | 0.130 | 0.062 | 0.035 |  | 0.130 | 0.066 | 0.050 |  | 0.050 | 0.065 | 0.445 |
| Aquatic Sports | −0.224 | 0.115 | 0.051 |  | −0.133 | 0.123 | 0.278 |  | 0.115 | 0.121 | 0.342 |
| Other Sports | 0.205 | 0.104 | 0.050 |  | −0.063 | 0.112 | 0.575 |  | 0.247 | 0.110 | 0.025 |
| Sport Level (ref: National Level-2 Athlete) |  |  |  |  |  |  |  |  |  |  |  |
| International-Level Elite Athlete | −0.041 | 0.170 | 0.808 |  | 0.057 | 0.182 | 0.752 |  | 0.019 | 0.180 | 0.914 |
| National-Level Elite Athlete | 0.099 | 0.097 | 0.306 |  | 0.058 | 0.104 | 0.575 |  | 0.037 | 0.102 | 0.715 |
| National Level-1 Athlete | 0.068 | 0.044 | 0.117 |  | −0.072 | 0.047 | 0.125 |  | 0.066 | 0.046 | 0.152 |

Note: MT = Mental Toughness, ATHB = athlete burnout, AB = academic burnout. Age was entered as a continuous covariate. Gender and multicategorical variables (family structure, residence, sport item, and sport level) were dummy coded using k−1 indicator variables. Reference categories were male, intact family, urban residence, combat sports, and national level-2 athlete, respectively.
