# Supplementary material for: The impact of perceived social support on college athletes’ academic burnout: The moderating role of cognitive reappraisal within a serial mediation model
Source: PLoS One. 2026 Oct 22;21(10):e0361222. doi: 10.1371/journal.pone.0361222 (PMC13690224; doi:10.1371/journal.pone.0361222)
Supplement: Supplementary file 7 [file pone.0361222.s007.docx]

**Response to Editor**

Dear Editor,

First, we would like to sincerely thank you for your time and effort in handling our manuscript, as well as for granting us sufficient time to complete the revisions. We have carefully revised the manuscript according to the reviewers’ comments and have provided detailed point-by-point responses to each reviewer’s comments below.

To facilitate your and the reviewers’ evaluation, we have uploaded two versions of the revised manuscript: one with tracked changes and one clean version without tracked changes. We hope that the revised manuscript and our responses adequately address the reviewers’ concerns. If any further revisions or clarifications are needed, we would be very willing to make additional improvements.

Sincerely,

The Authors

**Response to Reviewer 1**

Dear Reviewer,

We sincerely thank you for your positive evaluation of our work and for your professional, constructive, and detailed comments. We greatly appreciate the time and effort you devoted to reviewing our manuscript. We believe that your insightful suggestions have helped us substantially improve the quality, clarity, and rigor of the manuscript.

We have carefully considered each of your comments and have revised the manuscript accordingly. Detailed point-by-point responses are provided below.

Sincerely,

The Authors

**Q1:** appreciate the authors' transparency in disclosing that the dataset used in this study was collected from the same cohort as their recently published research. However, upon reviewing the published paper, I noticed a substantial conceptual and structural overlap between the two studies that requires immediate clarification. Both studies utilize the exact same cross-sectional data to explore the interrelationships among Athlete Burnout, Academic Burnout, and Cognitive Reappraisal. In the published work, Athlete Burnout predicts Academic Burnout with Cognitive Reappraisal acting as a mediator. In the current manuscript, Athlete Burnout is positioned as a mediator, while Cognitive Reappraisal serves as a moderator. Reconfiguring the statistical roles of the same core variables from a single survey administration raises significant questions about the distinct theoretical contribution of this second paper. To satisfy the publication standards of PLOS ONE, I strongly encourage the authors to explicitly clarify the incremental value of this manuscript in the Introduction and Discussion sections, justifying why these variables were not integrated into a single, comprehensive model originally.

**Response:**Thank you for this professional and constructive suggestion. In the revised manuscript, we have explicitly clarified that the two studies used the same cross-sectional survey data from the same cohort of participants and included some overlapping variables. However, the two studies addressed different theoretical questions.

Specifically, our published article examined the burnout spillover process in which athlete burnout was associated with academic burnout through cognitive reappraisal and sleep quality. In contrast, the present study is grounded in Conservation of Resources theory and treats perceived social support as the predictor, psychological resilience and athlete burnout as sequential mediators, and cognitive reappraisal as a moderating emotion-regulation resource.

Therefore, the incremental contribution of the present study lies in shifting the research focus from the burnout spillover process to a resource-oriented explanation; that is, how perceived social support is associated with academic burnout through the sequential pathway of psychological resilience and athlete burnout, and how this process varies according to athletes’ levels of cognitive reappraisal.

We have also further explained why these variables were not integrated into one comprehensive model. The two studies were designed to answer different theoretical questions. Including perceived social support, psychological resilience, athlete burnout, cognitive reappraisal, sleep quality, and academic burnout in a single comprehensive model would substantially increase model complexity and weaken the theoretical focus of the specific mechanisms examined in each study. Therefore, we tested two theoretically distinct models with clear boundaries to maintain theoretical clarity and avoid overcomplicating the model. **Please refer to Lines 171–182 and 440–446 in the revised manuscript.**

**Q2.** Table 1 details important athletic characteristics such as "Sports items" (combat, ball, track) and "Sport level." Strikingly, these critical variables were completely excluded as covariates in the mediation models. Athlete burnout and psychological resilience are highly sensitive to the competitive level and the specific nature of the sport (e.g., individual versus team sports). Omitting these sport-specific covariates undermines the validity of the findings. The authors should rerun the analyses controlling for these athletic characteristics or thoroughly justify their exclusion.

**Response:**Thank you for this helpful suggestion. We reran the mediation model with sport type and competitive level included as additional covariates. The results showed that the main hypothesized paths remained significant, indicating that the indirect association between perceived social support and academic burnout through psychological resilience and athlete burnout was robust after controlling for these sport-specific characteristics.

We have clarified the covariates in the Methods section, updated the Results section accordingly, and noted in the relevant tables/figure notes that sport type and competitive level were controlled. **Please refer to Line 303 and Table 4 in the revised manuscript.**

Q3.The reliance on Harman’s single-factor test to address Common Method Bias (CMB) is insufficient by modern psychometric standards. Harman’s test is widely considered an insensitive diagnostic tool. Given that all data are cross-sectional self-reports, the authors must employ more robust methods to evaluate CMB, such as conducting a Confirmatory Factor Analysis (CFA) with an unmeasured latent method factor (ULMC) or using a theoretically unrelated marker variable.

**Response:**Thank you for this valuable comment. We agree with the reviewer that Harman’s single-factor test has limitations and should not be used as the sole evidence for assessing common method bias. In response to this comment, we supplemented the revised manuscript with CFA-based tests of common method bias, including comparisons with alternative models and an unmeasured latent method construct (ULMC) model.

The hypothesized five-factor CFA model showed better fit than the alternative models (χ² = 1127.001, df = 125, CFI = 0.952, TLI = 0.941, RMSEA = 0.065, SRMR = 0.050). After adding an unmeasured latent method factor with equal loadings on all observed indicators, the ULMC model showed no substantive improvement in fit (χ² = 1127.001, df = 124, CFI = 0.952, TLI = 0.940, RMSEA = 0.065). These results suggest that common method bias was unlikely to substantially affect the findings of this study.**Please refer to Line 317-325 in the revised manuscript and Supplementary S1 Table.**

Q4.The study focuses on Cognitive Reappraisal (CR) as the sole emotion regulation moderator. While theoretically sound, CR is strictly an intrapersonal (internal) emotion regulation strategy. Given that the primary predictor—Perceived Social Support—is an inherently interpersonal external resource, relying exclusively on an internal coping mechanism creates a conceptual gap. The authors should expand their discussion to acknowledge the limitation of only assessing intrapersonal regulation and discuss how interpersonal emotion regulation strategies might synergize with perceived social support to combat burnout. For context, the authors might refer to recent literature such as "The Association Between Autistic Traits and Depression in College Students: The Mediating Roles of Interpersonal Emotion Regulation and Social Self-Efficacy" to explore how interpersonal emotion regulation functions alongside social variables.

**Response:** Thank you for this professional and constructive suggestion. In response to the reviewer’s comment, we have added a discussion on how interpersonal emotion-regulation strategies may work together with perceived social support to address burnout. This revision has been incorporated into the Discussion section **(Lines 519–528).** We have also explicitly acknowledged this limitation in the Limitations section **(Lines 613–618).**

Q5. The authors utilized a single composite score for both Academic Burnout (AB) and Athlete Burnout (ATHB). However, burnout is universally recognized as a multidimensional syndrome (e.g., emotional exhaustion versus diminished accomplishment). Combining these distinct dimensions into a single construct may mask specific, nuanced mechanisms. Advanced mediation models often deconstruct the dependent variable to reveal dimension-specific pathways. The authors should justify their use of a total score, or ideally, conduct an exploratory path analysis examining the specific dimensions of burnout.

Response:Thank you for this important suggestion. We agree that both academic burnout and athlete burnout have multidimensional structures, and dimension-level analyses may reveal more nuanced mechanisms. However, the present study used total scores mainly because the proposed model focused on the overall levels of academic burnout and athlete burnout rather than dimension-specific pathways. This approach is also consistent with the scoring procedures of the two scales, and the total scores of both scales showed good internal consistency in the present sample.

We considered the possibility of conducting exploratory dimension-level path analyses. However, simultaneously separating academic burnout and athlete burnout into multiple dimensions would substantially increase the number of model parameters and multiple comparisons, which may go beyond the core focus of the original theoretical model. Therefore, we decided to retain the total-score analytic strategy and added an explanation for this decision in **Section 2.1 Instruments.**

Q6.Due to the cross-sectional nature of the data, the serial mediation model implies a directional flow that cannot be definitively confirmed. Causal language (e.g., “how PSS influences AB,” “PSS alleviates AB”) should be tempered carefully throughout the abstract and discussion, explicitly framing the results as associative rather than causal.

**Response:**Thank you for this helpful suggestion. In response to the reviewer’s comment, we revised the Abstract and Discussion sections to avoid causal wording and to present the findings as associations. Specifically, we replaced expressions such as “influence” and “alleviate” with more cautious terms, such as “was associated with” and “was indirectly associated with.”

Q7. The path diagrams (Figures 1, 2, and 3) appear somewhat rudimentary and visually disjointed. Redesigning these flowcharts for improved aesthetic clarity and ensuring they meet PLOS ONE’s high-resolution figure guidelines will significantly improve readability. Finally, the discussion on practical interventions remains somewhat generic; please elaborate on how athletic departments and coaches can tangibly implement the suggested emotion regulation training in a dual-career student-athlete context.

Response:Thank you for this valuable comment. In response to the reviewer’s suggestion, we have redrawn the three figures to improve their clarity and readability. We have also expanded the Discussion section to further elaborate the practical implications for college athletes in the dual-career context.

Specifically, **Lines 570–576** of the revised manuscript now indicate that, from a practical perspective, colleges, athletic departments, teachers, coaches, and counselors should combine CR training with problem-focused and context-focused support. These supports may include academic guidance, emotional support, ATHB monitoring, training-load adjustment, recovery management, instrumental academic support, and coordination of academic–athletic role demands.

These revisions make the practical recommendations more specific and better address the intervention needs of college athletes in the dual-career context.

**Response to Reviewer 2**

Dear Reviewer,

We sincerely thank you for your careful evaluation of our manuscript and for providing constructive and insightful comments. Your suggestions were highly valuable and helped us further improve the presentation of the study, the methodological description, and the interpretation of the findings. We greatly appreciate the time and effort you devoted to reviewing our work.

In response to your comments, we have carefully revised the manuscript and provided detailed point-by-point responses below. We believe that these revisions have further improved the clarity, rigor, and overall quality of the manuscript.

Sincerely,

The Authors

**Q1**. The authors acknowledge in Lines 206–209 that the dataset used in the present study was collected from the same cohort of college athletes as their previously published study (Reference 33: Xu et al., 2025, Frontiers in Psychology). The earlier study and the present manuscript appear to share several closely related elements: academic burnout serves as the dependent variable in both papers, athlete burnout is a key variable in both, and emotion regulation/cognitive reappraisal is involved in both analytical frameworks.

I do not suggest that the use of the same cohort across multiple studies is inherently inappropriate. However, when the same participant cohort and closely related psychological variables are used across separate manuscripts, the relationship between the studies should be disclosed with greater transparency. At present, the manuscript addresses this issue only briefly, stating that the research objectives, variable selection, and analytical framework are different. This statement is helpful, but it does not provide enough detail for readers to evaluate the degree of overlap and the distinct contribution of the present manuscript.

I recommend that the authors add a brief clarification in the Methods section, or in the supplementary materials if space is limited, explaining the relationship between the present study and the previously published paper. This clarification should specify which variables overlap across the two studies, whether the relevant measures were collected during the same survey administration, and what distinct theoretical or analytical contribution the present manuscript makes beyond the prior publication.

**Response:**Thank you for this professional and constructive suggestion. In the revised manuscript, we have explicitly clarified that the two studies used the same cross-sectional survey data from the same cohort of participants and included some overlapping variables. However, the two studies addressed different theoretical questions.

Specifically, our published article examined the burnout spillover process in which athlete burnout was associated with academic burnout through cognitive reappraisal and sleep quality. In contrast, the present study is grounded in Conservation of Resources theory and treats perceived social support as the predictor, psychological resilience and athlete burnout as sequential mediators, and cognitive reappraisal as a moderating emotion-regulation resource.

Therefore, the incremental contribution of the present study lies in shifting the research focus from the burnout spillover process to a resource-oriented explanation; that is, how perceived social support is associated with academic burnout through the sequential pathway of psychological resilience and athlete burnout, and how this process varies according to athletes’ levels of cognitive reappraisal.

We have also further explained why these variables were not integrated into one comprehensive model. The two studies were designed to answer different theoretical questions. Including perceived social support, psychological resilience, athlete burnout, cognitive reappraisal, sleep quality, and academic burnout in a single comprehensive model would substantially increase model complexity and weaken the theoretical focus of the specific mechanisms examined in each study. Therefore, we tested two theoretically distinct models with clear boundaries to maintain theoretical clarity and avoid overcomplicating the model. **Please refer to Lines 171–182 and 417–426 in the revised manuscript.**

**Q2.** Lines 258–268 describe the Sports Mental Toughness Questionnaire (SMTQ), including its origin, dimensional structure, scoring method, and internal reliability. However, in the correlation matrix and subsequent mediation and moderation models, the corresponding construct is labeled as psychological resilience (PR), rather than mental toughness. This creates conceptual and methodological ambiguity.

The authors should explicitly state whether the SMTQ total score was used as the operational indicator of PR. If so, this operational decision requires clear justification, because mental toughness and psychological resilience are related but not identical constructs. The Methods section should explain why a measure originally designed to assess sports mental toughness can validly represent psychological resilience in the present model. The Results section should also make the naming consistent, so that readers can clearly understand whether the construct being tested is mental toughness, psychological resilience, or a theoretically justified proxy for resilience.

At present, the manuscript appears to move from the measurement label “sports mental toughness” to the analytical label “psychological resilience” without sufficient explanation. This should be clarified before readers can properly evaluate the validity of the mediation model.

**Response:**Thank you for this important comment. We agree that the original manuscript did not clearly explain the transition between the measurement label “sport mental toughness” and the analytic label “psychological resilience (PR),” which may have caused conceptual and methodological ambiguity.

To clarify this issue, we have revised the Methods section to explicitly state that the total score of the SMTQ was used as an operational indicator of psychological resilience in the present study. We further clarified that psychological resilience in this manuscript should be understood as sport-specific psychological resilience, or resilience-related psychological resources in the sport context, rather than a direct measure of general psychological resilience. Specifically, the revised manuscript now states that all SMTQ item scores were summed to form a total score, and this total score was used to operationalize resilience-related psychological resources in the sport context, namely sport-specific PR.

In addition, we have revised the Results section, relevant tables, and model descriptions to improve the consistency of variable naming. The variable labeled as PR in the correlation matrix and subsequent mediation and moderation models refers to the operational indicator of sport-specific PR derived from the SMTQ total score, rather than a direct measure of general psychological resilience. **Please refer to Lines 275–282 in the revised manuscript.**

Q3.Related to the previous point, the manuscript repeatedly frames the relevant mediator as “psychological resilience,” citing literature that defines resilience as the capacity for recovery, adaptation, and sustained functioning under adversity. However, the actual measurement instrument appears to be the SMTQ, which was designed to assess sports mental toughness rather than resilience per se.
Although mental toughness and psychological resilience overlap in sport psychology, they should not be treated as fully interchangeable without theoretical or empirical justification. Resilience generally emphasizes recovery, adaptation, and positive functioning after adversity or stress exposure, whereas mental toughness more strongly emphasizes confidence, constancy, persistence, and perceived control under pressure. These conceptual differences matter because the manuscript interprets the mediator as an internal psychological resource that is built through perceived social support and then reduces athlete burnout and academic burnout.
I recognize that the data have already been collected and that it may not be feasible to re-administer an alternative resilience measure, such as the Connor-Davidson Resilience Scale, to the same 1,918 participants. Therefore, I do not require the authors to replace the measure. However, the authors should address this construct-validity issue in three specific ways.
First, in the Methods section, the authors should add a paragraph justifying the use of the SMTQ to operationalize psychological resilience. This justification should cite sport psychology literature showing the conceptual and empirical overlap between mental toughness and resilience, especially in athlete populations.
Second, in the Limitations section, the authors should explicitly acknowledge that the SMTQ was not originally designed to measure psychological resilience per se. The authors should explain that this operationalization choice may affect the interpretation of the PR-related findings and that future research using dedicated resilience measures would be necessary to verify the robustness of the current mediation results.
Third, the authors should provide additional evidence that the construct measured by the SMTQ is empirically distinguishable from the other constructs in the model. This is especially important given the relatively high correlations among some self-report variables, such as the correlation between PR and CR. A confirmatory factor analysis, or another appropriate test of discriminant validity, would help demonstrate that the SMTQ-based mediator captures a distinct construct rather than reflecting a broader general psychological adjustment factor.
Without these clarifications, the current manuscript risks overstating the evidence for a psychological resilience mechanism when the empirical measure may more accurately reflect sports mental toughness. This does not necessarily invalidate the findings, but it does require a more cautious and transparent interpretation.

**Response:**Thank you for this detailed and constructive suggestion. In response to the reviewer’s comment, we made revisions in the following three aspects.

First, in the Methods section, we added a clarification regarding the use of the SMTQ total score as an operational indicator of sport-specific PR. The revised manuscript now states that although the SMTQ was originally developed to measure sport mental toughness rather than psychological resilience in a general sense, the sport psychology literature suggests that mental toughness and psychological resilience share a closely related conceptual space, as both involve adaptive functioning under stressful conditions and the capacity to recover from adversity. In addition, previous athlete studies and systematic reviews have used or identified the SMTQ as a resilience-related measurement tool in competitive sport contexts. Therefore, in the present athlete sample, using the SMTQ total score as an operational indicator of sport-specific PR has a theoretical basis. **Please refer to Lines 275–282 in the revised manuscript.**

Second, in the Limitations section, we have explicitly acknowledged that the SMTQ was not originally designed to measure general psychological resilience. Therefore, using the SMTQ total score to represent PR may affect the interpretive boundaries of PR-related findings. We further noted that future studies should use dedicated psychological resilience measures, such as the Connor-Davidson Resilience Scale, or compare different measures of psychological resilience and sport mental toughness to verify the robustness of the current mediation results. **Please refer to Lines 602–607 in the revised manuscript.**

Third, we agree that it is necessary to further examine the empirical distinction between the psychological resilience construct operationalized by the SMTQ and other constructs in the model, especially cognitive reappraisal. To this end, we added CFA model comparisons and discriminant validity tests in the Results section. The results showed that the hypothesized five-factor CFA model, including perceived social support, psychological resilience, athlete burnout, cognitive reappraisal and academic burnout had an acceptable fit and performed better than the alternative models: χ² = 1127.001, df = 125, CFI = 0.952, TLI = 0.941, RMSEA = 0.065, SRMR = 0.050. Further discriminant validity tests showed that the AVE values of all constructs were above 0.50, and the square root of each construct’s AVE was greater than its correlations with other constructs. In addition, the HTMT values ranged from 0.379 to 0.727, all below the recommended threshold of 0.85. These results suggest that, although psychological resilience was correlated with variables such as cognitive reappraisal , the construct measured by the SMTQ could still be empirically distinguished from cognitive reappraisal and other constructs, and was unlikely to reflect a broader general psychological adaptation factor. **Please refer to Lines 343–349 in the revised manuscript and Tables S1–S3.**

Q4. The study employs five self-report instruments simultaneously, with notably high inter-correlations among several variables. For example, Table 2 reports relatively high correlations between PSS and CR (r = 0.658) and between PR and CR (r = 0.560). Under these circumstances, it would be important to provide CFA results, or other appropriate evidence of discriminant validity, to confirm that the five constructs are empirically distinguishable.

At present, the manuscript reports internal consistency coefficients and bivariate correlations, but it does not provide sufficient evidence that the measured constructs are distinct from one another. The particularly high correlation between PSS and CR raises the question of whether these two variables may partly reflect overlapping aspects of a broader positive psychological adjustment factor. I recommend that the authors report additional evidence of construct distinctiveness, such as confirmatory factor analysis, comparison of alternative factor models, AVE-based discriminant validity, HTMT, or another appropriate approach.

**Response:**Thank you for this helpful suggestion. In response to the reviewer’s comment, we supplemented the revised manuscript with CFA model comparisons, AVE-based discriminant validity tests, and HTMT analyses.

The results showed that the five-factor CFA model, including perceived social support, athlete burnout, psychological resilience, cognitive reappraisal, and academic burnout as five latent variables, had an acceptable fit and performed better than the alternative models (χ² = 1127.001, df = 125, CFI = 0.952, TLI = 0.941, RMSEA = 0.065, SRMR = 0.050). Further discriminant validity analyses showed that the AVE values of all constructs were above 0.50, and the square root of each construct’s AVE was greater than its correlations with other constructs. In addition, the HTMT values ranged from 0.379 to 0.727, all below the recommended threshold of 0.85. Notably, the HTMT values for PSS–CR and PR–CR were 0.727 and 0.689, respectively, indicating acceptable discriminant validity even between relatively highly correlated constructs.

**These revisions can be found in Sections 3.1 and 3.2 of the revised manuscript, and the full results are reported in Supplementary Tables S1–S3.**

**Q5.** Table 3 reports the 95% confidence interval for the PSS → ATHB → AB indirect effect as [−0.065, −0.028], whereas Line 312 in the text reports the same result as [−0.064, −0.027]. Both the lower and upper bounds differ. Although the discrepancy is numerically small, reporting different values for the same result within the same manuscript undermines the credibility of the data. The authors should verify the original PROCESS output and ensure consistency throughout.

**Response:**Thank you for pointing this out. We rechecked the original PROCESS output and carefully reviewed all indirect effects and their bootstrap confidence intervals reported in the text and Table 3. The previous inconsistency was mainly due to rounding and an oversight during the reporting process.

In the revised manuscript, we have standardized and corrected the relevant values throughout the text. Specifically, the indirect effect of PSS on AB through ATHB is now consistently reported in both the main text and Table 3 as Effect = −0.045, 95% CI [−0.065, −0.027]. We also checked the other indirect effects, the total indirect effect, and the total effect to ensure consistency among the main text, tables, and the PROCESS output.

Q6. Figure 2 presents standardized path coefficients from the serial mediation model (Model 6), such as PR → AB = −0.150 and ATHB → AB = 0.327. These values differ from those in Table 4, which reports Model 89 coefficients (−0.108 and 0.302, respectively). I understand this discrepancy arises because the two models include different sets of predictors, which is expected. However, the authors never provide the full regression coefficient table for Model 6. Only the indirect effects appear in Table 3, leaving the path coefficients shown in Figure 2 unverifiable against any tabulated data. I recommend adding a complete Model 6 regression table in the main text or supplementary materials.

**Response:**Thank you for this helpful suggestion. In response to this comment, we further included sport item and sport level as additional covariates on the basis of the original control variables and re-estimated PROCESS Model 6. In the revised manuscript, we have added S4 **Table** to the Supplementary Materials, which reports the full regression results of Model 6, including regression coefficients, standard errors, p values, 95% confidence intervals, control variables, and model statistics. This allows the main path coefficients presented in Figure 2 to be directly verified.

In addition, Figure 2 presents the path coefficients of the serial mediation model estimated using PROCESS Model 6, whereas Table 4 presents the coefficients of the moderated serial mediation model estimated using PROCESS Model 89. Because Model 89 additionally included cognitive reappraisal and the interaction terms PSS × CR, PR × CR, and ATHB × CR in the equation predicting academic burnout, the path coefficients for PR → AB and ATHB → AB were not expected to be identical to the corresponding coefficients in Model 6. Thus, the observed differences reflect the different sets of predictors included in the two model specifications, rather than inconsistencies in the statistical results.

**Please refer to S4 Table in the Supplementary Materials.**

**Q7.** Table 4 reports the p-value for the PSS × CR interaction term as p = 0.010, while Line 327 describes the same result as “p < 0.05.” The manuscript alternates between exact p-values and inequality notation throughout. I recommend adopting a uniform reporting standard.

**Response:**Thank you for pointing this out. After further controlling for sport item and sport level in addition to the original covariates, we reran PROCESS Model 89. The updated results showed that the interaction term PSS × CR significantly predicted AB (β = −0.052, p = 0.009). Therefore, we have revised this result consistently in **Table 4 and the Results section** as p = 0.009, instead of using general expressions such as “p < 0.05” or “p < 0.01.” We have also standardized the reporting format of other p values throughout the manuscript.

Q8. When the correlation between an independent variable and a moderator reaches 0.66, interaction term estimates may become unstable. The authors note in Lines 283–284 that variables were mean-centered prior to computing interaction terms, which is appropriate. However, mean-centering only reduces collinearity between the interaction term and the constituent main effects; it does not address collinearity between the main effects themselves. I recommend reporting VIF (Variance Inflation Factor) values for each predictor across all models.

**Response:** Thank you for this helpful comment. We conducted collinearity diagnostics for the predictors and all covariates included in the regression models. The results showed that the VIF values of all predictors ranged from 1.010 to 2.014, which were all below the commonly used threshold of 5. The Tolerance values ranged from 0.496 to 0.990, which were all above 0.10. These results indicate that multicollinearity was unlikely to seriously affect the model estimates.

We have added these results to **Section 3.2, “Correlation Analysis and Discriminant Validity Assessment,”** in the revised manuscript.

**Q9.** Lines 287–294 report that the first unrotated factor explained 23.769% of the variance, below the 40% threshold. However, Harman’s test has been widely criticized in the methodological literature for its low sensitivity and limited statistical power. I recommend that the authors at minimum acknowledge this limitation, and ideally supplement the test with a more robust approach, such as adding a common method factor within the CFA framework.

**Response:**Thank you for this important suggestion. We agree that Harman’s single-factor test has relatively low sensitivity and should not be used as the sole basis for assessing common method bias. Therefore, **in Section 3.1, “Common Method Bias,”** of the revised manuscript, we retained Harman’s test as a preliminary diagnostic and further supplemented it with a CFA-based unmeasured latent method construct (ULMC) analysis.

Specifically, we first established a five-factor CFA model. We then added an unmeasured latent method construct to the five-factor CFA model to examine the potential influence of common method bias. The results showed that the five-factor CFA model had an acceptable fit (χ² = 1127.001, df = 125, CFI = 0.952, TLI = 0.941, RMSEA = 0.065). Subsequently, we added an unmeasured latent method construct to this model and constrained its loadings on all observed indicators to be equal. The results showed that the ULMC model did not lead to a substantial improvement in model fit (χ² = 1127.001, df = 124, CFI = 0.952, TLI = 0.940, RMSEA = 0.065, SRMR = 0.050). These results suggest that common method bias was unlikely to substantially affect the main findings of this study.

**Q10.** The moderated serial mediation model tested in the manuscript specifies CR as a moderator of paths leading to AB, including the PSS → AB, PR → AB, and ATHB → AB paths. This specification is theoretically plausible, but the manuscript does not sufficiently justify why CR was modeled only at these later-stage paths rather than at earlier stages of the serial chain, such as PSS → PR, PSS → ATHB, or PR → ATHB.

Given that the Introduction describes CR as a factor that may influence the transformation of external support into internal psychological resources, one might also expect CR to moderate earlier resource-transformation paths. I recommend that the authors provide a clearer theoretical rationale for the chosen PROCESS Model 89 specification and acknowledge that alternative moderated mediation models could test different boundary conditions within the serial pathway.

**Response:**Thank you for this constructive comment. We agree that the original wording in the Introduction may have led readers to infer that CR could also moderate earlier resource-transformation pathways. To avoid this ambiguity, we revised the Introduction to further clarify the theoretical rationale for the specification of PROCESS Model 89.

Specifically, drawing on the process model of emotion regulation and COR theory, we now conceptualize CR as an outcome-proximal emotion-regulation resource in relation to AB. Because CR primarily involves the reinterpretation of stressful situations and emotional experiences, it is more directly relevant to how college athletes appraise perceived support, internal psychological resources, and burnout-related experiences in relation to academic burnout. Therefore, in the present study, CR was specified as a moderator of the direct paths leading to AB, namely PSS → AB, PR → AB, and ATHB → AB, rather than as a moderator of earlier resource-accumulation paths such as PSS → PR, PSS → ATHB, or PR → ATHB.

We have also added a note in the Discussion/Limitations section acknowledging that alternative moderated mediation models may also be theoretically plausible. Future longitudinal studies should compare models with different moderator positions within the sequential pathway.

**Please refer to Lines 160–164 and the Limitations section in the revised manuscript.**

**Q11.** Table 4 and Figure 5 show that CR positively moderates the ATHB → AB path (β = 0.078, p < 0.001). The simple slope analysis indicates that the association between ATHB and AB is stronger among athletes with higher CR (β = 0.380) than among those with lower CR (β = 0.223). In other words, higher CR is associated with a stronger ATHB–AB link, rather than a weaker one.

This finding complicates, and partly contradicts, the manuscript’s general framing of CR as a uniformly protective factor. Although CR appears to strengthen the protective association between PSS and AB, its moderating role in the ATHB → AB path operates in the opposite direction from what a simple protective-factor interpretation would predict.

The authors briefly touch on this result in Lines 476–479 but do not meaningfully engage with its theoretical implications. I believe this finding warrants a more thorough discussion. Possible explanations might include: (a) cognitive reappraisal may be effective under mild stress but may fail or even backfire when athletic burnout reaches severe levels, with high-CR individuals becoming more acutely aware of their deteriorating state; (b) high-CR individuals may over-rely on reappraisal strategies while neglecting problem-focused coping, leading to strategy exhaustion when burnout accumulates; or (c) there may be blurred boundaries between reappraisal and rumination, such that some individuals’ “reappraisal” effectively constitutes repeated cognitive processing of negative experiences. These possibilities deserve serious discussion rather than a cursory mention.

In its current form, the Discussion risks overstating the protective role of CR and underinterpreting a theoretically important, counterintuitive moderation effect.

**Response:**Thank you for this important comment. We agree that the positive moderating effect of CR on the ATHB → AB path complicates the interpretation of CR as a uniformly protective factor. Specifically, the revised Results section now explicitly states that the ATHB × CR interaction term was significant and positive (β = 0.078, p < 0.001), indicating that CR strengthened the ATHB → AB path. The simple slope analysis showed that ATHB had a stronger predictive effect on AB at higher levels of CR (β = 0.381, p < 0.001), whereas this effect was relatively weaker at lower levels of CR (β = 0.224, p < 0.001).

In the revised Discussion section, we have explicitly acknowledged that the simple slope analysis showed a stronger ATHB–AB association among individuals with higher CR. Accordingly, we removed statements that implied that CR weakened the ATHB–AB association and revised the interpretation to emphasize the path-specific and context-dependent role of CR. Specifically, we added several possible explanations for this counterintuitive finding. First, CR may be adaptive under mild or moderate stress, but when burnout-related strain becomes severe, repeated cognitive reframing may become less effective or even cognitively costly. Second, individuals with higher CR may over-rely on cognitive reframing while engaging less in problem-focused coping strategies, which may contribute to strategy exhaustion when ATHB continues to accumulate. Third, under chronic burnout conditions, the boundary between adaptive reappraisal and rumination may become blurred; for some athletes, self-reported cognitive reappraisal may partly reflect repeated cognitive processing of burnout-related difficulties, which may help explain the stronger ATHB–AB association at higher levels of CR.

**Please refer to Lines 531–554 in the revised manuscript.**

Q12. The simple slope analysis for the PSS × CR interaction suggests that, among athletes with lower CR, the protective association between PSS and AB is weaker and does not reach conventional statistical significance (effect = −0.058, p = 0.066). By contrast, the association is stronger and statistically significant among athletes with higher CR (effect = −0.162, p < 0.001).

This pattern has important implications for intervention design. It suggests that enhancing social support alone may be less effective for athletes who have lower levels of cognitive reappraisal, and that social support interventions may need to be combined with emotion regulation training to produce more consistent benefits for reducing academic burnout.
However, the manuscript’s discussion of this point is too brief. I recommend that the authors elaborate on the practical implications of this conditional effect, especially with regard to how universities, coaches, counselors, and academic support staff might design interventions that integrate social support enhancement with emotion regulation training.

**Response：**Thank you for this helpful suggestion. We agree that the conditional effect of the PSS × CR interaction has important implications for intervention design. In the revised analysis, the simple slope estimates changed slightly, but the substantive interpretation remained consistent. Specifically, at higher levels of CR, PSS predicted lower AB (M + 1 SD; effect = −0.162, p < 0.001), whereas at lower levels of CR, this association was weaker and did not reach statistical significance (M − 1 SD; effect = −0.057, p = 0.072). **Please refer to Lines 375–378 in the revised manuscript.**

In response to the reviewer’s comment, we expanded the Discussion section to clarify the practical implications of this conditional effect. We now further explain that, for athletes with lower CR, simply increasing social support may be insufficient because they may have more difficulty cognitively reframing perceived support as a positive resource for coping with academic stress. Therefore, interventions targeting AB among college athletes should combine social support enhancement with CR training. **Please refer to Lines 570–579 in the revised manuscript.**

**Q13.** The AB equation in Table 4 explains 25.9% of the variance in academic burnout. While this represents a meaningful proportion of explained variance in psychological research, the manuscript does not report individual predictor effect sizes or discuss the practical magnitude of the observed associations. I recommend that the authors supplement the statistical significance tests with effect size indicators where appropriate, such as semi-partial effects, standardized effect interpretation, or f² values. More importantly, the Discussion should clarify what the observed effects mean for practical intervention design. For example, the authors should explain whether the effects of PSS, ATHB, and CR are sufficiently large to support targeted intervention programs, or whether the findings should be interpreted as preliminary evidence requiring further validation.

**Response:** Thank you for this constructive suggestion. In the revised Results section, we have supplemented the manuscript with effect size indices. According to the updated analysis, the academic burnout equation explained 26.2% of the variance, R² = 0.262, F(13, 1904) = 51.862, p < .001, corresponding to Cohen’s f² = 0.355, which indicates a relatively strong overall explanatory effect. **Please refer to Lines 371–374 in the revised manuscript.**

In addition, we further calculated the local effect sizes (local f²) of the core predictors in the academic burnout equation to reflect the unique contribution of each predictor after controlling for the other variables. The results showed that athlete burnout had the largest local effect size (local f² = 0.092), whereas the local effect sizes of perceived social support, psychological resilience, cognitive reappraisal, and the interaction terms were relatively small, ranging from 0.000 to 0.011. These effect size indices have been added **to Table 4.**

We also further clarified the practical implications of these effects in the Discussion section. The revised manuscript now indicates that ATHB had a relatively stronger unique association with AB and therefore may be a priority intervention target for reducing academic burnout among college athletes. In contrast, the smaller local effects of PSS, PR, CR, and the interaction terms suggest that social support enhancement, resilience-related psychological resource building, and CR training may be more appropriate as complementary components of a multicomponent intervention, rather than as stand-alone solutions.

**Q14.** The authors successively draw upon the stress-buffering model (Line 91), Conservation of Resources theory (Line 106), the process model of emotion regulation (Line 151), and later in the Discussion introduce the Job Demands–Resources model (Line 448) and self-regulation theory (Line 470). While each framework has some relevance, the current presentation lacks a unifying logical thread and reads more like a list of theories than an integrated framework. I recommend anchoring the paper in COR theory as the primary framework and clearly positioning the other theories in supporting roles.

**Response:**Thank you for this insightful suggestion. In response to the reviewer’s comment, we revised the Introduction and Discussion sections to position Conservation of Resources (COR) theory as the core theoretical framework of the present study, while treating other theories only as supplementary perspectives.

Specifically, COR theory is now used as the overarching framework to explain the processes of resource gain, resource protection, and resource loss among college athletes facing dual academic–athletic demands . The stress-buffering model is now used only as a supplementary perspective to explain the role of social support as an external resource, whereas the Process Model of Emotion Regulation is used to support CR as a boundary condition that may influence how athletes interpret and use available resources. We have also removed references to the Job Demands–Resources model and self-regulation theory as independent explanatory frameworks in the Discussion section to avoid theoretical fragmentation**(Lines 165–182).**

Q15. The proposed sequence assumes that social support builds resilience, which in turn reduces athletic burnout, and the reduction in athletic burnout then lowers academic burnout. However, under a cross-sectional design, alternative causal orderings are equally plausible. For example, ATHB → PR → AB (athletic burnout erodes resilience, which then exacerbates academic burnout) is theoretically defensible. I recommend that the authors provide more thorough theoretical justification for the chosen ordering in the Introduction, and indicate whether any competing models were tested. If not, this should be acknowledged in the Limitations section.

**Response:**Thank you for this important comment. We agree that, given the cross-sectional design, the proposed sequential pathway cannot be interpreted as a strict causal order, and other directional pathways are also theoretically possible. Therefore, we have further softened the causal wording throughout the revised manuscript and added a clearer theoretical rationale in the Introduction for specifying the PSS → PR → ATHB → AB sequence.

Specifically, perceived social support was placed at the beginning of the model because social support is generally regarded as a relatively external psychosocial resource that can provide emotional, informational, and instrumental support, thereby promoting adaptive psychological resources under stressful conditions. PR was positioned before athlete burnout because it reflects individuals’ adaptive and regulatory capacity in response to stress and challenges, which may theoretically buffer the impact of training, competition, and academic demands on burnout experiences. ATHB was placed before AB based on the dual academic–athletic role context of college athletes: persistent exhaustion in the athletic domain may occupy psychological and behavioral resources and become associated with burnout in the academic domain. Accordingly, the present study examined PSS → PR → ATHB → AB as a theory-driven sequential pathway based on Conservation of Resources theory and relevant prior evidence.

At the same time, we have explicitly acknowledged in the Limitations section that this study did not systematically test competing sequential models. Because the study used a cross-sectional self-report questionnaire design, it cannot determine the temporal order or causal direction among the variables, nor can it rule out alternative pathways, such as ATHB → PR → AB. Future research should use longitudinal, cross-lagged, or experimental/intervention designs to further compare the plausibility and robustness of different competing pathways.

**Please refer to the revised Introduction and Limitations sections.**

Q16. H7 reads “CR moderates the relationship between ABTH and AB.” The abbreviation should be “ATHB,” not “ABTH.” Please also check the x-axis label of Figure 5 and all related figure labels for abbreviation consistency, and correct this issue throughout the manuscript.

**Response:** Thank you for pointing out this inconsistency in the abbreviation. We have corrected “ABTH” to “ATHB” in the statement of H7. We also carefully checked the x-axis label, title, and note of Figure 5, as well as the table labels and all related descriptions throughout the manuscript. In the revised manuscript, “ATHB” is now used consistently to refer to athlete burnout.

**Q17.** Lines 386–389 discuss how PSS may modulate the sympathetic nervous system and the HPA axis to reduce physiological stress responses. However, the present study collected no physiological data whatsoever. While citing relevant literature as theoretical background is acceptable, the current phrasing risks misleading readers into thinking these mechanisms are findings of the present study. I recommend adjusting the wording to clearly mark these as speculative interpretations based on prior literature.

**Response:**Thank you for this important comment. To avoid potential misunderstanding, we have removed the sentences discussing the regulation of the sympathetic nervous system and the HPA axis by PSS. The revised Discussion is mainly grounded in COR theory and interprets PSS as an external social resource, focusing on its association with lower AB. This revision avoids overinterpretation and ensures that the discussion remains consistent with the variables actually measured in the present study.

**Q18.** The Limitations discussion (Lines 496-509) covers cross-sectional design, self-report instruments, and the non-significant PR × CR interaction, which is reasonable. However, it omits several points worth discussing: (a) the sample was recruited through convenience sampling from Chinese university sports teams, and the representativeness of the sample may be limited, particularly given the uneven distribution of sport types and athlete levels; (b) using the SMTQ to operationalize psychological resilience may introduce construct validity concerns (see Q3); and (c) the high collinearity between PSS and CR (r = 0.658) may affect the stability of interaction term estimates (see Q8).

Response:Thank you for this helpful suggestion. We have revised this section accordingly.

First, we now acknowledge that the participants in this study were recruited through convenience sampling from Chinese university sports teams, which may limit the generalizability of the findings. Therefore, future research should use more diverse and representative samples and examine whether the model remains stable across different sports, competitive levels, and cultural contexts.

Second, we have added a limitation regarding the use of the SMTQ to operationalize PR. Although the SMTQ can reflect resilience-related characteristics in the sport context, it was originally developed to measure sport mental toughness rather than general psychological resilience. Therefore, using the SMTQ total score as an operational indicator of PR may raise certain construct-validity concerns and affect the interpretive boundaries of PR-related findings. We now suggest that future studies should use dedicated psychological resilience measures or compare multiple measurement tools to further verify the robustness of the findings.

Third, we have expanded the discussion of collinearity between PSS and CR. We agree that the relatively high correlation between PSS and CR may affect the stability of the interaction-term estimates. In response to Q8, we conducted additional collinearity diagnostics. The results showed that the VIF values of all predictors ranged from 1.286 to 1.745, and the tolerance values ranged from 0.573 to 0.777, suggesting that severe multicollinearity was unlikely. Nevertheless, we still acknowledge in the Limitations section that the relatively high PSS–CR correlation should be considered when interpreting the interaction effects, and that future studies should further replicate these moderation effects in independent samples.

**Please refer to the Limitations section in the revised manuscript.**

Q19. The reference list uses journal abbreviations for some entries and full journal names for others; some entries include complete page information while others provide only article numbers; and the formatting of translated Chinese-language references is inconsistent with other entries. Please revise in accordance with PLOS ONE reference formatting requirements.

Response:Thank you for this helpful comment. We have carefully revised the reference list according to the PLOS ONE reference formatting requirements. Journal title abbreviations, page ranges or article numbers, DOI information, and translated Chinese-language references have been standardized throughout the reference list. We also checked the numbering of all references to ensure consistency with the in-text citations.

**Q20.** After a careful reading of the Introduction, I find that the empirical grounding for the hypotheses is uneven. H1 and H2 are reasonably well supported by cited literature. H3, however, relies on the ATHB → AB spillover assumption, for which the only cited evidence is the authors’ own prior work (Reference 33, Line 142: “our preliminary work suggests”). A single self-citation is insufficient to anchor a mediation hypothesis; the authors should supplement this with independent evidence on cross-domain burnout transfer.

H4, the core serial mediation hypothesis, lacks direct empirical precedent. No cited study has tested a sequential PSS → PR → ATHB → AB pathway, and the critical PR → ATHB link receives only a brief mention at Lines 136–138 (Reference 29), citing a correlational association rather than a mediation relationship. A four-variable serial mediation hypothesis requires stronger literature support than what is currently provided.

Most critically, the moderation hypotheses H5 through H8 (Lines 176–179) suffer from a systematic logical gap. The authors’ argument in Lines 154–161 proceeds as follows: CR is related to AB (Reference 36), CR is related to resilience (Reference 37), and CR is related to ATHB (Reference 38). From these bivariate associations, the authors derive hypotheses that CR moderates specific paths (PSS → AB, PR → AB, ATHB → AB, and the serial indirect effect). However, "CR correlates with variable X" does not logically entail "CR moderates the path from X to Y." Correlation and moderation are distinct statistical and conceptual relationships. Furthermore, Reference 36 actually examines CR as a mediator between social support and academic burnout, not as a moderator. Using a mediation finding to justify a moderation hypothesis conflates two different theoretical mechanisms. I recommend that the authors either locate prior studies that have specifically tested CR (or related emotion regulation strategies) as a moderator of burnout pathways, or reframe the moderation hypotheses as more exploratory in nature and adjust the language in the Introduction accordingly.

**Response:**Thank you for this important and constructive comment. We agree that the original Introduction did not provide sufficiently balanced empirical support for H3, H4, and H5–H8. Accordingly, we have systematically revised the Introduction.

First, to strengthen the theoretical and empirical basis for H3, we added independent evidence regarding the relationship between burnout in the sport domain and burnout in the school/academic domain. Specifically, the revised manuscript cites research on the dual career of student-athletes, showing a strong positive correlation between school burnout and sport burnout. This suggests that burnout experiences in the academic and athletic domains are not independent of each other among student-athletes. Meanwhile, we now use previous evidence showing a significant association between ATHB and AB among college athletes as supplementary evidence, rather than as the sole basis for H3. Together with findings showing that higher perceived social support is associated with lower levels of ATHB, these studies provide stronger support for conceptualizing ATHB as a potential mediator between PSS and AB.

Second, regarding H4, we now explicitly acknowledge in the revised manuscript that, to the best of our knowledge, no prior study has directly examined the complete PSS → PR → ATHB → AB sequential pathway. Therefore, we have reframed H4 as a theory-driven sequential pathway based on COR theory and evidence from interconnected paths, rather than as a direct extension of an existing four-variable model. We added evidence showing that psychological resilience is associated with lower levels of ATHB, as well as athlete-related research that conceptualizes mental toughness as a resilience-related construct in the sport context and shows that it can mediate the relationship between social support and athlete burnout. Taken together, these findings provide empirical support for integrating the interconnected pathways among PSS, PR, ATHB, and AB. Based on this, the revised manuscript presents H4 as a theory-driven sequential pathway examining the potential links among external social resources, internal psychological resources, athletic-domain exhaustion, and academic-domain exhaustion under the dual academic–athletic demands faced by college athletes.

Third, we rewrote the theoretical rationale for H5–H8 to avoid conflating correlational, mediating, and moderating mechanisms. In the revised manuscript, we removed statements that directly inferred moderation hypotheses from bivariate associations between CR and AB, PR, or ATHB. Instead, we now justify the moderation hypotheses based on the Process Model of Emotion Regulation and empirical studies in which CR or related emotion-regulation strategies functioned as moderators in burnout-related pathways. For example, previous studies have shown that CR can moderate the association between intrusive rumination and academic burnout, as well as the relationships between stress and job burnout and between depression and job burnout. At the same time, we now explicitly acknowledge that direct evidence on whether CR moderates the specific pathways among PSS, PR, ATHB, and AB in college athletes remains limited. Therefore, CR is now presented as a theory-driven exploratory boundary condition in the revised manuscript.

Finally, in response to the reviewer’s question about why CR was placed on the later-stage paths, we further clarified that CR was conceptualized as a proximal emotion-regulation resource that mainly concerns how athletes reinterpret stressful situations, emotional experiences, and burnout-related appraisals. Therefore, the revised manuscript now explicitly states that the present study focused on whether CR moderated the direct paths leading to AB, namely PSS → AB, PR → AB, and ATHB → AB.

**Please refer to Lines 130–146 and 153–164 in the revised manuscript.**
