# Supplementary material for: The impact of perceived social support on college athletes’ academic burnout: The moderating role of cognitive reappraisal within a serial mediation model
Source: PLoS One. 2026 Oct 22;21(10):e0361222. doi: 10.1371/journal.pone.0361222 (PMC13690224; doi:10.1371/journal.pone.0361222)
Supplement: Supplementary file 8 [file pone.0361222.s008.docx]

Dear Editor，

Thank you for reviewing our manuscript. At the same time, give us the opportunity to make modifications. This revision has provided point-to-point responses to the specific questions raised by the reviewers. Thank you very much for your work. If you feel that there are any further modifications needed, please feel free to point them out.

Dear reviewer,

Thank you for carefully reviewing our manuscript and providing many helpful suggestions. We have made corresponding modifications to the manuscript based on your suggestions. We believe that with your help, the quality of our manuscript has been greatly improved. We hope that this modification can meet your requirements. If there are any shortcomings, please feel free to raise them again.

Once again, I would like to express my sincere gratitude for your patience and meticulousness.

Major Comments

**Major Comment 1:** The authors have responded constructively to my previous concerns about the transition from the measurement label “sport mental toughness” to the analytical label “psychological resilience.” In the revised Methods section, around Lines 275–282, the authors now state explicitly that the SMTQ total score was used to operationalize a sport-specific resilience-related psychological resource rather than general psychological resilience. The Limitations section also acknowledges that the SMTQ was not originally designed as a dedicated measure of psychological resilience. These additions improve the transparency of the manuscript.

Nevertheless, the underlying construct-validity issue remains only partially resolved. Sport mental toughness and psychological resilience occupy related conceptual territory, but they are not fully interchangeable. The SMTQ assesses confidence, constancy, and control under pressure, whereas resilience more specifically concerns adaptation, recovery, and sustained functioning following adversity. The newly added CFA, AVE, and HTMT analyses can demonstrate that the SMTQ-based factor is empirically distinguishable from PSS, CR, ATHB, and AB, but they cannot establish that the SMTQ is a direct measure of psychological resilience. Discriminant validity from other variables is not equivalent to content or convergent validity as resilience. The manuscript should therefore avoid presenting the mediator unqualifiedly as psychological resilience. The authors may either relabel the construct as “sport mental toughness” or consistently describe it as an “SMTQ-based resilience-related psychological resource in the sport context,” but the terminology and interpretation must be aligned throughout the Introduction, hypotheses, figures, tables, Results, Discussion, and Conclusion. Claims that the study directly demonstrates a psychological-resilience mechanism should be narrowed accordingly.

A related scoring issue also requires clarification. The original manuscript stated that SMTQ Items 2, 4, 7, 8, 9, and 10 were reverse-coded, whereas the revised description appears to omit Item 10. The original scoring description is located in the SMTQ subsection, Lines 258–268. The authors should verify the scoring key actually used to compute the analytic variable and state clearly which description is correct. If the dataset was scored correctly and the inconsistency is only textual, an explicit confirmation and correction would be sufficient. If the scoring computation itself differed, the descriptive statistics, reliability analysis, CFA results, correlations, and PROCESS models involving this mediator would need to be recalculated.

**Response:** Thank you for this important and constructive comment. We agree that sport mental toughness and psychological resilience are conceptually related but distinct constructs and that the CFA, AVE, and HTMT analyses cannot establish the SMTQ as a direct measure of psychological resilience. We also appreciate the reviewer’s identification of the related inconsistency in the reported SMTQ reverse-scoring key. We address the construct-validity and scoring issues separately below.

A. Construct clarification and terminology alignment

Following the reviewer’s recommendation, we have relabeled the mediator from psychological resilience (PR) to mental toughness (MT) throughout the manuscript and revised its conceptualization, measurement description, reporting, and interpretation accordingly.

(1) Because the SMTQ was originally developed to assess mental toughness rather than psychological resilience, the revised Introduction now conceptualizes MT as a psychological advantage manifested in the sport context, enabling athletes to maintain determination, focus, confidence, and control when coping with the demands of competition, training, and the athletic lifestyle. We also reconstructed the theoretical rationale using MT-related evidence concerning PSS, AB, ATHB, and school-adjustment outcomes. Accordingly, all hypotheses previously involving PR have been revised to refer to MT. These include the individual mediation pathway through MT, the sequential mediation pathway through MT and ATHB, the moderation of the MT–AB association by CR, and the moderated indirect pathway involving MT and ATHB. The corresponding hypotheses, figures, and model descriptions have been revised consistently, and no hypothesis now refers to a psychological-resilience mechanism.

Revisions: Introduction (Lines 109–128, Lines 143-144, Lines 193-199), Figure 1.

(2) Revisions to the Methods. The Methods section now states that the SMTQ was used to assess college athletes’ MT in coping with competitive stress and performance-related challenges and identifies confidence, constancy, and control as its three dimensions. The previous paragraph justifying the SMTQ as a psychological resilience-related measure has been removed.

Revisions: Methods, Section 2.1.5 (Lines 290-299).

(3) Revisions to the Results, figures, and tables. The terms “psychological resilience” and “PR” have been replaced with “mental toughness” and “MT” in the Results, figures, and tables. The CFA, AVE, and HTMT findings are now interpreted only as evidence that MT is empirically distinguishable from PSS, CR, ATHB, and AB, rather than as evidence that the SMTQ has content or convergent validity as a measure of psychological resilience.

Revisions: Results, Sections 3.2-3.4; Figures 2-3; Tables 2-4; and Supplementary Tables S1-S5.

Revisions to the Discussion, Conclusion, and Limitations. In Sections 4.2 and 4.4, the mediation and sequential mediation findings are now interpreted as indirect associations involving MT, rather than as evidence of a psychological-resilience mechanism. Where research on resilience is retained in Section 4.2, it is used only as complementary support for a broader resource-based perspective, with resilience and MT explicitly treated as distinct constructs. In Section 4.6, because the mediator is no longer conceptualized as psychological resilience, we removed the previous limitation concerning the use of the SMTQ instead of a dedicated psychological-resilience measure. The Conclusion has also been revised by replacing PR with MT and aligning the summary and implications with the revised construct interpretation.

Revisions: Discussion, Sections 4.2 and 4.4 (Lines 528–535), and 4.6; Conclusion (Lines 669-681).

These revisions align the mediator’s terminology, conceptual definition, measurement, empirical reporting, and theoretical interpretation throughout the manuscript. We appreciate the reviewer’s guidance, which substantially improved the construct clarity and interpretive precision of the study.

B.Clarification of the SMTQ scoring key

The omission of Item 10 from the revised description reflects the correction of a textual error in the original manuscript rather than a change in the scoring procedure. The original SMTQ contains 14 items, whereas the culturally adapted Chinese version administered in the present study contains 12 items. Because original Items 8 and 11 are not retained in the 12-item Chinese version, the subsequent items were renumbered. Specifically, the negatively worded concentration item, which is Item 10 in the original version, is Item 9 in the 12-item Chinese version. Therefore, the item numbering and reverse-scoring key of the original 14-item version cannot be directly applied to the 12-item version.

In the original manuscript, we mistakenly reported the reverse-scored item numbers of the 14-item version. In the 12-item Chinese version actually administered in this study, Items 2, 4, 7, 8, and 9 are negatively worded and were reverse-coded. Thus, the removal of Item 10 from the list of reverse-scored items in the revised manuscript reflects a correction of the original textual description. We apologize for not explaining the reason for this change in our previous response.

We rechecked the scoring procedure and confirmed that the dataset was scored according to the correct scoring key for the 12-item Chinese version. Therefore, this inconsistency was confined to the description in the original manuscript and did not affect the descriptive statistics, reliability estimates, CFA results, correlations, or PROCESS analyses.

Major Comment 2: The authors have undertaken the additional psychometric analyses requested in the previous review. The revised manuscript reports that the hypothesized five-factor model achieved acceptable fit and outperformed alternative models, and that AVE and HTMT results supported discriminant validity. The authors also added an unmeasured latent method construct model to supplement Harman’s single-factor test. These additions are directionally appropriate and represent a substantive response to my earlier comments.

However, the manuscript does not explain how the observed indicators in the CFA were constructed. The five measures contain a large number of individual items, yet the reported five-factor model has only 125 degrees of freedom. This suggests that the authors may have used subscale scores, item parcels, or another reduced-indicator approach, but the relevant procedure is not described. The authors should state clearly whether the CFA was conducted using individual items, scale dimensions, or parcels. If parcels were used, the parcel-construction procedure and its rationale should be reported, particularly because both academic burnout and athlete burnout are multidimensional constructs.

The reported baseline CFA and ULMC models also have exactly the same chi-square value, χ^2^=1127.001, despite differing by one degree of freedom. This is not necessarily impossible, but it is sufficiently unusual to require verification. The authors should confirm that the values were not duplicated inadvertently and briefly report the specification of the method factor, including whether its loadings were constrained to equality and whether the model converged without inadmissible estimates. The relevant Supplementary Tables S1–S3 should also be made fully accessible to reviewers. I do not consider it necessary to provide every line of raw software output, but the manuscript and supplement must contain enough information for readers to understand and evaluate the model specification.

The authors should additionally explain why the Harman test results changed between the original and revised manuscripts. The original manuscript reported ten factors with eigenvalues greater than one and a first-factor variance of 23.769%, whereas the revised manuscript reports nine factors and 25.214%. Because the sample remained the same, the change suggests that the set of included items, scoring, or data-processing procedure may have changed. This should be clarified so that the common-method assessment can be interpreted consistently.

**Response:** Thank you for identifying the lack of information concerning the construction of the CFA indicators and the specification of the ULMC model. We have now clarified both issues in the revised manuscript and Supplementary Materials.

First, the five-factor CFA was conducted using 18 observed indicators: three a priori dimension-level sum scores from each of the MSPSS, SMTQ, ABQ, and LBUS, together with the six individual cognitive-reappraisal items. Each dimension score was calculated according to the established structure and scoring rules of the corresponding validated subscale. Thus, these indicators were theory-based dimension-level composites rather than ad hoc or data-driven item parcels. This approach preserved the established multidimensional structures of the measures, particularly academic burnout and athlete burnout, while maintaining a parsimonious measurement model. The 18-indicator specification is also consistent with the reported 125 degrees of freedom. This information has been added to the Statistical Analysis section (Lines 303-309).

Second, we rechecked the originally reported ULMC analysis and confirmed that the identical chi-square value was not caused by inadvertent duplication. In the original ULMC model, all unstandardized method-factor loadings were constrained to equality, the variance of the method factor was fixed to 1 for identification, and its covariances with the five substantive factors were constrained to zero. Because the equality constraint introduced only one additional freely estimated loading parameter, the model had one fewer degree of freedom than the baseline CFA model. Although the software reported convergence, the common method loading was estimated as 0.000 with an extremely large standard error. The method factor therefore collapsed to a poorly determined zero-effect solution and produced no reduction in chi-square, explaining why the model yielded the same chi-square value as the baseline CFA. Accordingly, the equality-constrained model could not be regarded as a stable and substantively informative ULMC solution.

We therefore replaced the equality-constrained model with a less restrictive ULMC model in which the method-factor loadings were freely estimated on all 18 indicators. The method-factor variance was fixed to 1, and its covariances with the five substantive factors were constrained to zero. The revised model converged without negative variance estimates or standardized loadings exceeding 1.00 and showed improved fit, χ²(107) = 701.894, CFI = 0.971, TLI = 0.959, RMSEA = 0.054, and SRMR = 0.036. Inspection of the parameter estimates showed comparatively high method-factor loadings on the cognitive-reappraisal indicators, accompanied by markedly attenuated loadings on the substantive CR factor. This pattern indicated limited empirical separation between the variance attributed to the method factor and the substantive CR factor. We therefore did not attribute the improvement in model fit uniquely to common method variance. Instead, the revised manuscript now acknowledges nontrivial method-related shared covariance, particularly among the CR indicators, and notes that findings involving CR, especially the moderation effects, should be interpreted cautiously and replicated using temporally separated or multi-method designs. These revisions have been incorporated into Section 3.1(Lines 342-357) and the Limitations section (Lines 646-654 and Lines 655-661).

Supplementary Table S1 now reports the fit indices for the hypothesized five-factor CFA model, four alternative measurement models, and the freely loading ULMC model. Supplementary Table S2 presents composite reliability, AVE, construct correlations, and the square roots of AVE, whereas Supplementary Table S3 reports the HTMT ratios among the five constructs. We also added a concise summary of these results to Section 3.2 of the revised manuscript. Composite reliability values ranged from 0.770 to 0.916, AVE values ranged from 0.533 to 0.784, and HTMT ratios ranged from 0.379 to 0.727. For each construct, the square root of AVE exceeded all corresponding interconstruct correlations. Together, the composite reliability and AVE results supported convergent validity, whereas the Fornell–Larcker criterion and HTMT results supported discriminant validity. The complete results are presented in the revised Supplementary Materials.

Revisions: Results, Section 3.2 (Lines 369–373); Supplementary Tables S1–S3.

Third, the difference between the two Harman test results arose from the set of ERQ items included. In the original analysis, the expressive-suppression items, which were not part of the hypothesized model, were inadvertently included together with the cognitive-reappraisal items. The revised analysis included only the cognitive-reappraisal items, consistent with the construct examined in the present model. Apart from this adjustment to the included items, the sample, scoring procedures, data-processing criteria, and factor-extraction method remained unchanged. The revised analysis yielded nine factors with eigenvalues greater than 1, with the first factor accounting for 25.214% of the total variance. This result has been retained in the revised manuscript. We apologize for not explaining this change in our previous response.

Major Comment 3: This is a new concern arising from the revised analysis. The authors added sport item and sport level as covariates in response to concerns about sport-specific confounding, and the revised Methods section indicates that these variables were included together with gender, age, family structure, and residence. The response letter refers to the revised covariate specification around Line 303 and Table 4.

Table 4, however, appears to report only one coefficient for each of the variables family structure, residence, sport item, and sport level. The model degrees of freedom also appear consistent with each multicategory variable being entered as a single numeric predictor. If this interpretation is correct, the analysis imposes a linear and equally spaced ordering on categories that are not naturally continuous. For example, combat sports, ball sports, track and field, aquatic sports, and other sports do not constitute a meaningful numerical continuum. The same concern applies to family-structure and residence categories.

The authors should clarify exactly how these variables were coded in PROCESS. If appropriate indicator coding was already used, the reference categories and category-specific coefficients should be reported clearly in the table or Supplementary Materials. If integer codes were entered directly as single predictors, the models should be rerun using appropriate (k-1) dummy variables for nominal categories. Sport level may be treated as ordinal only if the authors can justify the assumed linear trend; otherwise, it should also be represented through indicator variables. Any required reanalysis should be reflected consistently in the mediation effects, interaction terms, confidence intervals, effect sizes, figures, and conclusions. The VIF and tolerance diagnostics should also be updated after the final coding specification is established.

Response: Thank you for raising this important methodological concern. We agree that treating multicategorical variables as single numeric predictors would impose inappropriate assumptions of linearity and equal intervals among categories.

Upon re-examination, we confirmed that family structure, residence, sport item, and sport level had previously been entered as single predictors using their original integer codes. We therefore recoded these variables using k-1 indicator variables and reran all relevant analyses. Gender was represented by one binary indicator, with male as the reference category. Family structure was represented by three indicators, with intact family as the reference category; residence by three indicators, with urban residence as the reference category; sport item by four indicators, with combat sports as the reference category; and sport level by three indicators, with National Level-2 Athlete as the reference category. Age remained a continuous covariate. Thus, no assumptions of linear ordering or equal spacing were imposed on the categorical variables.

Using the revised coding scheme, we reran the serial mediation model and the moderated serial mediation model, including all direct and indirect effects, moderated indirect effects, interaction terms, bootstrap confidence intervals, simple-slope analyses, and effect-size estimates. The revised model degrees of freedom are consistent with the final indicator coding. The overall pattern and statistical significance of the hypothesized associations remained unchanged, and the substantive conclusions were not altered. The corresponding tables, figures, and conclusions were checked and updated where necessary.

Multicollinearity diagnostics were also recalculated using the final coding specification. VIF values ranged from 1.016 to 1.686, and tolerance values ranged from 0.593 to 0.984, indicating no evidence of problematic multicollinearity.

The revisions have been incorporated throughout the manuscript. Specifically, the Statistical Analysis section (Section 2.2, Lines 318-321) was revised to clarify the coding procedures and reference categories. The serial mediation and moderated serial mediation analyses, corresponding tables, and supplementary materials (Tables S4 and S5) were updated based on the revised models.

Major Comment 4: The revised Discussion provides a much more balanced interpretation of the ATHB × CR interaction. In Lines 535–552, the authors explicitly acknowledge that cognitive reappraisal strengthened the positive association between athlete burnout and academic burnout and therefore should not be treated as a uniformly protective resource. The discussion of possible strategy exhaustion, reduced effectiveness under severe burnout, and overlap with repeated negative cognitive processing is thoughtful, provided that these explanations remain clearly identified as tentative.

This balanced interpretation is not yet fully reflected in the Abstract and Conclusion. The Abstract continues to state that CR “strengthens” the buffering effect of PSS and uses the result to support interventions combining social support enhancement with emotion-regulation training. It also states that PSS “predicted” AB despite the cross-sectional design. The Conclusion similarly tends to characterize higher CR as amplifying the protective role of PSS, without giving equal emphasis to the fact that higher CR also strengthened the positive ATHB–AB association.

The Abstract and Conclusion should explicitly state that the moderating role of CR was path-specific. Higher CR strengthened the negative PSS–AB association, but it also strengthened the positive ATHB–AB association. This dual pattern is theoretically important and should not be reduced to a general buffering interpretation. The authors should also continue revising residual causal or directional expressions such as “predicted,” “protective factor,” “reduces,” “prevents,” and “mechanism,” where these terms imply temporal or causal evidence. The serial mediation findings should be described as theory-consistent indirect associations rather than demonstrated processes of resource transformation. The practical recommendations may remain, but they should be framed as implications requiring longitudinal or experimental testing, rather than as interventions shown by the present study to be effective.

Response: Thank you for this important comment. We agree that the path-specific role of cognitive reappraisal (CR), the cross-sectional nature of the data, and the tentative status of the proposed explanations and practical implications needed to be represented more consistently throughout the manuscript. Accordingly, we made the following revisions:

First, the Abstract and Conclusion now give equal emphasis to the two significant moderation effects. Specifically, they state that, at higher levels of CR, the negative association between perceived social support (PSS) and academic burnout (AB) was stronger, but the positive association between athlete burnout (ATHB) and AB was also stronger, whereas the association between MT and AB did not differ significantly across CR levels. We therefore explicitly characterize the moderating role of CR as path-specific rather than uniformly buffering. These revisions have been incorporated into Abstract (Lines 18-21) and the Conclusion (Lines 673-677).

Second, we systematically reviewed causal and directional expressions throughout the manuscript and revised those that could imply temporal or causal evidence from the present cross-sectional findings. PSS is now described as being associated with, rather than predicting, AB. The mediation findings are described as direct and indirect associations, and the PSS → MT → ATHB → AB sequence is explicitly presented as a theory-driven statistical model rather than evidence of a causal chain or demonstrated resource-transformation process.

Third, the proposed explanations for the ATHB × CR interaction, including reduced reappraisal effectiveness under elevated burnout, strategy exhaustion, and overlap with rumination or repetitive negative processing, are now explicitly identified as speculative interpretations (see Lines 593-598).

Finally, the practical implications have been revised to avoid implying that the present study established intervention effectiveness. The manuscript now states that PSS, MT, ATHB, and CR-related training may inform future multicomponent support approaches, but that their effectiveness and boundary conditions require longitudinal or experimental evaluation. These revisions have been made in the Abstract (Lines 21–25) and the Conclusion (Lines 677–681).

Minor Comments

Minor Comment 1: The authors now clearly state that the two studies used the same cross-sectional survey and shared several variables, and they distinguish the earlier burnout-spillover model from the present resource-oriented model. The revisions around Lines 171–182 and 417–426 represent a substantial improvement and largely address my previous concern.

One further clarification would strengthen transparency. The authors should indicate whether the present moderated serial mediation model was specified before or after the analysis reported in the earlier publication, and whether any part of the current analysis should be regarded as exploratory. This does not require the authors to demonstrate formal preregistration or reconstruct a detailed historical timeline; a clear and candid statement would be sufficient.

**Response:** Thank you for this helpful suggestion. The moderated serial mediation model was developed after completion of the analyses reported in the earlier publication but before the analyses reported in the present study were conducted. The analyses were not formally preregistered. However, the focal model and Hypotheses H1–H8 were specified before the PROCESS analyses, and no component of the reported moderated serial mediation analysis was conducted as a post hoc exploratory analysis. This clarification has been added to Section 2.2 (Statistical Analysis) of the revised manuscript (Lines 325–327).

Minor Comment 2: The coefficient reported in the Abstract, β=−0.123, does not represent the total association between perceived social support and academic burnout. Rather, it represents the direct association after accounting for psychological resilience, athlete burnout, and the covariates. Table 3 reports the total effect as −0.325. The Abstract should therefore identify β=−0.123 explicitly as the direct effect or direct association, so that readers do not misinterpret it as the overall association between PSS and AB.

The manuscript should also distinguish more carefully between the interaction effect supporting H7 and the moderated indirect effects. H7 concerns whether cognitive reappraisal moderates the ATHB→AB path, and the most direct evidence for this hypothesis is the significant ATHB×CR interaction. By contrast, the conditional indirect effects of PSS on AB through ATHB at low and high levels of CR, together with the corresponding index of moderated mediation, address whether the entire PSS→ATHB→AB indirect pathway varies as a function of CR. These statistics are related, but they are not interchangeable forms of evidence for the same hypothesis.

Accordingly, the authors should report the path interaction, the conditional indirect effects, and the index of moderated mediation as conceptually distinct results. The moderated mediation index of −0.011 should be described as evidence that the PSS→ATHB→AB indirect association varies across levels of CR, rather than simply as additional support for H7. By contrast, the serial moderated mediation index of −0.017 corresponds more directly to H8, which concerns the conditional indirect association through the full PSS→PR→ATHB→AB pathway.

**Response:** Thank you for this important clarification. We agree with the reviewer and have revised both the Abstract and the Results section accordingly.

First, we revised the Abstract to explicitly identify the reported coefficient as the direct association between PSS and AB: “PSS was directly and negatively associated with AB (β = −0.122, p < 0.001).” Following the reanalysis using the revised dummy-coded covariate specification, the coefficient changed slightly from −0.123 to −0.122. The total association (β = −0.322) remains reported separately in Table 3.

Second, Section 3.4 has been revised to distinguish the evidence supporting H7 from the moderated indirect effects. The significant ATHB × CR interaction and the corresponding simple slope results are now presented as the direct evidence supporting H7. The conditional indirect effects of PSS on AB through ATHB and the index of moderated mediation (−0.011, 95% CI [−0.020, −0.004]) are reported separately as evidence that the PSS → ATHB → AB indirect pathway varied as a function of CR, rather than as additional support for H7. In contrast, the index of moderated mediation for the serial indirect effect (−0.017, 95% CI [−0.027, −0.007]) is explicitly linked to H8 because it indicates that the full PSS → MT → ATHB → AB serial indirect association varied as a function of CR. These revisions are presented in Lines 410–419.

Minor Comment 3: Although the overall language quality of the revised manuscript has improved, several clearly identifiable grammatical and formatting problems remain. For example, the phrase “CR significantly moderate” in Lines 391–393 contains an incorrect verb form, and the sentence “PSS significantly and negatively associated with AB” in Lines 621–622 is missing the auxiliary verb “was.” Lines 327–330, as well as several tables and figure notes, also contain inconsistent statistical formatting, including singular/plural errors in “p value,” spacing errors such as “p <0 .01,” mixed use of English and full-width Chinese punctuation, and duplicated periods.

The reference list also requires further checking. The same article by Shang and Yang appears to be listed repeatedly as References 34, 64, and 67. In addition, Lines 560–562 cite Reference 70 to support the statement that cognitive reappraisal did not significantly moderate the association between stress and mental health. However, the cited article concerns the mediating roles of interpersonal emotion regulation and social self-efficacy in the association between autistic traits and depression, and therefore does not directly support the claim made in the manuscript.

The authors should undertake a systematic English-language and reference audit after completing the methodological and statistical revisions. Particular attention should be paid to grammar, statistical symbols and spacing, figure-note formatting, duplicate references, and whether each cited source directly supports the corresponding statement in the text.

Response: Thank you for this careful review. We have conducted a systematic language, formatting, and reference audit throughout the manuscript. Specifically, we corrected the identified grammatical errors, including “CR significantly moderate” to “CR significantly moderated” and “PSS significantly and negatively associated with AB” to “PSS was significantly and negatively associated with AB.” We also standardized statistical reporting across the text, tables, and figure notes, including the singular/plural use of p value(s), spacing (e.g., p < 0.01), punctuation, and duplicated periods.

Third, the duplicate entries for Shang and Yang (2021), previously listed as References 34, 64, and 67, were consolidated into a single reference, and all subsequent reference numbers and corresponding in-text citations were updated. The relevant discussion was also revised to clarify that this study supports the adjacent association from social support through MT to ATHB, rather than the complete serial indirect pathway examined in the present study. Please see Lines 528–535.

We agree that the former Reference 70 did not support the original statement regarding the nonsignificant moderating role of cognitive reappraisal. The inappropriate citation and unsupported statement were therefore removed, and the discussion was revised using a more relevant, but explicitly described as partially analogous, study. Please see Lines 608- 611.

We also conducted an additional cross-check of the reference list to ensure consistency between the cited claims and their supporting sources.
