# Supplementary material for: The impact of perceived social support on college athletes’ academic burnout: The moderating role of cognitive reappraisal within a serial mediation model
Source: PLoS One. 2026 Oct 22;21(10):e0361222. doi: 10.1371/journal.pone.0361222 (PMC13690224; doi:10.1371/journal.pone.0361222)
Supplement: Supplementary file 9 [file pone.0361222.s009.docx]

Dear Editor,

Thank you very much for your careful consideration of our manuscript and for giving us the opportunity to revise it. We have carefully addressed all comments and suggestions raised by the reviewers and have provided detailed point-by-point responses. We sincerely appreciate the time and effort devoted by you and the reviewers to improving our manuscript. We hope that the revised manuscript is now suitable for publication. Should any further revisions be required, we would be pleased to address them.

Sincerely,
The Authors

Dear Reviewer,

Thank you very much for your careful review of our manuscript and for your valuable comments and suggestions. Your feedback has been highly helpful in improving the scientific rigor, clarity, and overall quality of the manuscript. We have carefully considered and addressed each of your comments and revised the manuscript accordingly. All changes have been highlighted in the revised manuscript for ease of reference. We sincerely appreciate the time and effort you devoted to evaluating our work. Should any further revisions or clarifications be required, we would be pleased to address them.

Sincerely,
The Authors

Minor Concerns

Q1:Lines 218–220 state that 2,115 questionnaires were distributed and 1,967 were returned, corresponding to a response rate of 93.00%. Lines 220–222 further report that 49 questionnaires were excluded and that 1,918 respondents were retained, but describe 97.51% as the “effective response rate.” The latter percentage represents the proportion of usable questionnaires among the questionnaires returned (1,918/1,967), whereas the proportion of usable questionnaires among all questionnaires distributed is 90.69% (1,918/2,115). Please revise the terminology at Lines 218–222 to distinguish clearly between the questionnaire return rate, the proportion of usable questionnaires among returned questionnaires, and the overall usable response rate.

Lines 220–221 also refer to “repetitive patterns” and “logical errors” as exclusion criteria. Please briefly clarify how these criteria were defined and applied during data screening.

Response: Thank you for this clarification. We have revised the participant-screening description to distinguish clearly among the questionnaire return rate, the proportion of usable questionnaires among returned questionnaires, and the overall usable response rate. Specifically, 1,967 of the 2,115 distributed questionnaires were returned (return rate: 93.00%), and 1,918 were retained for analysis, corresponding to 97.51% of the returned questionnaires and an overall usable response rate of 90.69%.

We also clarified the exclusion criteria used during manual screening. “Repetitive patterns” defined as invariant responding within all multi-item scales included in the questionnaire“Logical errors” has been revised to logical inconsistencies and was operationalized as contradictory responses regarding sport-specific training, such as reporting no weekly sport-specific training while also reporting at least one training day per week or at least 5 hours of weekly sport-specific training. These revisions have been incorporated into the Methods section. (Lines 218–226).

Q2:At Line 190, H1 is formulated as the hypothesis that PSS is negatively correlated with AB. The zero-order correlation reported in Table 2 at Line 356 ((r=-0.327), (p<0.01)) provides the direct evidence relevant to this hypothesis. However, Lines 371–372 state that the adjusted direct association between PSS and AB (effect = −0.122, 95% CI [−0.170, −0.073]) supports H1.

These two statistics represent different quantities. Please revise Lines 371–372 so that the correlation reported in Table 2 is used as the primary evidence for H1. The coefficient reported in Table 3 should be identified separately as the direct association between PSS and AB after accounting for the proposed mediators and covariates.

Response: Thank you for this important clarification. We agree that the zero-order Pearson correlation reported in Table 2 provides the evidence directly relevant to H1, whereas the coefficient reported in Table 3 represents the direct association between PSS and AB after accounting for the proposed mediators and covariates. Accordingly, we have revised Sections 3.2 and 3.3. H1 is now evaluated using the zero-order Pearson correlation between PSS and AB reported in Table 2 (r = −0.327, p < 0.01). The coefficient reported in Table 3 is now described separately as the significant negative direct association between PSS and AB after accounting for MT, ATHB, and the covariates (direct effect = −0.122, 95% CI [−0.170, −0.073]). The previous statement that this adjusted direct effect supported H1 has been removed. (Lines 352–353 and 375–377)

Q3. Lines 257–263 explain that the ERQ comprises the cognitive reappraisal and expressive suppression dimensions, while only the CR subscale was included in the model. Lines 266–267 subsequently report Cronbach’s (Cronbach’s α=0.828) for the ERQ and (Cronbach’s α=0.894) for CR. It is unclear which items were included in the calculation of the first coefficient and why the reliability of the complete ERQ is relevant when only CR was analyzed.

Please clarify at Lines 266–267 which items were used to calculate each reliability coefficient. Because only the CR subscale was included in the reported analyses, its reliability estimate should be emphasized. The reliability estimate for the complete ERQ should be removed if it is not relevant to the present analysis.

Response: Thank you for this comment. We have removed the reliability estimate for the full ERQ (α = 0.828), as only the CR subscale was analyzed. The revised text clarifies that α = 0.894 was calculated using the six CR items (items 1, 3, 5, 7, 8, and 10). (Lines 270–272).

Q4. At Line 5, the institutional affiliation is not capitalized consistently. Please conduct a final systematic language and statistical-formatting check across the main text, tables, figure captions, and notes.

Response: Thank you for this careful observation. We have corrected and standardized the capitalization of the institutional affiliation. We also conducted a final systematic check of language and statistical formatting throughout the manuscript, including the main text, tables, figure captions, and notes.
