# Supplementary material for: The impact of perceived social support on college athletes’ academic burnout: The moderating role of cognitive reappraisal within a serial mediation model
Source: PLoS One. 2026 Oct 22;21(10):e0361222. doi: 10.1371/journal.pone.0361222 (PMC13690224; doi:10.1371/journal.pone.0361222)
Supplement: Peer review history

# Peer review: Decision Letter 0

**DOI:** 10.1371/journal.pone.0361222.r001

**Authors:** Rogis Baker

29 Apr 2026

Dear Dr. Xu,

Thank you for submitting your manuscript to PLOS ONE. After careful consideration, we feel that it has merit but does not fully meet PLOS ONE’s publication criteria as it currently stands. Therefore, we invite you to submit a revised version of the manuscript that addresses the points raised during the review process.

Please submit your revised manuscript by Jun 13 2026 11:59PM. If you will need more time than this to complete your revisions, please reply to this message or contact the journal office at plosone@plos.org. When you're ready to submit your revision, log on to https://www.editorialmanager.com/pone/ and select the 'Submissions Needing Revision' folder to locate your manuscript file.

- A letter that responds to each point raised by the academic editor and reviewer(s). You should upload this letter as a separate file labeled 'Response to Reviewers'.
- A marked-up copy of your manuscript that highlights changes made to the original version. You should upload this as a separate file labeled 'Revised Manuscript with Track Changes'.
- An unmarked version of your revised paper without tracked changes. You should upload this as a separate file labeled 'Manuscript'.

If you would like to make changes to your financial disclosure, please include your updated statement in your cover letter. Guidelines for resubmitting your figure files are available below the reviewer comments at the end of this letter.

If applicable, we recommend that you deposit your laboratory protocols in protocols.io to enhance the reproducibility of your results. Protocols.io assigns your protocol its own identifier (DOI) so that it can be cited independently in the future. For instructions see: https://journals.plos.org/plosone/s/submission-guidelines#loc-laboratory-protocols. Additionally, PLOS ONE offers an option for publishing peer-reviewed Lab Protocol articles, which describe protocols hosted on protocols.io. Read more information on sharing protocols at https://plos.org/protocols?utm_medium=editorial-email&utm_source=authorletters&utm_campaign=protocols.

As the corresponding author, your ORCID iD is verified in the submission system and will appear in the published article. PLOS supports the use of ORCID, and we encourage all coauthors to register for an ORCID iD and use it as well. Please encourage your coauthors to verify their ORCID iD within the submission system before final acceptance, as unverified ORCID iDs will not appear in the published article. *Only*  the individual author can complete the verification step; PLOS staff *cannot*  verify ORCID iDs on behalf of authors.

We look forward to receiving your revised manuscript.

Kind regards,

Rogis Baker, Ph.D

Academic Editor

PLOS One

Journal Requirements:

When submitting your revision, we need you to address these additional requirements.

1.Please ensure that your manuscript meets PLOS ONE's style requirements, including those for file naming. The PLOS ONE style templates can be found at

https://journals.plos.org/plosone/s/file?id=wjVg/PLOSOne_formatting_sample_main_body.pdf and

https://journals.plos.org/plosone/s/file?id=ba62/PLOSOne_formatting_sample_title_authors_affiliations.pdf

2. Your ethics statement should only appear in the Methods section of your manuscript. If your ethics statement is written in any section besides the Methods, please delete it from any other section.

3. We note that there is identifying data in the Supporting Information file “Data.xlsx”. Due to the inclusion of these potentially identifying data, we have removed this file from your file inventory. Prior to sharing human research participant data, authors should consult with an ethics committee to ensure data are shared in accordance with participant consent and all applicable local laws.

Data sharing should never compromise participant privacy. It is therefore not appropriate to publicly share personally identifiable data on human research participants. The following are examples of data that should not be shared:

-Name, initials, physical address

-Ages more specific than whole numbers

-Internet protocol (IP) address

-Specific dates (birth dates, death dates, examination dates, etc.)

-Contact information such as phone number or email address

-Location data

-ID numbers that seem specific (long numbers, include initials, titled “Hospital ID”) rather than random (small numbers in numerical order)

Data that are not directly identifying may also be inappropriate to share, as in combination they can become identifying. For example, data collected from a small group of participants, vulnerable populations, or private groups should not be shared if they involve indirect identifiers (such as sex, ethnicity, location, etc.) that may risk the identification of study participants.

Additional guidance on preparing raw data for publication can be found in our Data Policy (https://journals.plos.org/plosone/s/data-availability#loc-human-research-participant-data-and-other-sensitive-data) and in the following article: http://www.bmj.com/content/340/bmj.c181.long.

Please remove or anonymize all personal information (<specific identifying information in file to be removed>), ensure that the data shared are in accordance with participant consent, and re-upload a fully anonymized data set. Please note that spreadsheet columns with personal information must be removed and not hidden as all hidden columns will appear in the published file.

4. If the reviewer comments include a recommendation to cite specific previously published works, please review and evaluate these publications to determine whether they are relevant and should be cited. There is no requirement to cite these works unless the editor has indicated otherwise.

[Note: HTML markup is below. Please do not edit.]

Reviewers' comments:

Reviewer's Responses to Questions

**Comments to the Author**

1. Is the manuscript technically sound, and do the data support the conclusions?

Reviewer #1: Yes

Reviewer #2: Partly

**********

2. Has the statistical analysis been performed appropriately and rigorously?

Reviewer #1: Yes

Reviewer #2: Yes

**********

3. Have the authors made all data underlying the findings in their manuscript fully available?

The PLOS Data policy

Reviewer #1: Yes

Reviewer #2: Yes

**********

4. Is the manuscript presented in an intelligible fashion and written in standard English?

Reviewer #1: Yes

Reviewer #2: Yes

**********

Reviewer #1: This manuscript presents an interesting investigation integrating perceived social support (PSS), psychological resilience (PR), athlete burnout (ATHB), and cognitive reappraisal (CR) to understand academic burnout (AB) in college athletes. The focus on the dual-stressor population and cross-domain burnout spillover is highly valuable, and the authors' inclusion of the raw dataset in the supplementary files is commendable and aligns well with PLOS ONE’s open science standards. However, there are critical methodological, statistical, and conceptual issues that must be rigorously addressed to meet the publication criteria of PLOS ONE.

1. I appreciate the authors' transparency in disclosing that the dataset used in this study was collected from the same cohort as their recently published research. However, upon reviewing the published paper, I noticed a substantial conceptual and structural overlap between the two studies that requires immediate clarification. Both studies utilize the exact same cross-sectional data to explore the interrelationships among Athlete Burnout, Academic Burnout, and Cognitive Reappraisal. In the published work, Athlete Burnout predicts Academic Burnout with Cognitive Reappraisal acting as a mediator. In the current manuscript, Athlete Burnout is positioned as a mediator, while Cognitive Reappraisal serves as a moderator. Reconfiguring the statistical roles of the same core variables from a single survey administration raises significant questions about the distinct theoretical contribution of this second paper. To satisfy the publication standards of PLOS ONE, I strongly encourage the authors to explicitly clarify the incremental value of this manuscript in the Introduction and Discussion sections, justifying why these variables were not integrated into a single, comprehensive model originally.

2. Table 1 details important athletic characteristics such as "Sports items" (combat, ball, track) and "Sport level." Strikingly, these critical variables were completely excluded as covariates in the mediation models. Athlete burnout and psychological resilience are highly sensitive to the competitive level and the specific nature of the sport (e.g., individual versus team sports). Omitting these sport-specific covariates undermines the validity of the findings. The authors should rerun the analyses controlling for these athletic characteristics or thoroughly justify their exclusion.

3. The reliance on Harman’s single-factor test to address Common Method Bias (CMB) is insufficient by modern psychometric standards. Harman’s test is widely considered an insensitive diagnostic tool. Given that all data are cross-sectional self-reports, the authors must employ more robust methods to evaluate CMB, such as conducting a Confirmatory Factor Analysis (CFA) with an unmeasured latent method factor (ULMC) or using a theoretically unrelated marker variable.

4. The study focuses on Cognitive Reappraisal (CR) as the sole emotion regulation moderator. While theoretically sound, CR is strictly an intrapersonal (internal) emotion regulation strategy. Given that the primary predictor—Perceived Social Support—is an inherently interpersonal external resource, relying exclusively on an internal coping mechanism creates a conceptual gap. The authors should expand their discussion to acknowledge the limitation of only assessing intrapersonal regulation and discuss how interpersonal emotion regulation strategies might synergize with perceived social support to combat burnout. For context, the authors might refer to recent literature such as "The Association Between Autistic Traits and Depression in College Students: The Mediating Roles of Interpersonal Emotion Regulation and Social Self-Efficacy" to explore how interpersonal emotion regulation functions alongside social variables.

5. The authors utilized a single composite score for both Academic Burnout (AB) and Athlete Burnout (ATHB). However, burnout is universally recognized as a multidimensional syndrome (e.g., emotional exhaustion versus diminished accomplishment). Combining these distinct dimensions into a single construct may mask specific, nuanced mechanisms. Advanced mediation models often deconstruct the dependent variable to reveal dimension-specific pathways. The authors should justify their use of a total score, or ideally, conduct an exploratory path analysis examining the specific dimensions of burnout.

6.Due to the cross-sectional nature of the data, the serial mediation model implies a directional flow that cannot be definitively confirmed. Causal language (e.g., "how PSS influences AB," "PSS alleviates AB") should be tempered carefully throughout the abstract and discussion, explicitly framing the results as associative rather than causal.

7. The path diagrams (Figures 1, 2, and 3) appear somewhat rudimentary and visually disjointed. Redesigning these flowcharts for improved aesthetic clarity and ensuring they meet PLOS ONE’s high-resolution figure guidelines will significantly improve readability. Finally, the discussion on practical interventions remains somewhat generic; please elaborate on how athletic departments and coaches can tangibly implement the suggested emotion regulation training in a dual-career student-athlete context.

Reviewer #2: Specific Comments

Q1. The authors acknowledge in Lines 206–209 that the dataset used in the present study was collected from the same cohort of college athletes as their previously published study (Reference 33: Xu et al., 2025, Frontiers in Psychology). The earlier study and the present manuscript appear to share several closely related elements: academic burnout serves as the dependent variable in both papers, athlete burnout is a key variable in both, and emotion regulation/cognitive reappraisal is involved in both analytical frameworks.

I do not suggest that the use of the same cohort across multiple studies is inherently inappropriate. However, when the same participant cohort and closely related psychological variables are used across separate manuscripts, the relationship between the studies should be disclosed with greater transparency. At present, the manuscript addresses this issue only briefly, stating that the research objectives, variable selection, and analytical framework are different. This statement is helpful, but it does not provide enough detail for readers to evaluate the degree of overlap and the distinct contribution of the present manuscript.

I recommend that the authors add a brief clarification in the Methods section, or in the supplementary materials if space is limited, explaining the relationship between the present study and the previously published paper. This clarification should specify which variables overlap across the two studies, whether the relevant measures were collected during the same survey administration, and what distinct theoretical or analytical contribution the present manuscript makes beyond the prior publication.

Q2. Lines 258–268 describe the Sports Mental Toughness Questionnaire (SMTQ), including its origin, dimensional structure, scoring method, and internal reliability. However, in the correlation matrix and subsequent mediation and moderation models, the corresponding construct is labeled as psychological resilience (PR), rather than mental toughness. This creates conceptual and methodological ambiguity.

The authors should explicitly state whether the SMTQ total score was used as the operational indicator of PR. If so, this operational decision requires clear justification, because mental toughness and psychological resilience are related but not identical constructs. The Methods section should explain why a measure originally designed to assess sports mental toughness can validly represent psychological resilience in the present model. The Results section should also make the naming consistent, so that readers can clearly understand whether the construct being tested is mental toughness, psychological resilience, or a theoretically justified proxy for resilience.

At present, the manuscript appears to move from the measurement label “sports mental toughness” to the analytical label “psychological resilience” without sufficient explanation. This should be clarified before readers can properly evaluate the validity of the mediation model.

Q3.Related to the previous point, the manuscript repeatedly frames the relevant mediator as “psychological resilience,” citing literature that defines resilience as the capacity for recovery, adaptation, and sustained functioning under adversity. However, the actual measurement instrument appears to be the SMTQ, which was designed to assess sports mental toughness rather than resilience per se.

Although mental toughness and psychological resilience overlap in sport psychology, they should not be treated as fully interchangeable without theoretical or empirical justification. Resilience generally emphasizes recovery, adaptation, and positive functioning after adversity or stress exposure, whereas mental toughness more strongly emphasizes confidence, constancy, persistence, and perceived control under pressure. These conceptual differences matter because the manuscript interprets the mediator as an internal psychological resource that is built through perceived social support and then reduces athlete burnout and academic burnout.

I recognize that the data have already been collected and that it may not be feasible to re-administer an alternative resilience measure, such as the Connor-Davidson Resilience Scale, to the same 1,918 participants. Therefore, I do not require the authors to replace the measure. However, the authors should address this construct-validity issue in three specific ways.

First, in the Methods section, the authors should add a paragraph justifying the use of the SMTQ to operationalize psychological resilience. This justification should cite sport psychology literature showing the conceptual and empirical overlap between mental toughness and resilience, especially in athlete populations.

Second, in the Limitations section, the authors should explicitly acknowledge that the SMTQ was not originally designed to measure psychological resilience per se. The authors should explain that this operationalization choice may affect the interpretation of the PR-related findings and that future research using dedicated resilience measures would be necessary to verify the robustness of the current mediation results.

Third, the authors should provide additional evidence that the construct measured by the SMTQ is empirically distinguishable from the other constructs in the model. This is especially important given the relatively high correlations among some self-report variables, such as the correlation between PR and CR. A confirmatory factor analysis, or another appropriate test of discriminant validity, would help demonstrate that the SMTQ-based mediator captures a distinct construct rather than reflecting a broader general psychological adjustment factor.

Without these clarifications, the current manuscript risks overstating the evidence for a psychological resilience mechanism when the empirical measure may more accurately reflect sports mental toughness. This does not necessarily invalidate the findings, but it does require a more cautious and transparent interpretation.

Q4. The study employs five self-report instruments simultaneously, with notably high inter-correlations among several variables. For example, Table 2 reports relatively high correlations between PSS and CR (r = 0.658) and between PR and CR (r = 0.560). Under these circumstances, it would be important to provide CFA results, or other appropriate evidence of discriminant validity, to confirm that the five constructs are empirically distinguishable.

At present, the manuscript reports internal consistency coefficients and bivariate correlations, but it does not provide sufficient evidence that the measured constructs are distinct from one another. The particularly high correlation between PSS and CR raises the question of whether these two variables may partly reflect overlapping aspects of a broader positive psychological adjustment factor. I recommend that the authors report additional evidence of construct distinctiveness, such as confirmatory factor analysis, comparison of alternative factor models, AVE-based discriminant validity, HTMT, or another appropriate approach.

Q5. Table 3 reports the 95% confidence interval for the PSS → ATHB → AB indirect effect as [−0.065, −0.028], whereas Line 312 in the text reports the same result as [−0.064, −0.027]. Both the lower and upper bounds differ. Although the discrepancy is numerically small, reporting different values for the same result within the same manuscript undermines the credibility of the data. The authors should verify the original PROCESS output and ensure consistency throughout.

Q6. Figure 2 presents standardized path coefficients from the serial mediation model (Model 6), such as PR → AB = −0.150 and ATHB → AB = 0.327. These values differ from those in Table 4, which reports Model 89 coefficients (−0.108 and 0.302, respectively). I understand this discrepancy arises because the two models include different sets of predictors, which is expected. However, the authors never provide the full regression coefficient table for Model 6. Only the indirect effects appear in Table 3, leaving the path coefficients shown in Figure 2 unverifiable against any tabulated data. I recommend adding a complete Model 6 regression table in the main text or supplementary materials.

Q7. Table 4 reports the p-value for the PSS × CR interaction term as p = 0.010, while Line 327 describes the same result as “p < 0.05.” The manuscript alternates between exact p-values and inequality notation throughout. I recommend adopting a uniform reporting standard.

Q8. When the correlation between an independent variable and a moderator reaches 0.66, interaction term estimates may become unstable. The authors note in Lines 283–284 that variables were mean-centered prior to computing interaction terms, which is appropriate. However, mean-centering only reduces collinearity between the interaction term and the constituent main effects; it does not address collinearity between the main effects themselves. I recommend reporting VIF (Variance Inflation Factor) values for each predictor across all models.

Q9. Lines 287–294 report that the first unrotated factor explained 23.769% of the variance, below the 40% threshold. However, Harman’s test has been widely criticized in the methodological literature for its low sensitivity and limited statistical power. I recommend that the authors at minimum acknowledge this limitation, and ideally supplement the test with a more robust approach, such as adding a common method factor within the CFA framework.

Q10. The moderated serial mediation model tested in the manuscript specifies CR as a moderator of paths leading to AB, including the PSS → AB, PR → AB, and ATHB → AB paths. This specification is theoretically plausible, but the manuscript does not sufficiently justify why CR was modeled only at these later-stage paths rather than at earlier stages of the serial chain, such as PSS → PR, PSS → ATHB, or PR → ATHB.

Given that the Introduction describes CR as a factor that may influence the transformation of external support into internal psychological resources, one might also expect CR to moderate earlier resource-transformation paths. I recommend that the authors provide a clearer theoretical rationale for the chosen PROCESS Model 89 specification and acknowledge that alternative moderated mediation models could test different boundary conditions within the serial pathway.

Q11. Table 4 and Figure 5 show that CR positively moderates the ATHB → AB path (β = 0.078, p < 0.001). The simple slope analysis indicates that the association between ATHB and AB is stronger among athletes with higher CR (β = 0.380) than among those with lower CR (β = 0.223). In other words, higher CR is associated with a stronger ATHB–AB link, rather than a weaker one.

This finding complicates, and partly contradicts, the manuscript’s general framing of CR as a uniformly protective factor. Although CR appears to strengthen the protective association between PSS and AB, its moderating role in the ATHB → AB path operates in the opposite direction from what a simple protective-factor interpretation would predict.

The authors briefly touch on this result in Lines 476–479 but do not meaningfully engage with its theoretical implications. I believe this finding warrants a more thorough discussion. Possible explanations might include: (a) cognitive reappraisal may be effective under mild stress but may fail or even backfire when athletic burnout reaches severe levels, with high-CR individuals becoming more acutely aware of their deteriorating state; (b) high-CR individuals may over-rely on reappraisal strategies while neglecting problem-focused coping, leading to strategy exhaustion when burnout accumulates; or (c) there may be blurred boundaries between reappraisal and rumination, such that some individuals’ “reappraisal” effectively constitutes repeated cognitive processing of negative experiences. These possibilities deserve serious discussion rather than a cursory mention.

In its current form, the Discussion risks overstating the protective role of CR and underinterpreting a theoretically important, counterintuitive moderation effect.

Q12. The simple slope analysis for the PSS × CR interaction suggests that, among athletes with lower CR, the protective association between PSS and AB is weaker and does not reach conventional statistical significance (effect = −0.058, p = 0.066). By contrast, the association is stronger and statistically significant among athletes with higher CR (effect = −0.162, p < 0.001).

This pattern has important implications for intervention design. It suggests that enhancing social support alone may be less effective for athletes who have lower levels of cognitive reappraisal, and that social support interventions may need to be combined with emotion regulation training to produce more consistent benefits for reducing academic burnout.

However, the manuscript’s discussion of this point is too brief. I recommend that the authors elaborate on the practical implications of this conditional effect, especially with regard to how universities, coaches, counselors, and academic support staff might design interventions that integrate social support enhancement with emotion regulation training.

Q13. The AB equation in Table 4 explains 25.9% of the variance in academic burnout. While this represents a meaningful proportion of explained variance in psychological research, the manuscript does not report individual predictor effect sizes or discuss the practical magnitude of the observed associations. I recommend that the authors supplement the statistical significance tests with effect size indicators where appropriate, such as semi-partial effects, standardized effect interpretation, or f² values. More importantly, the Discussion should clarify what the observed effects mean for practical intervention design. For example, the authors should explain whether the effects of PSS, ATHB, and CR are sufficiently large to support targeted intervention programs, or whether the findings should be interpreted as preliminary evidence requiring further validation.

Q14. The authors successively draw upon the stress-buffering model (Line 91), Conservation of Resources theory (Line 106), the process model of emotion regulation (Line 151), and later in the Discussion introduce the Job Demands–Resources model (Line 448) and self-regulation theory (Line 470). While each framework has some relevance, the current presentation lacks a unifying logical thread and reads more like a list of theories than an integrated framework. I recommend anchoring the paper in COR theory as the primary framework and clearly positioning the other theories in supporting roles.

Q15. The proposed sequence assumes that social support builds resilience, which in turn reduces athletic burnout, and the reduction in athletic burnout then lowers academic burnout. However, under a cross-sectional design, alternative causal orderings are equally plausible. For example, ATHB → PR → AB (athletic burnout erodes resilience, which then exacerbates academic burnout) is theoretically defensible. I recommend that the authors provide more thorough theoretical justification for the chosen ordering in the Introduction, and indicate whether any competing models were tested. If not, this should be acknowledged in the Limitations section.

Q16. H7 reads “CR moderates the relationship between ABTH and AB.” The abbreviation should be “ATHB,” not “ABTH.” Please also check the x-axis label of Figure 5 and all related figure labels for abbreviation consistency, and correct this issue throughout the manuscript.

Q17. Lines 386–389 discuss how PSS may modulate the sympathetic nervous system and the HPA axis to reduce physiological stress responses. However, the present study collected no physiological data whatsoever. While citing relevant literature as theoretical background is acceptable, the current phrasing risks misleading readers into thinking these mechanisms are findings of the present study. I recommend adjusting the wording to clearly mark these as speculative interpretations based on prior literature.

Q18. The Limitations discussion (Lines 496-509) covers cross-sectional design, self-report instruments, and the non-significant PR × CR interaction, which is reasonable. However, it omits several points worth discussing: (a) the sample was recruited through convenience sampling from Chinese university sports teams, and the representativeness of the sample may be limited, particularly given the uneven distribution of sport types and athlete levels; (b) using the SMTQ to operationalize psychological resilience may introduce construct validity concerns (see Q3); and (c) the high collinearity between PSS and CR (r = 0.658) may affect the stability of interaction term estimates (see Q8).

Q19. The reference list uses journal abbreviations for some entries and full journal names for others; some entries include complete page information while others provide only article numbers; and the formatting of translated Chinese-language references is inconsistent with other entries. Please revise in accordance with PLOS ONE reference formatting requirements.

Q20. After a careful reading of the Introduction, I find that the empirical grounding for the hypotheses is uneven. H1 and H2 are reasonably well supported by cited literature. H3, however, relies on the ATHB → AB spillover assumption, for which the only cited evidence is the authors’ own prior work (Reference 33, Line 142: “our preliminary work suggests”). A single self-citation is insufficient to anchor a mediation hypothesis; the authors should supplement this with independent evidence on cross-domain burnout transfer.

H4, the core serial mediation hypothesis, lacks direct empirical precedent. No cited study has tested a sequential PSS → PR → ATHB → AB pathway, and the critical PR → ATHB link receives only a brief mention at Lines 136–138 (Reference 29), citing a correlational association rather than a mediation relationship. A four-variable serial mediation hypothesis requires stronger literature support than what is currently provided.

Most critically, the moderation hypotheses H5 through H8 (Lines 176–179) suffer from a systematic logical gap. The authors’ argument in Lines 154–161 proceeds as follows: CR is related to AB (Reference 36), CR is related to resilience (Reference 37), and CR is related to ATHB (Reference 38). From these bivariate associations, the authors derive hypotheses that CR moderates specific paths (PSS → AB, PR → AB, ATHB → AB, and the serial indirect effect). However, "CR correlates with variable X" does not logically entail "CR moderates the path from X to Y." Correlation and moderation are distinct statistical and conceptual relationships. Furthermore, Reference 36 actually examines CR as a mediator between social support and academic burnout, not as a moderator. Using a mediation finding to justify a moderation hypothesis conflates two different theoretical mechanisms. I recommend that the authors either locate prior studies that have specifically tested CR (or related emotion regulation strategies) as a moderator of burnout pathways, or reframe the moderation hypotheses as more exploratory in nature and adjust the language in the Introduction accordingly.

**********

what does this mean?). If published, this will include your full peer review and any attached files.

If you choose “no”, your identity will remain anonymous but your review may still be made public.

**Do you want your identity to be public for this peer review?**  For information about this choice, including consent withdrawal, please see our Privacy Policy

Reviewer #1: No

Reviewer #2: No

**********

[NOTE: If reviewer comments were submitted as an attachment file, they will be attached to this email and accessible via the submission site. Please log into your account, locate the manuscript record, and check for the action link "View Attachments". If this link does not appear, there are no attachment files.]

To ensure your figures meet our technical requirements, please review our figure guidelines: https://journals.plos.org/plosone/s/figures

You may also use PLOS’s free figure tool, NAAS, to help you prepare publication quality figures: https://journals.plos.org/plosone/s/figures#loc-tools-for-figure-preparation.

NAAS will assess whether your figures meet our technical requirements by comparing each figure against our figure specifications.

Attachment Submitted filename: Peer Review Report PONE-D-26-07482 .pdf

# Author comment: Author response to Decision Letter 1

**DOI:** 10.1371/journal.pone.0361222.r002

10 Jun 2026

Response to Editor

Dear Editor,

First, we would like to sincerely thank you for your time and effort in handling our manuscript, as well as for granting us sufficient time to complete the revisions. We have carefully revised the manuscript according to the reviewers’ comments and have provided detailed point-by-point responses to each reviewer’s comments below.

To facilitate your and the reviewers’ evaluation, we have uploaded two versions of the revised manuscript: one with tracked changes and one clean version without tracked changes. We hope that the revised manuscript and our responses adequately address the reviewers’ concerns. If any further revisions or clarifications are needed, we would be very willing to make additional improvements.

Sincerely,

The Authors

Response to Reviewer 1

Dear Reviewer,

We sincerely thank you for your positive evaluation of our work and for your professional, constructive, and detailed comments. We greatly appreciate the time and effort you devoted to reviewing our manuscript. We believe that your insightful suggestions have helped us substantially improve the quality, clarity, and rigor of the manuscript.

We have carefully considered each of your comments and have revised the manuscript accordingly. Detailed point-by-point responses are provided below.

Sincerely,

The Authors

Q1: appreciate the authors' transparency in disclosing that the dataset used in this study was collected from the same cohort as their recently published research. However, upon reviewing the published paper, I noticed a substantial conceptual and structural overlap between the two studies that requires immediate clarification. Both studies utilize the exact same cross-sectional data to explore the interrelationships among Athlete Burnout, Academic Burnout, and Cognitive Reappraisal. In the published work, Athlete Burnout predicts Academic Burnout with Cognitive Reappraisal acting as a mediator. In the current manuscript, Athlete Burnout is positioned as a mediator, while Cognitive Reappraisal serves as a moderator. Reconfiguring the statistical roles of the same core variables from a single survey administration raises significant questions about the distinct theoretical contribution of this second paper. To satisfy the publication standards of PLOS ONE, I strongly encourage the authors to explicitly clarify the incremental value of this manuscript in the Introduction and Discussion sections, justifying why these variables were not integrated into a single, comprehensive model originally.

Response:Thank you for this professional and constructive suggestion. In the revised manuscript, we have explicitly clarified that the two studies used the same cross-sectional survey data from the same cohort of participants and included some overlapping variables. However, the two studies addressed different theoretical questions.

Specifically, our published article examined the burnout spillover process in which athlete burnout was associated with academic burnout through cognitive reappraisal and sleep quality. In contrast, the present study is grounded in Conservation of Resources theory and treats perceived social support as the predictor, psychological resilience and athlete burnout as sequential mediators, and cognitive reappraisal as a moderating emotion-regulation resource.

Therefore, the incremental contribution of the present study lies in shifting the research focus from the burnout spillover process to a resource-oriented explanation; that is, how perceived social support is associated with academic burnout through the sequential pathway of psychological resilience and athlete burnout, and how this process varies according to athletes’ levels of cognitive reappraisal.

We have also further explained why these variables were not integrated into one comprehensive model. The two studies were designed to answer different theoretical questions. Including perceived social support, psychological resilience, athlete burnout, cognitive reappraisal, sleep quality, and academic burnout in a single comprehensive model would substantially increase model complexity and weaken the theoretical focus of the specific mechanisms examined in each study. Therefore, we tested two theoretically distinct models with clear boundaries to maintain theoretical clarity and avoid overcomplicating the model. Please refer to Lines 177–188 and 440–446 in the revised manuscript.

Q2. Table 1 details important athletic characteristics such as "Sports items" (combat, ball, track) and "Sport level." Strikingly, these critical variables were completely excluded as covariates in the mediation models. Athlete burnout and psychological resilience are highly sensitive to the competitive level and the specific nature of the sport (e.g., individual versus team sports). Omitting these sport-specific covariates undermines the validity of the findings. The authors should rerun the analyses controlling for these athletic characteristics or thoroughly justify their exclusion.

Response:Thank you for this helpful suggestion. We reran the mediation model with sport type and competitive level included as additional covariates. The results showed that the main hypothesized paths remained significant, indicating that the indirect association between perceived social support and academic burnout through psychological resilience and athlete burnout was robust after controlling for these sport-specific characteristics.

We have clarified the covariates in the Methods section, updated the Results section accordingly, and noted in the relevant tables/figure notes that sport type and competitive level were controlled. Please refer to Line 309 and Table 4 in the revised manuscript.

Q3.The reliance on Harman’s single-factor test to address Common Method Bias (CMB) is insufficient by modern psychometric standards. Harman’s test is widely considered an insensitive diagnostic tool. Given that all data are cross-sectional self-reports, the authors must employ more robust methods to evaluate CMB, such as conducting a Confirmatory Factor Analysis (CFA) with an unmeasured latent method factor (ULMC) or using a theoretically unrelated marker variable.

Response:Thank you for this valuable comment. We agree with the reviewer that Harman’s single-factor test has limitations and should not be used as the sole evidence for assessing common method bias. In response to this comment, we supplemented the revised manuscript with CFA-based tests of common method bias, including comparisons with alternative models and an unmeasured latent method construct (ULMC) model.

The hypothesized five-factor CFA model showed better fit than the alternative models (χ² = 1127.001, df = 125, CFI = 0.952, TLI = 0.941, RMSEA = 0.065, SRMR = 0.050). After adding an unmeasured latent method factor with equal loadings on all observed indicators, the ULMC model showed no substantive improvement in fit (χ² = 1127.001, df = 124, CFI = 0.952, TLI = 0.940, RMSEA = 0.065). These results suggest that common method bias was unlikely to substantially affect the findings of this study.Please refer to Line 323-331 in the revised manuscript and Supplementary Table S1.

Q4.The study focuses on Cognitive Reappraisal (CR) as the sole emotion regulation moderator. While theoretically sound, CR is strictly an intrapersonal (internal) emotion regulation strategy. Given that the primary predictor—Perceived Social Support—is an inherently interpersonal external resource, relying exclusively on an internal coping mechanism creates a conceptual gap. The authors should expand their discussion to acknowledge the limitation of only assessing intrapersonal regulation and discuss how interpersonal emotion regulation strategies might synergize with perceived social support to combat burnout. For context, the authors might refer to recent literature such as "The Association Between Autistic Traits and Depression in College Students: The Mediating Roles of Interpersonal Emotion Regulation and Social Self-Efficacy" to explore how interpersonal emotion regulation functions alongside social variables.

Response: Thank you for this professional and constructive suggestion. In response to the reviewer’s comment, we have added a discussion on how interpersonal emotion-regulation strategies may work together with perceived social support to address burnout. This revision has been incorporated into the Discussion section (Lines 525–534). We have also explicitly acknowledged this limitation in the Limitations section (Lines 619–624).

Q5. The authors utilized a single composite score for both Academic Burnout (AB) and Athlete Burnout (ATHB). However, burnout is universally recognized as a multidimensional syndrome (e.g., emotional exhaustion versus diminished accomplishment). Combining these distinct dimensions into a single construct may mask specific, nuanced mechanisms. Advanced mediation models often deconstruct the dependent variable to reveal dimension-specific pathways. The authors should justify their use of a total score, or ideally, conduct an exploratory path analysis examining the specific dimensions of burnout.

Response:Thank you for this important suggestion. We agree that both academic burnout and athlete burnout have multidimensional structures, and dimension-level analyses may reveal more nuanced mechanisms. However, the present study used total scores mainly because the proposed model focused on the overall levels of academic burnout and athlete burnout rather than dimension-specific pathways. This approach is also consistent with the scoring procedures of the two scales, and the total scores of both scales showed good internal consistency in the present sample.

We considered the possibility of conducting exploratory dimension-level path analyses. However, simultaneously separating academic burnout and athlete burnout into multiple dimensions would substantially increase the number of model parameters and multiple comparisons, which may go beyond the core focus of the original theoretical model. Therefore, we decided to retain the total-score analytic strategy and added an explanation for this decision in Section 2.1 Instruments.

Q6.Due to the cross-sectional nature of the data, the serial mediation model implies a directional flow that cannot be definitively confirmed. Causal language (e.g., “how PSS influences AB,” “PSS alleviates AB”) should be tempered carefully throughout the abstract and discussion, explicitly framing the results as associative rather than causal.

Response:Thank you for this helpful suggestion. In response to the reviewer’s comment, we revised the Abstract and Discussion sections to avoid causal wording and to present the findings as associations. Specifically, we replaced expressions such as “influence” and “alleviate” with more cautious terms, such as “was associated with” and “was indirectly associated with.”

Q7. The path diagrams (Figures 1, 2, and 3) appear somewhat rudimentary and visually disjointed. Redesigning these flowcharts for improved aesthetic clarity and ensuring they meet PLOS ONE’s high-resolution figure guidelines will significantly improve readability. Finally, the discussion on practical interventions remains somewhat generic; please elaborate on how athletic departments and coaches can tangibly implement the suggested emotion regulation training in a dual-career student-athlete context.

Response:Thank you for this valuable comment. In response to the reviewer’s suggestion, we have redrawn the three figures to improve their clarity and readability. We have also expanded the Discussion section to further elaborate the practical implications for college athletes in the dual-career context.

Specifically, Lines 576–583 of the revised manuscript now indicate that, from a practical perspective, colleges, athletic departments, teachers, coaches, and counselors should combine CR training with problem-focused and context-focused support. These supports may include academic guidance, emotional support, ATHB monitoring, training-load adjustment, recovery management, instrumental academic support, and coordination of academic–athletic role demands.

These revisions make the practical recommendations more specific and better address the intervention needs of college athletes in the dual-career context.

Response to Reviewer 2

Dear Reviewer,

We sincerely thank you for your careful evaluation of our manuscript and for providing constructive and insightful comments. Your suggestions were highly valuable and helped us further improve the presentation of the study, the methodological description, and the interpretation of the findings. We greatly appreciate the time and effort you devoted to reviewing our work.

In response to your comments, we have carefully revised the manuscript and provided detailed point-by-point responses below. We believe that these revisions have further improved the clarity, rigor, and overall quality of the manuscript.

Sincerely,

The Authors

Q1. The authors acknowledge in Lines 206–209 that the dataset used in the present study was collected from the same cohort of college athletes as their previously published study (Reference 33: Xu et al., 2025, Frontiers in Psychology). The earlier study and the present manuscript appear to share several closely related elements: academic burnout serves as the dependent variable in both papers, athlete burnout is a key variable in both, and emotion regulation/cognitive reappraisal is involved in both analytical frameworks.

I do not suggest that the use of the same cohort across multiple studies is inherently inappropriate. However, when the same participant cohort and closely related psychological variables are used across separate manuscripts, the relationship between the studies should be disclosed with greater transparency. At present, the manuscript addresses this issue only briefly, stating that the research objectives, variable selection, and analytical framework are different. This statement is helpful, but it does not provide enough detail for readers to evaluate the degree of overlap and the distinct contribution of the present manuscript.

I recommend that the authors add a brief clarification in the Methods section, or in the supplementary materials if space is limited, explaining the relationship between the present study and the previously published paper. This clarification should specify which variables overlap across the two studies, whether the relevant measures were collected during the same survey administration, and what distinct theoretical or analytical contribution the present manuscript makes beyond the prior publication.

Response:Thank you for this professional and constructive suggestion. In the revised manuscript, we have explicitly clarified that the two studies used the same cross-sectional survey data from the same cohort of participants and included some overlapping variables. However, the two studies addressed different theoretical questions.

Specifically, our published article examined the burnout spillover process in which athlete burnout was associated with academic burnout through cognitive reappraisal and sleep quality. In contrast, the present study is grounded in Conservation of Resources theory and treats perceived social support as the predictor, psychological resilience and athlete burnout as sequential mediators, and cognitive reappraisal as a moderating emotion-regulation resource.

Therefore, the incremental contribution of the present study lies in shifting the research focus from the burnout spillover process to a resource-oriented explanation; that is, how perceived social support is associated with academic burnout through the sequential pathway of psychological resilience and athlete burnout, and how this process varies according to athletes’ levels of cognitive reappraisal.

We have also further explained why these variables were not integrated into one comprehensive model. The two studies were designed to answer different theoretical questions. Including perceived social support, psychological resilience, athlete burnout, cognitive reappraisal, sleep quality, and academic burnout in a single comprehensive model would substantially increase model complexity and weaken the theoretical focus of the specific mechanisms examined in each study. Therefore, we tested two theoretically distinct models with clear boundaries to maintain theoretical clarity and avoid overcomplicating the model. Please refer to Lines 177–188 and 423–428 in the revised manuscript.

Q2. Lines 258–268 describe the Sports Mental Toughness Questionnaire (SMTQ), including its origin, dimensional structure, scoring method, and internal reliability. However, in the correlation matrix and subsequent mediation and moderation models, the corresponding construct is labeled as psychological resilience (PR), rather than mental toughness. This creates conceptual and methodological ambiguity.

The authors should explicitly state whether the SMTQ total score was used as the operational indicator of PR. If so, this operational decision requires clear justification, because mental toughness and psychological resilience are related but not identical constructs. The Methods section should explain why a measure originally designed to assess sports mental toughness can validly represent psychological resilience in the present model. The Results section should also make the naming consistent, so that readers can clearly understand whether the construct being tested is mental toughness, psychological resilience, or a theoretically justified proxy for resilience.

At present, the manuscript appears to move from the measurement label “sports mental toughness” to the analytical label “psychological resilience” without sufficient explanation. This should be clarified before readers can properly evaluate the validity of the mediation model.

Response:Thank you for this important comment. We agree that the original manuscript did not clearly explain the transition between the measurement label “sport mental toughness” and the analytic label “psychological resilience (PR),” which may have caused conceptual and methodological ambiguity.

To clarify this issue, we have revised the Methods section to explicitly state that the total score of the SMTQ was used as an operational indicator of psychological resilience in the present study. We further clarified that psychological resilience in this manuscript should be understood as sport-specific psychological resilience, or resilience-related psychological resources in the sport context, rather than a direct measure of general psychological resilience. Specifically, the revised manuscript now states that all SMTQ item scores were summed to form a total score, and this total score was used to operationalize resilience-related psychological resources in the sport context, namely sport-specific PR.

In addition, we have revised the Results section, relevant tables, and model descriptions to improve the consistency of variable naming. The variable labeled as PR in the correlation matrix and subsequent mediation and moderation models refers to the operational indicator of sport-specific PR derived from the SMTQ total score, rather than a direct measure of general psychological resilience. Please refer to Lines 281–288 in the revised manuscript.

Q3.Related to the previous point, the manuscript repeatedly frames the relevant mediator as “psychological resilience,” citing literature that defines resilience as the capacity for recovery, adaptation, and sustained functioning under adversity. However, the actual measurement instrument appears to be the SMTQ, which was designed to assess sports mental toughness rather than resilience per se.

Although mental toughness and psychological resilience overlap in sport psychology, they should not be treated as fully interchangeable without theoretical or empirical justification. Resilience generally emphasizes recovery, adaptation, and positive functioning after adversity or stress exposure, whereas mental toughness more strongly emphasizes confidence, constancy, persistence, and perceived control under pressure. These conceptual differences matter because the manuscript interprets the mediator as an internal psychological resource that is built through perceived social support and then reduces athlete burnout and academic burnout.

I recognize that the data have already been collected and that it may not be feasible to re-administer an alternative resilience measure, such as the Connor-Davidson Resilience Scale, to the same 1,918 participants. Therefore, I do not require the authors to replace the measure. However, the authors should address this construct-validity issue in three specific ways.

First, in the Methods section, the authors should add a paragraph justifying the use of the SMTQ to operationalize psychological resilience. This justification should cite sport psychology literature showing the conceptual and empirical overlap between mental toughness and resilience, especially in athlete populations.

Second, in the Limitations section, the authors should explicitly acknowledge that the SMTQ was not originally designed to measure psychological resilience per se. The authors should explain that this operationalization choice may affect the interpretation of the PR-related findings and that future research using dedicated resilience measures would be necessary to verify the robustness of the current mediation results.

Third, the authors should provide additional evidence that the construct measured by the SMTQ is empirically distinguishable from the other constructs in the model. This is especially important given the relatively high correlations among some self-report variables, such as the correlation between PR and CR. A confirmatory factor analysis, or another appropriate test of discriminant validity, would help demonstrate that the SMTQ-based mediator captures a distinct construct rather than reflecting a broader general psychological adjustment factor.

Without these clarifications, the current manuscript risks overstating the evidence for a psychological resilience mechanism when the empirical measure may more accurately reflect sports mental toughness. This does not necessarily invalidate the findings, but it does require a more cautious and transparent interpretation.

Response:Thank you for this detailed and constructive suggestion. In response to the reviewer’s comment, we made revisions in the following three aspects.

First, in the Methods section, we added a clarification regarding the use of the SMTQ total score as an operational indicator of sport-specific PR. The revised manuscript now states that although the SMTQ was originally developed to measure sport mental toughness rather than psychological resilience in a general sense, the sport psychology literature suggests that mental toughness and psychological resilience share a closely related conceptual space, as both involve adaptive functioning under stressful conditions and the capacity to recover from adversity. In addition, previous athlete studies and systematic reviews have used or identified the SMTQ as a resilience-related measurement tool in competitive sport contexts. Therefore, in the present athlete sample, using the SMTQ total score as an operational indicator of sport-specific PR has a theoretical basis. Please refer to Lines 281–288 in the revised manuscript.

Second, in the Limitations section, we have explicitly acknowledged that the SMTQ was not originally designed to measure general psychological resilience. Therefore, using the SMTQ total score to represent PR may affect the interpretive boundaries of PR-related findings. We further noted that future studies should use dedicated psychological resilience measures, such as the Connor-Davidson Resilience Scale, or compare different measures of psychological resilience and sport mental toughness to verify the robustness of the current mediation results. Please refer to Lines 608–613 in the revised manuscript.

Third, we agree that it is necessary to further examine the empirical distinction between the psychological resilience construct operationalized by the SMTQ and other constructs in the model, especially cognitive reappraisal. To this end, we added CFA model comparisons and discriminant validity tests in the Results section. The results showed that the hypothesized five-factor CFA model, including perceived social support, psychological resilience, athlete burnout, cognitive reappraisal and academic burnout had an acceptable fit and performed better than the alternative models: χ² = 1127.001, df = 125, CFI = 0.952, TLI = 0.941, RMSEA = 0.065, SRMR = 0.050. Further discriminant validity tests showed that the AVE values of all constructs were above 0.50, and the square root of each construct’s AVE was greater than its correlations with other constructs. In addition, the HTMT values ranged from 0.379 to 0.727, all below the recommended threshold of 0.85. These results suggest that, although psychological resilience was correlated with variables such as cognitive reappraisal , the construct measured by the SMTQ could still be empirically distinguished from cognitive reappraisal and other constructs, and was unlikely to reflect a broader general psychological adaptation factor. Please refer to Lines 349–355 in the revised manuscript and Tables S1–S3.

Q4. The study employs five self-report instruments simultaneously, with notably high inter-correlations among several variables. For example, Table 2 reports relatively high correlations between PSS and CR (r = 0.658) and between PR and CR (r = 0.560). Under these circumstances, it would be important to provide CFA results, or other appropriate evidence of discriminant validity, to confirm that the five constructs are empirically distinguishable.

At present, the manuscript reports internal consistency coefficients and bivariate correlations, but it does not provide sufficient evidence that the measured constructs are distinct from one another. The particularly high correlation between PSS and CR raises the question of whether these two variables may partly reflect overlapping aspects of a broader positive psychological adjustment factor. I recommend that the authors report additional evidence of construct distinctiveness, such as confirmatory factor analysis, comparison of alternative factor models, AVE-based discriminant validity, HTMT, or another appropriate approach.

Response:Thank you for this helpful suggestion. In response to the reviewer’s comment, we supplemented the revised manuscript with CFA model comparisons, AVE-based discriminant validity tests, and HTMT analyses.

The results showed that the five-factor CFA model, including perceived social support, athlete burnout, psychological resilience, cognitive reappraisal, and academic burnout as five latent variables, had an acceptable fit and performed better than the alternative models (χ² = 1127.001, df = 125, CFI = 0.952, TLI = 0.941, RMSEA = 0.065, SRMR = 0.050). Further discriminant validity analyses showed that the AVE values of all constructs were above 0.50, and the square root of each construct’s AVE was greater than its correlations with other constructs. In addition, the HTMT values ranged from 0.379 to 0.727, all below the recommended threshold of 0.85. Notably, the HTMT values for PSS–CR and PR–CR were 0.727 and 0.689, respectively, indicating acceptable discriminant validity even between relatively highly correlated constructs.

These revisions can be found in Sections 3.1 and 3.2 of the revised manuscript, and the full results are reported in Supplementary Tables S1–S3.

Q5. Table 3 reports the 95% confidence interval for the PSS → ATHB → AB indirect effect as [−0.065, −0.028], whereas Line 312 in the text reports the same result as [−0.064, −0.027]. Both the lower and upper bounds differ. Although the discrepancy is numerically small, reporting different values for the same result within the same manuscript undermines the credibility of the data. The authors should verify the original PROCESS output and ensure consistency throughout.

Response:Thank you for pointing this out. We rechecked the original PROCESS output and carefully reviewed all indirect effects and their bootstrap confidence intervals reported in the text and Table 3. The previous inconsistency was mainly due to rounding and an oversight during the reporting process.

In the revised manuscript, we have standardized and corrected the relevant values throughout the text. Specifically, the indirect effect of PSS on AB through ATHB is now consistently reported in both the main text and Table 3 as Effect = −0.045, 95% CI [−0.065, −0.027]. We also checked the other indirect effects, the total indirect effect, and the total effect to ensure consistency among the main text, tables, and the PROCESS output.

Q6. Figure 2 presents standardized path coefficients from the serial mediation model (Model 6), such as PR → AB = −0.150 and ATHB → AB = 0.327. These values differ from those in Table 4, which reports Model 89 coefficients (−0.108 and 0.302, respectively). I understand this discrepancy arises because the two models include different sets of predictors, which is expected. However, the authors never provide the full regression coefficient table for Model 6. Only the indirect effects appear in Table 3, leaving the path coefficients shown in Figure 2 unverifiable against any tabulated data. I recommend adding a complete Model 6 regression table in the main text or supplementary materials.

Response:Thank you for this helpful suggestion. In response to this comment, we further included sport item and sport level as additional covariates on the basis of the original control variables and re-estimated PROCESS Model 6. In the revised manuscript, we have added Table S4 to the Supplementary Materials, which reports the full regression results of Model 6, including regression coefficients, standard errors, p values, 95% confidence intervals, control variables, and model statistics. This allows the main path coefficients presented in Figure 2 to be directly verified.

In addition, Figure 2 presents the path coefficients of the serial mediation model estimated using PROCESS Model 6, whereas Table 4 presents the coefficients of the moderated serial mediation model estimated using PROCESS Model 89. Because Model 89 additionally included cognitive reappraisal and the interaction terms PSS × CR, PR × CR, and ATHB × CR in the equation predicting academic burnout, the path coefficients for PR → AB and ATHB → AB were not expected to be identical to the corresponding coefficients in Model 6. Thus, the observed differences reflect the different sets of predictors included in the two model specifications, rather than inconsistencies in the statistical results.

Please refer to Table S4 in the Supplementary Materials.

Q7. Table 4 reports the p-value for the PSS × CR interaction term as p = 0.010, while Line 327 describes the same result as “p < 0.05.” The manuscript alternates between exact p-values and inequality notation throughout. I recommend adopting a uniform reporting standard.

Response:Thank you for pointing this out. After further controlling for sport item and sport level in addition to the original covariates, we reran PROCESS Model 89. The updated results showed that the interaction term PSS × CR significantly predicted AB (β = −0.052, p = 0.009). Therefore, we have revised this result consistently in Table 4 and the Results section as p = 0.009, instead of using general expressions such as “p < 0.05” or “p < 0.01.” We have also standardized the reporting format of other p values throughout the manuscript.

Q8. When the correlation between an independent variable and a moderator reaches 0.66, interaction term estimates may become unstable. The authors note in Lines 283–284 that variables were mean-centered prior to computing interaction terms, which is appropriate. However, mean-centering only reduces collinearity between the interaction term and the constituent main effects; it does not address collinearity between the main effects themselves. I recommend reporting VIF (Variance Inflation Factor) values for each predictor across all models.

Response: Thank you for this helpful comment. We conducted collinearity diagnostics for the predictors and all covariates included in the regression models. The results showed that the VIF values of all predictors ranged from 1.010 to 2.014, which were all below the commonly used threshold of 5. The Tolerance values ranged from 0.496 to 0.990, which were all above 0.10. These results indicate that multicollinearity was unlikely to seriously affect the model estimates.

We have added these results to Section 3.2, “Correlation Analysis and Discriminant Validity Assessment,” in the revised manuscript.

Q9. Lines 287–294 report that the first unrotated factor explained 23.769% of the variance, below the 40% threshold. However, Harman’s test has been widely criticized in the methodological literature for its low sensitivity and limited statistical power. I recommend that the authors at minimum acknowledge this limitation, and ideally supplement the test with a more robust approach, such as adding a common method factor within the CFA framework.

Response:Thank you for this important suggestion. We agree that Harman’s single-factor test has relatively low sensitivity and should not be used as the sole basis for assessing common method bias. Therefore, in Section 3.1, “Common Method Bias,” of the revised manuscript, we retained Harman’s test as a preliminary diagnostic and further supplemented it with a CFA-based unmeasured latent method construct (ULMC) analysis.

Specifically, we first established a five-factor CFA model. We then added an unmeasured latent method construct to the five-factor CFA model to examine the potential influence of common method bias. The results showed that the five-factor CFA model had an acceptable fit (χ² = 1127.001, df = 125, CFI = 0.952, TLI = 0.941, RMSEA = 0.065). Subsequently, we added an unmeasured latent method construct to this model and constrained its loadings on all observed indicators to be equal. The results showed that the ULMC model did not lead to a substantial improvement in model fit (χ² = 1127.001, df = 124, CFI = 0.952, TLI = 0.940, RMSEA = 0.065, SRMR = 0.050). These results suggest that common method bias was unlikely to substantially affect the main findings of this study.

Q10. The moderated serial mediation model tested in the manuscript specifies CR as a moderator of paths leading to AB, including the PSS → AB, PR → AB, and ATHB → AB paths. This specification is theoretically plausible, but the manuscript does not sufficiently justify why CR was modeled only at these later-stage paths rather than at earlier stages of the serial chain, such as PSS → PR, PSS → ATHB, or PR → ATHB.

Given that the Introduction describes CR as a factor that may influence the transformation of external support into internal psychological resources, one might also expect CR to moderate earlier resource-transformation paths. I recommend that the authors provide a clearer theoretical rationale for the chosen PROCESS Model 89 specification and acknowledge that alternative moderated mediation models could test different boundary conditions within the serial pathway.

Response:Thank you for this constructive comment. We agree that the original wording in the Introduction may have led readers to infer that CR could also moderate earlier resource-transformation pathways. To avoid this ambiguity, we revised the Introduction to further clarify the theoretical rationale for the specification of PROCESS Model 89.

Specifically, drawing on the process model of emotion regulation and COR theory, we now conceptualize CR as an outcome-proximal emotion-regulation resource in relation to AB. Because CR primarily involves the reinterpretation of stressful situations and emotional experiences, it is more directly relevant to how college athletes appraise perceived support, internal psychological resources, and burnout-related experiences in relation to academic burnout. Therefore, in the present study, CR was specified as a moderator of the direct paths leading to AB, namely PSS → AB, PR → AB, and ATHB → AB, rather than as a moderator of earlier resource-accumulation paths such as PSS → PR, PSS → ATHB, or PR → ATHB.

We have also added a note in the Discussion/Limitations section acknowledging that alternative moderated mediation models may also be theoretically plausible. Future longitudinal studies should compare models with different moderator positions within the sequential pathway.

Please refer to Lines 166–170 and the Limitations section in the revised manuscript.

Q11. Table 4 and Figure 5 show that CR positively moderates the ATHB → AB path (β = 0.078, p < 0.001). The simple slope analysis indicates that the association between ATHB and AB is stronger among athletes with higher CR (β = 0.380) than among those with lower CR (β = 0.223). In other words, higher CR is associated with a stronger ATHB–AB link, rather than a weaker one.

This finding complicates, and partly contradicts, the manuscript’s general framing of CR as a uniformly protective factor. Although CR appears to strengthen the protective association between PSS and AB, its moderating role in the ATHB → AB path operates in the opposite direction from what a simple protective-factor interpretation would predict.

The authors briefly touch on this result in Lines 476–479 but do not meaningfully engage with its theoretical implications. I believe this finding warrants a more thorough discussion. Possible explanations might include: (a) cognitive reappraisal may be effective under mild stress but may fail or even backfire when athletic burnout reaches severe levels, with high-CR individuals becoming more acutely aware of their deteriorating state; (b) high-CR individuals may over-rely on reappraisal strategies while neglecting problem-focused coping, leading to strategy exhaustion when burnout accumulates; or (c) there may be blurred boundaries between reappraisal and rumination, such that some individuals’ “reappraisal” effectively constitutes repeated cognitive processing of negative experiences. These possibilities deserve serious discussion rather than a cursory mention.

In its current form, the Discussion risks overstating the protective role of CR and underinterpreting a theoretically important, counterintuitive moderation effect.

Response:Thank you for this important comment. We agree that the positive moderating effect of CR on the ATHB → AB path complicates the interpretation of CR as a uniformly protective factor. Specifically, the revised Results section now explicitly states that the ATHB × CR interaction term was significant and positive (β = 0.078, p < 0.001), indicating that CR strengthened the ATHB → AB path. The simple slope analysis showed that ATHB had a stronger predictive effect on AB at higher levels of CR (β = 0.381, p < 0.001), whereas this effect was relatively weaker at lower levels of CR (β = 0.224, p < 0.001).

In the revised Discussion section, we have explicitly acknowledged that the simple slope analysis showed a stronger ATHB–AB association among individuals with higher CR. Accordingly, we removed statements that implied that CR weakened the ATHB–AB association and revised the interpretation to emphasize the path-specific and context-dependent role of CR. Specifically, we added several possible explanations for this counterintuitive finding. First, CR may be adaptive under mild or moderate stress, but when burnout-related strain becomes severe, repeated cognitive reframing may become less effective or even cognitively costly. Second, individuals with higher CR may over-rely on cognitive reframing while engaging less in problem-focused coping strategies, which may contribute to strategy exhaustion when ATHB continues to accumulate. Third, under chronic burnout conditions, the boundary between adaptive reappraisal and rumination may become blurred; for some athletes, self-reported cognitive reappraisal may partly reflect repeated cognitive processing of burnout-related difficulties, which may help explain the stronger ATHB–AB association at higher levels of CR.

Please refer to Lines 537–560 in the revised manuscript.

Q12. The simple slope analysis for the PSS × CR interaction suggests that, among athletes with lower CR, the protective association between PSS and AB is weaker and does not reach conventional statistical significance (effect = −0.058, p = 0.066). By contrast, the association is stronger and statistically significant among athletes with higher CR (effect = −0.162, p < 0.001).

This pattern has important implications for intervention design. It suggests that enhancing social support alone may be less effective for athletes who have lower levels of cognitive reappraisal, and that social support interventions may need to be combined with emotion regulation training to produce more consistent benefits for reducing academic burnout.

However, the manuscript’s discussion of this point is too brief. I recommend that the authors elaborate on the practical implications of this conditional effect, especially with regard to how universities, coaches, counselors, and academic support staff might design interventions that integrate social support enhancement with emotion regulation training.

Response: Thank you for this helpful suggestion. We agree that the conditional effect of the PSS × CR interaction has important implications for intervention design. In the revised analysis, the simple slope estimates changed slightly, but the substantive interpretation remained consistent. Specifically, at higher levels of CR, PSS predicted lower AB (M + 1 SD; effect = −0.162, p < 0.001), whereas at lower levels of CR, this association was weaker and did not reach statistical significance (M − 1 SD; effect = −0.057, p = 0.072). Please refer to Lines 381–384 in the revised manuscript.

In response to the reviewer’s comment, we expanded the Discussion section to clarify the practical implications of this conditional effect. We now further explain that, for athletes with lower CR, simply increasing social support may be insufficient because they may have more difficulty cognitively reframing perceived support as a positive resource for coping with academic stress. Therefore, interventions targeting AB among college athletes should combine social support enhancement with CR training. Please refer to Lines 576–585 in the revised manuscript.

Q13. The AB equation in Table 4 explains 25.9% of the variance in academic burnout. While this represents a meaningful proportion of explained variance in psychological research, the manuscript does not report individual predictor effect sizes or discuss the practical magnitude of the observed associations. I recommend that the authors supplement the statistical significance tests with effect size indicators where appropriate, such as semi-partial effects, standardized effect interpretation, or f² values. More importantly, the Discussion should clarify what the observed effects mean for practical intervention design. For example, the authors should explain whether the effects of PSS, ATHB, and CR are sufficiently large to support targeted intervention programs, or whether the findings should be interpreted as preliminary evidence requiring further validation.

Response: Thank you for this constructive suggestion. In the revised Results section, we have supplemented the manuscript with effect size indices. According to the updated analysis, the academic burnout equation explained 26.2% of the variance, R² = 0.262, F(13, 1904) = 51.862, p < .001, corresponding to Cohen’s f² = 0.355, which indicates a relatively strong overall explanatory effect. Please refer to Lines 377–380 in the revised manuscript.

In addition, we further calculated the local effect sizes (local f²) of the core predictors in the academic burnout equation to reflect the unique contribution of each predictor after controlling for the other variables. The results showed that athlete burnout had the largest local effect size (local f² = 0.092), whereas the local effect sizes of perceived social support, psychological resilience, cognitive reappraisal, and the interaction terms were relatively small, ranging from 0.000 to 0.011. These effect size indices have been added to Table 4.

We also further clarified the practical implications of these effects in the Discussion section. The revised manuscript now indicates that ATHB had a relatively stronger unique association with AB and therefore may be a priority intervention target for reducing academic burnout among college athletes. In contrast, the smaller local effects of PSS, PR, CR, and the interaction terms suggest that social support enhancement, resilience-related psychological resource building, and CR training may be more appropriate as complementary components of a multicomponent intervention, rather than as stand-alone solutions.

Q14. The authors successively draw upon the stress-buffering model (Line 91), Conservation of Resources theory (Line 106), the process model of emotion regulation (Line 151), and later in the Discussion introduce the Job Demands–Resources model (Line 448) and self-regulation theory (Line 470). While each framework has some relevance, the current presentation lacks a unifying logical thread and reads more like a list of theories than an integrated framework. I recommend anchoring the paper in COR theory as the primary framework and clearly positioning the other theories in supporting roles.

Response:Thank you for this insightful suggestion. In response to the reviewer’s comment, we revised the Introduction and Discussion sections to position Conservation of Resources (COR) theory as the core theoretical framework of the present study, while treating other theories only as supplementary perspectives.

Specifically, COR theory is now used as the overarching framework to explain the processes of resource gain, resource protection, and resource loss among college athletes facing dual academic–athletic demands . The stress-buffering model is now used only as a supplementary perspective to explain the role of social support as an external resource, whereas the Process Model of Emotion Regulation is used to support CR as a boundary condition that may influence how athletes interpret and use available resources. We have also removed references to the Job Demands–Resources model and self-regulation theory as independent explanatory frameworks in the Discussion section to avoid theoretical fragmentation(Lines 171–188).

Q15. The proposed sequence assumes that social support builds resilience, which in turn reduces athletic burnout, and the reduction in athletic burnout then lowers academic burnout. However, under a cross-sectional design, alternative causal orderings are equally plausible. For example, ATHB → PR → AB (athletic burnout erodes resilience, which then exacerbates academic burnout) is theoretically defensible. I recommend that the authors provide more thorough theoretical justification for the chosen ordering in the Introduction, and indicate whether any competing models were tested. If not, this should be acknowledged in the Limitations section.

Response:Thank you for this important comment. We agree that, given the cross-sectional design, the proposed sequential pathway cannot be interpreted as a strict causal order, and other directional pathways are also theoretically possible. Therefore, we have further softened the causal wording throughout the revised manuscript and added a clearer theoretical rationale in the Introduction for specifying the PSS → PR → ATHB → AB sequence.

Specifically, perceived social support was placed at the beginning of the model because social support is generally regarded as a relatively external psychosocial resource that can provide emotional, informational, and instrumental support, thereby promoting adaptive psychological resources under stressful conditions. PR was positioned before athlete burnout because it reflects individuals’ adaptive and regulatory capacity in response to stress and challenges, which may theoretically buffer the impact of training, competition, and academic demands on burnout experiences. ATHB was placed before AB based on the dual academic–athletic role context of college athletes: persistent exhaustion in the athletic domain may occupy psychological and behavioral resources and become associated with burnout in the academic domain. Accordingly, the present study examined PSS → PR → ATHB → AB as a theory-driven sequential pathway based on Conservation of Resources theory and relevant prior evidence.

At the same time, we have explicitly acknowledged in the Limitations section that this study did not systematically test competing sequential models. Because the study used a cross-sectional self-report questionnaire design, it cannot determine the temporal order or causal direction among the variables, nor can it rule out alternative pathways, such as ATHB → PR → AB. Future research should use longitudinal, cross-lagged, or experimental/intervention designs to further compare the plausibility and robustness of different competing pathways.

Please refer to the revised Introduction and Limitations sections.

Q16. H7 reads “CR moderates the relationship between ABTH and AB.” The abbreviation should be “ATHB,” not “ABTH.” Please also check the x-axis label of Figure 5 and all related figure labels for abbreviation consistency, and correct this issue throughout the manuscript.

Response: Thank you for pointing out this inconsistency in the abbreviation. We have corrected “ABTH” to “ATHB” in the statement of H7. We also carefully checked the x-axis label, title, and note of Figure 5, as well as the table labels and all related descriptions throughout the manuscript. In the revised manuscript, “ATHB” is now used consistently to refer to athlete burnout.

Q17. Lines 386–389 discuss how PSS may modulate the sympathetic nervous system and the HPA axis to reduce physiological stress responses. However, the present study collected no physiological data whatsoever. While citing relevant literature as theoretical background is acceptable, the current phrasing risks misleading readers into thinking these mechanisms are findings of the present study. I recommend adjusting the wording to clearly mark these as speculative interpretations based on prior literature.

Response:Thank you for this important comment. To avoid potential misunderstanding, we have removed the sentences discussing the regulation of the sympathetic nervous system and the HPA axis by PSS. The revised Discussion is mainly grounded in COR theory and interprets PSS as an external social resource, focusing on its association with lower AB. This revision avoids overinterpretation and ensures that the discussion remains consistent with the variables actually measured in the present study.

Q18. The Limitations discussion (Lines 496-509) covers cross-sectional design, self-report instruments, and the non-significant PR × CR interaction, which is reasonable. However, it omits several points worth discussing: (a) the sample was recruited through convenience sampling from Chinese university sports teams, and the representativeness of the sample may be limited, particularly given the uneven distribution of sport types and athlete levels; (b) using the SMTQ to operationalize psychological resilience may introduce construct validity concerns (see Q3); and (c) the high collinearity between PSS and CR (r = 0.658) may affect the stability of interaction term estimates (see Q8).

Response:Thank you for this helpful suggestion. We have revised this section accordingly.

First, we now acknowledge that the participants in this study were recruited through convenience sampling from Chinese university sports teams, which may limit the generalizability of the findings. Therefore, future research should use more diverse and representative samples and examine whether the model remains stable across different sports, competitive levels, and cultural contexts.

Second, we have added a limitation regarding the use of the SMTQ to operationalize PR. Although the SMTQ can reflect resilience-related characteristics in the sport context, it was originally developed to measure sport mental toughness rather than general psychological resilience. Therefore, using the SMTQ total score as an operational indicator of PR may raise certain construct-validity concerns and affect the interpretive boundaries of PR-related findings. We now suggest that future studies should use dedicated psychological resilience measures or compare multiple measurement tools to further verify the robustness of the findings.

Third, we have expanded the discussion of collinearity between PSS and CR. We agree that the relatively high correlation between PSS and CR may affect the stability of the interaction-term estimates. In response to Q8, we conducted additional collinearity diagnostics. The results showed that the VIF values of all predictors ranged from 1.286 to 1.745, and the tolerance values ranged from 0.573 to 0.777, suggesting that severe multicollinearity was unlikely. Nevertheless, we still acknowledge in the Limitations section that the relatively high PSS–CR correlation should be considered when interpreting the interaction effects, and that future studies should further replicate these moderation effects in independent samples.

Please refer to the Limitations section in the revised manuscript.

Q19. The reference list uses journal abbreviations for some entries and full journal names for others; some entries include complete page information while others provide only article numbers; and the formatting of translated Chinese-language references is inconsistent with other entries. Please revise in accordance with PLOS ONE reference formatting requirements.

Response:Thank you for this helpful comment. We have carefully revised the reference list according to the PLOS ONE reference formatting requirements. Journal title abbreviations, page ranges or article numbers, DOI information, and translated Chinese-language references have been standardized throughout the reference list. We also checked the numbering of all references to ensure consistency with the in-text citations.

Q20. After a careful reading of the Introduction, I find that the empirical grounding for the hypotheses is uneven. H1 and H2 are reasonably well supported by cited literature. H3, however, relies on the ATHB → AB spillover assumption, for which the only cited evidence is the authors’ own prior work (Reference 33, Line 142: “our preliminary work suggests”). A single self-citation is insufficient to anchor a mediation hypothesis; the authors should supplement this with independent evidence on cross-domain burnout transfer.

H4, the core serial mediation hypothesis, lacks direct empirical precedent. No cited study has tested a sequential PSS → PR → ATHB → AB pathway, and the critical PR → ATHB link receives only a brief mention at Lines 136–138 (Reference 29), citing a correlational association rather than a mediation relationship. A four-variable serial mediation hypothesis requires stronger literature support than what is currently provided.

Most critically, the moderation hypotheses H5 through H8 (Lines 176–179) suffer from a systematic logical gap. The authors’ argument in Lines 154–161 proceeds as follows: CR is related to AB (Reference 36), CR is related to resilience (Reference 37), and CR is related to ATHB (Reference 38). From these bivariate associations, the authors derive hypotheses that CR moderates specific paths (PSS → AB, PR → AB, ATHB → AB, and the serial indirect effect). However, "CR correlates with variable X" does not logically entail "CR moderates the path from X to Y." Correlation and moderation are distinct statistical and conceptual relationships. Furthermore, Reference 36 actually examines CR as a mediator between social support and academic burnout, not as a moderator. Using a mediation finding to justify a moderation hypothesis conflates two different theoretical mechanisms. I recommend that the authors either locate prior studies that have specifically tested CR (or related emotion regulation strategies) as a moderator of burnout pathways, or reframe the moderation hypotheses as more exploratory in nature and adjust the language in the Introduction accordingly.

Response:Thank you for this important and constructive comment. We agree that the original Introduction did not provide sufficiently balanced empirical support for H3, H4, and H5–H8. Accordingly, we have systematically revised the Introduction.

First, to strengthen the theoretical and empirical basis for H3, we added independent evidence regarding the relationship between burnout in the sport domain and burnout in the school/academic domain. Specifically, the revised manuscript cites research on the dual career of student-athletes, showing a strong positive correlation between school burnout and sport burnout. This suggests that burnout experiences in the academic and athletic domains are not independent of each other among student-athletes. Meanwhile, we now use previous evidence showing a significant association between ATHB and AB among college athletes as supplementary evidence, rather than as the sole basis for H3. Together with findings showing that higher perceived social support is associated with lower levels of ATHB, these studies provide stronger support for conceptualizing ATHB as a potential mediator between PSS and AB.

Second, regarding H4, we now explicitly acknowledge in the revised manuscript that, to the best of our knowledge, no prior study has directly examined the complete PSS → PR → ATHB → AB sequential pathway. Therefore, we have reframed H4 as a theory-driven sequential pathway based on COR theory and evidence from interconnected paths, rather than as a direct extension of an existing four-variable model. We added evidence showing that psychological resilience is associated with lower levels of ATHB, as well as athlete-related research that conceptualizes mental toughness as a resilience-related construct in the sport context and shows that it can mediate the relationship between social support and athlete burnout. Taken together, these findings provide empirical support for integrating the interconnected pathways among PSS, PR, ATHB, and AB. Based on this, the revised manuscript presents H4 as a theory-driven sequential pathway examining the potential links among external social resources, internal psychological resources, athletic-domain exhaustion, and academic-domain exhaustion under the dual academic–athletic demands faced by college athletes.

Third, we rewrote the theoretical rationale for H5–H8 to avoid conflating correlational, mediating, and moderating mechanisms. In the revised manuscript, we removed statements that directly inferred moderation hypotheses from bivariate associations between CR and AB, PR, or ATHB. Instead, we now justify the moderation hypotheses based on the Process Model of Emotion Regulation and empirical studies in which CR or related emotion-regulation strategies functioned as moderators in burnout-related pathways. For example, previous studies have shown that CR can moderate the association between intrusive rumination and academic burnout, as well as the relationships between stress and job burnout and between depression and job burnout. At the same time, we now explicitly acknowledge that direct evidence on whether CR moderates the specific pathways among PSS, PR, ATHB, and AB in college athletes remains limited. Therefore, CR is now presented as a theory-driven exploratory boundary condition in the revised manuscript.

Finally, in response to the reviewer’s question about why CR was placed on the later-stage paths, we further clarified that CR was conceptualized as a proximal emotion-regulation resource that mainly concerns how athletes reinterpret stressful situations, emotional experiences, and burnout-related appraisals. Therefore, the revised manuscript now explicitly states that the present study focused on whether CR moderated the direct paths leading to AB, namely PSS → AB, PR → AB, and ATHB → AB.

Please refer to Lines 136–152 and 159–170 in the revised manuscript.

Attachment Submitted filename: response letter for review comments.docx

# Peer review: Decision Letter 1

**DOI:** 10.1371/journal.pone.0361222.r003

**Authors:** Rogis Baker

16 Jul 2026

Dear Dr. Xu,

Thank you for submitting your manuscript to PLOS ONE. After careful consideration, we feel that it has merit but does not fully meet PLOS ONE’s publication criteria as it currently stands. Therefore, we invite you to submit a revised version of the manuscript that addresses the points raised during the review process.

Please submit your revised manuscript by Aug 30 2026 11:59PM. If you will need more time than this to complete your revisions, please reply to this message or contact the journal office at plosone@plos.org. When you're ready to submit your revision, log on to https://www.editorialmanager.com/pone/ and select the 'Submissions Needing Revision' folder to locate your manuscript file.

- A letter that responds to each point raised by the academic editor and reviewer(s). You should upload this letter as a separate file labeled 'Response to Reviewers'.
- A marked-up copy of your manuscript that highlights changes made to the original version. You should upload this as a separate file labeled 'Revised Manuscript with Track Changes'.
- An unmarked version of your revised paper without tracked changes. You should upload this as a separate file labeled 'Manuscript'.

If you would like to make changes to your financial disclosure, please include your updated statement in your cover letter. Guidelines for resubmitting your figure files are available below the reviewer comments at the end of this letter.

If applicable, we recommend that you deposit your laboratory protocols in protocols.io to enhance the reproducibility of your results. Protocols.io assigns your protocol its own identifier (DOI) so that it can be cited independently in the future. For instructions see: https://journals.plos.org/plosone/s/submission-guidelines#loc-laboratory-protocols. Additionally, PLOS ONE offers an option for publishing peer-reviewed Lab Protocol articles, which describe protocols hosted on protocols.io. Read more information on sharing protocols at https://plos.org/protocols?utm_medium=editorial-email&utm_source=authorletters&utm_campaign=protocols.

As the corresponding author, your ORCID iD is verified in the submission system and will appear in the published article. PLOS supports the use of ORCID, and we encourage all coauthors to register for an ORCID iD and use it as well. Please encourage your coauthors to verify their ORCID iD within the submission system before final acceptance, as unverified ORCID iDs will not appear in the published article. *Only*  the individual author can complete the verification step; PLOS staff *cannot*  verify ORCID iDs on behalf of authors.

We look forward to receiving your revised manuscript.

Kind regards,

Rogis Baker, Ph.D

Academic Editor

PLOS One

Journal Requirements:

If the reviewer comments include a recommendation to cite specific previously published works, please review and evaluate these publications to determine whether they are relevant and should be cited. There is no requirement to cite these works unless the editor has indicated otherwise.

[Note: HTML markup is below. Please do not edit.]

Reviewers' comments:

Reviewer's Responses to Questions

**Comments to the Author**

Reviewer #1: All comments have been addressed

Reviewer #2: (No Response)

**********

2. Is the manuscript technically sound, and do the data support the conclusions?

Reviewer #1: Yes

Reviewer #2: Partly

**********

3. Has the statistical analysis been performed appropriately and rigorously?

Reviewer #1: Yes

Reviewer #2: No

**********

4. Have the authors made all data underlying the findings in their manuscript fully available?

The PLOS Data policy

Reviewer #1: Yes

Reviewer #2: Yes

**********

5. Is the manuscript presented in an intelligible fashion and written in standard English?

Reviewer #1: Yes

Reviewer #2: Yes

**********

Reviewer #1: The authors have adequately addressed the concerns raised in the previous review and provided clear, detailed responses. The revised manuscript presents the research on academic burnout among college athletes logically and rigorously. Therefore, I recommend this manuscript for acceptance.

Reviewer #2: Manuscript ID: PONE-D-26-07482R1

Recommendation: Major Revision

Overall Assessment of the Revision

I appreciate the authors’ careful and detailed responses to my previous comments. The revised manuscript has improved substantially in several respects. The relationship between the present study and the authors’ previously published same-cohort paper is now disclosed more explicitly; the authors have clarified that the SMTQ total score was used as an operational indicator of a sport-specific resilience-related resource; CFA, AVE, HTMT, and common-method-factor analyses have been added; the numerical inconsistency in the indirect-effect confidence interval has been corrected; exact p-value reporting has been standardized; a fuller regression table for PROCESS Model 6 has reportedly been added to the Supplementary Materials; and the theoretical rationale for positioning cognitive reappraisal on the outcome-proximal paths has been strengthened. The revised Discussion also engages more seriously with the counterintuitive finding that cognitive reappraisal strengthens the positive association between athlete burnout and academic burnout. These revisions represent meaningful progress.

Several of my previous comments can therefore be considered satisfactorily addressed. In particular, the authors have corrected the discrepancy between the text and Table 3, standardized the reporting of the PSS × CR interaction, added effect-size information, revised the theoretical organization of the manuscript, corrected the ATHB abbreviation, removed the unsupported physiological explanation, and expanded the limitations section. I do not raise these matters again below.

However, several issues remain only partially resolved. The principal concerns relate to the scoring and interpretation of the SMTQ-based mediator, the specification and reporting of the newly added CFA and unmeasured latent method construct analyses, and the representation of cognitive reappraisal in the Abstract and Conclusion. In addition, the revised analysis raises a new question concerning the coding of multicategory covariates. These issues require further clarification and, depending on the authors’ actual coding and scoring procedures, may require reanalysis.

Major Comments

Major Comment 1: The authors have responded constructively to my previous concerns about the transition from the measurement label “sport mental toughness” to the analytical label “psychological resilience.” In the revised Methods section, around Lines 275–282, the authors now state explicitly that the SMTQ total score was used to operationalize a sport-specific resilience-related psychological resource rather than general psychological resilience. The Limitations section also acknowledges that the SMTQ was not originally designed as a dedicated measure of psychological resilience. These additions improve the transparency of the manuscript.

Nevertheless, the underlying construct-validity issue remains only partially resolved. Sport mental toughness and psychological resilience occupy related conceptual territory, but they are not fully interchangeable. The SMTQ assesses confidence, constancy, and control under pressure, whereas resilience more specifically concerns adaptation, recovery, and sustained functioning following adversity. The newly added CFA, AVE, and HTMT analyses can demonstrate that the SMTQ-based factor is empirically distinguishable from PSS, CR, ATHB, and AB, but they cannot establish that the SMTQ is a direct measure of psychological resilience. Discriminant validity from other variables is not equivalent to content or convergent validity as resilience. The manuscript should therefore avoid presenting the mediator unqualifiedly as psychological resilience. The authors may either relabel the construct as “sport mental toughness” or consistently describe it as an “SMTQ-based resilience-related psychological resource in the sport context,” but the terminology and interpretation must be aligned throughout the Introduction, hypotheses, figures, tables, Results, Discussion, and Conclusion. Claims that the study directly demonstrates a psychological-resilience mechanism should be narrowed accordingly.

A related scoring issue also requires clarification. The original manuscript stated that SMTQ Items 2, 4, 7, 8, 9, and 10 were reverse-coded, whereas the revised description appears to omit Item 10. The original scoring description is located in the SMTQ subsection, Lines 258–268. The authors should verify the scoring key actually used to compute the analytic variable and state clearly which description is correct. If the dataset was scored correctly and the inconsistency is only textual, an explicit confirmation and correction would be sufficient. If the scoring computation itself differed, the descriptive statistics, reliability analysis, CFA results, correlations, and PROCESS models involving this mediator would need to be recalculated.

Major Comment 2:The authors have undertaken the additional psychometric analyses requested in the previous review. The revised manuscript reports that the hypothesized five-factor model achieved acceptable fit and outperformed alternative models, and that AVE and HTMT results supported discriminant validity. The authors also added an unmeasured latent method construct model to supplement Harman’s single-factor test. These additions are directionally appropriate and represent a substantive response to my earlier comments.

However, the manuscript does not explain how the observed indicators in the CFA were constructed. The five measures contain a large number of individual items, yet the reported five-factor model has only 125 degrees of freedom. This suggests that the authors may have used subscale scores, item parcels, or another reduced-indicator approach, but the relevant procedure is not described. The authors should state clearly whether the CFA was conducted using individual items, scale dimensions, or parcels. If parcels were used, the parcel-construction procedure and its rationale should be reported, particularly because both academic burnout and athlete burnout are multidimensional constructs.

The reported baseline CFA and ULMC models also have exactly the same chi-square value, χ2=1127.001, despite differing by one degree of freedom. This is not necessarily impossible, but it is sufficiently unusual to require verification. The authors should confirm that the values were not duplicated inadvertently and briefly report the specification of the method factor, including whether its loadings were constrained to equality and whether the model converged without inadmissible estimates. The relevant Supplementary Tables S1–S3 should also be made fully accessible to reviewers. I do not consider it necessary to provide every line of raw software output, but the manuscript and supplement must contain enough information for readers to understand and evaluate the model specification.

The authors should additionally explain why the Harman test results changed between the original and revised manuscripts. The original manuscript reported ten factors with eigenvalues greater than one and a first-factor variance of 23.769%, whereas the revised manuscript reports nine factors and 25.214%. Because the sample remained the same, the change suggests that the set of included items, scoring, or data-processing procedure may have changed. This should be clarified so that the common-method assessment can be interpreted consistently.

Major Comment 3: This is a new concern arising from the revised analysis. The authors added sport item and sport level as covariates in response to concerns about sport-specific confounding, and the revised Methods section indicates that these variables were included together with gender, age, family structure, and residence. The response letter refers to the revised covariate specification around Line 303 and Table 4.

Table 4, however, appears to report only one coefficient for each of the variables family structure, residence, sport item, and sport level. The model degrees of freedom also appear consistent with each multicategory variable being entered as a single numeric predictor. If this interpretation is correct, the analysis imposes a linear and equally spaced ordering on categories that are not naturally continuous. For example, combat sports, ball sports, track and field, aquatic sports, and other sports do not constitute a meaningful numerical continuum. The same concern applies to family-structure and residence categories.

The authors should clarify exactly how these variables were coded in PROCESS. If appropriate indicator coding was already used, the reference categories and category-specific coefficients should be reported clearly in the table or Supplementary Materials. If integer codes were entered directly as single predictors, the models should be rerun using appropriate (k-1) dummy variables for nominal categories. Sport level may be treated as ordinal only if the authors can justify the assumed linear trend; otherwise, it should also be represented through indicator variables. Any required reanalysis should be reflected consistently in the mediation effects, interaction terms, confidence intervals, effect sizes, figures, and conclusions. The VIF and tolerance diagnostics should also be updated after the final coding specification is established.

Major Comment 4: The revised Discussion provides a much more balanced interpretation of the ATHB × CR interaction. In Lines 535–552, the authors explicitly acknowledge that cognitive reappraisal strengthened the positive association between athlete burnout and academic burnout and therefore should not be treated as a uniformly protective resource. The discussion of possible strategy exhaustion, reduced effectiveness under severe burnout, and overlap with repeated negative cognitive processing is thoughtful, provided that these explanations remain clearly identified as tentative.

This balanced interpretation is not yet fully reflected in the Abstract and Conclusion. The Abstract continues to state that CR “strengthens” the buffering effect of PSS and uses the result to support interventions combining social support enhancement with emotion-regulation training. It also states that PSS “predicted” AB despite the cross-sectional design. The Conclusion similarly tends to characterize higher CR as amplifying the protective role of PSS, without giving equal emphasis to the fact that higher CR also strengthened the positive ATHB–AB association.

The Abstract and Conclusion should explicitly state that the moderating role of CR was path-specific. Higher CR strengthened the negative PSS–AB association, but it also strengthened the positive ATHB–AB association. This dual pattern is theoretically important and should not be reduced to a general buffering interpretation. The authors should also continue revising residual causal or directional expressions such as “predicted,” “protective factor,” “reduces,” “prevents,” and “mechanism,” where these terms imply temporal or causal evidence. The serial mediation findings should be described as theory-consistent indirect associations rather than demonstrated processes of resource transformation. The practical recommendations may remain, but they should be framed as implications requiring longitudinal or experimental testing, rather than as interventions shown by the present study to be effective.

Minor Comments

Minor Comment 1: The authors now clearly state that the two studies used the same cross-sectional survey and shared several variables, and they distinguish the earlier burnout-spillover model from the present resource-oriented model. The revisions around Lines 171–182 and 417–426 represent a substantial improvement and largely address my previous concern.

One further clarification would strengthen transparency. The authors should indicate whether the present moderated serial mediation model was specified before or after the analysis reported in the earlier publication, and whether any part of the current analysis should be regarded as exploratory. This does not require the authors to demonstrate formal preregistration or reconstruct a detailed historical timeline; a clear and candid statement would be sufficient.

Minor Comment 2: The coefficient reported in the Abstract, β=−0.123, does not represent the total association between perceived social support and academic burnout. Rather, it represents the direct association after accounting for psychological resilience, athlete burnout, and the covariates. Table 3 reports the total effect as −0.325. The Abstract should therefore identify β=−0.123 explicitly as the direct effect or direct association, so that readers do not misinterpret it as the overall association between PSS and AB.

The manuscript should also distinguish more carefully between the interaction effect supporting H7 and the moderated indirect effects. H7 concerns whether cognitive reappraisal moderates the ATHB→AB path, and the most direct evidence for this hypothesis is the significant ATHB×CR interaction. By contrast, the conditional indirect effects of PSS on AB through ATHB at low and high levels of CR, together with the corresponding index of moderated mediation, address whether the entire PSS→ATHB→AB indirect pathway varies as a function of CR. These statistics are related, but they are not interchangeable forms of evidence for the same hypothesis.

Accordingly, the authors should report the path interaction, the conditional indirect effects, and the index of moderated mediation as conceptually distinct results. The moderated mediation index of −0.011 should be described as evidence that the PSS→ATHB→AB indirect association varies across levels of CR, rather than simply as additional support for H7. By contrast, the serial moderated mediation index of −0.017 corresponds more directly to H8, which concerns the conditional indirect association through the full PSS→PR→ATHB→AB pathway.

Minor Comment 3: Although the overall language quality of the revised manuscript has improved, several clearly identifiable grammatical and formatting problems remain. For example, the phrase “CR significantly moderate” in Lines 391–393 contains an incorrect verb form, and the sentence “PSS significantly and negatively associated with AB” in Lines 621–622 is missing the auxiliary verb “was.” Lines 327–330, as well as several tables and figure notes, also contain inconsistent statistical formatting, including singular/plural errors in “p value,” spacing errors such as “p <0 .01,” mixed use of English and full-width Chinese punctuation, and duplicated periods.

The reference list also requires further checking. The same article by Shang and Yang appears to be listed repeatedly as References 34, 64, and 67. In addition, Lines 560–562 cite Reference 70 to support the statement that cognitive reappraisal did not significantly moderate the association between stress and mental health. However, the cited article concerns the mediating roles of interpersonal emotion regulation and social self-efficacy in the association between autistic traits and depression, and therefore does not directly support the claim made in the manuscript.

The authors should undertake a systematic English-language and reference audit after completing the methodological and statistical revisions. Particular attention should be paid to grammar, statistical symbols and spacing, figure-note formatting, duplicate references, and whether each cited source directly supports the corresponding statement in the text.

**********

what does this mean?). If published, this will include your full peer review and any attached files.

If you choose “no”, your identity will remain anonymous but your review may still be made public.

**Do you want your identity to be public for this peer review?**  For information about this choice, including consent withdrawal, please see our Privacy Policy

Reviewer #1: No

Reviewer #2: No

**********

[NOTE: If reviewer comments were submitted as an attachment file, they will be attached to this email and accessible via the submission site. Please log into your account, locate the manuscript record, and check for the action link "View Attachments". If this link does not appear, there are no attachment files.]

To ensure your figures meet our technical requirements, please review our figure guidelines: https://journals.plos.org/plosone/s/figures

You may also use PLOS’s free figure tool, NAAS, to help you prepare publication quality figures: https://journals.plos.org/plosone/s/figures#loc-tools-for-figure-preparation.

NAAS will assess whether your figures meet our technical requirements by comparing each figure against our figure specifications.

# Author comment: Author response to Decision Letter 2

**DOI:** 10.1371/journal.pone.0361222.r004

19 Aug 2026

Dear Editor,

Thank you for reviewing our manuscript. At the same time, give us the opportunity to make modifications. This revision has provided point-to-point responses to the specific questions raised by the reviewers. Thank you very much for your work. If you feel that there are any further modifications needed, please feel free to point them out.

Dear reviewer,

Thank you for carefully reviewing our manuscript and providing many helpful suggestions. We have made corresponding modifications to the manuscript based on your suggestions. We believe that with your help, the quality of our manuscript has been greatly improved. We hope that this modification can meet your requirements. If there are any shortcomings, please feel free to raise them again.

Once again, I would like to express my sincere gratitude for your patience and meticulousness.

Major Comments

Major Comment 1: The authors have responded constructively to my previous concerns about the transition from the measurement label “sport mental toughness” to the analytical label “psychological resilience.” In the revised Methods section, around Lines 275–282, the authors now state explicitly that the SMTQ total score was used to operationalize a sport-specific resilience-related psychological resource rather than general psychological resilience. The Limitations section also acknowledges that the SMTQ was not originally designed as a dedicated measure of psychological resilience. These additions improve the transparency of the manuscript.

Nevertheless, the underlying construct-validity issue remains only partially resolved. Sport mental toughness and psychological resilience occupy related conceptual territory, but they are not fully interchangeable. The SMTQ assesses confidence, constancy, and control under pressure, whereas resilience more specifically concerns adaptation, recovery, and sustained functioning following adversity. The newly added CFA, AVE, and HTMT analyses can demonstrate that the SMTQ-based factor is empirically distinguishable from PSS, CR, ATHB, and AB, but they cannot establish that the SMTQ is a direct measure of psychological resilience. Discriminant validity from other variables is not equivalent to content or convergent validity as resilience. The manuscript should therefore avoid presenting the mediator unqualifiedly as psychological resilience. The authors may either relabel the construct as “sport mental toughness” or consistently describe it as an “SMTQ-based resilience-related psychological resource in the sport context,” but the terminology and interpretation must be aligned throughout the Introduction, hypotheses, figures, tables, Results, Discussion, and Conclusion. Claims that the study directly demonstrates a psychological-resilience mechanism should be narrowed accordingly.

A related scoring issue also requires clarification. The original manuscript stated that SMTQ Items 2, 4, 7, 8, 9, and 10 were reverse-coded, whereas the revised description appears to omit Item 10. The original scoring description is located in the SMTQ subsection, Lines 258–268. The authors should verify the scoring key actually used to compute the analytic variable and state clearly which description is correct. If the dataset was scored correctly and the inconsistency is only textual, an explicit confirmation and correction would be sufficient. If the scoring computation itself differed, the descriptive statistics, reliability analysis, CFA results, correlations, and PROCESS models involving this mediator would need to be recalculated.

Response: Thank you for this important and constructive comment. We agree that sport mental toughness and psychological resilience are conceptually related but distinct constructs and that the CFA, AVE, and HTMT analyses cannot establish the SMTQ as a direct measure of psychological resilience. We also appreciate the reviewer’s identification of the related inconsistency in the reported SMTQ reverse-scoring key. We address the construct-validity and scoring issues separately below.

A. Construct clarification and terminology alignment

Following the reviewer’s recommendation, we have relabeled the mediator from psychological resilience (PR) to mental toughness (MT) throughout the manuscript and revised its conceptualization, measurement description, reporting, and interpretation accordingly.

(1) Because the SMTQ was originally developed to assess mental toughness rather than psychological resilience, the revised Introduction now conceptualizes MT as a psychological advantage manifested in the sport context, enabling athletes to maintain determination, focus, confidence, and control when coping with the demands of competition, training, and the athletic lifestyle. We also reconstructed the theoretical rationale using MT-related evidence concerning PSS, AB, ATHB, and school-adjustment outcomes. Accordingly, all hypotheses previously involving PR have been revised to refer to MT. These include the individual mediation pathway through MT, the sequential mediation pathway through MT and ATHB, the moderation of the MT–AB association by CR, and the moderated indirect pathway involving MT and ATHB. The corresponding hypotheses, figures, and model descriptions have been revised consistently, and no hypothesis now refers to a psychological-resilience mechanism.

Revisions: Introduction (Lines 109–128, Lines 143-144, Lines 193-199), Figure 1.

(2) Revisions to the Methods. The Methods section now states that the SMTQ was used to assess college athletes’ MT in coping with competitive stress and performance-related challenges and identifies confidence, constancy, and control as its three dimensions. The previous paragraph justifying the SMTQ as a psychological resilience-related measure has been removed.

Revisions: Methods, Section 2.1.5 (Lines 290-299).

(3) Revisions to the Results, figures, and tables. The terms “psychological resilience” and “PR” have been replaced with “mental toughness” and “MT” in the Results, figures, and tables. The CFA, AVE, and HTMT findings are now interpreted only as evidence that MT is empirically distinguishable from PSS, CR, ATHB, and AB, rather than as evidence that the SMTQ has content or convergent validity as a measure of psychological resilience.

Revisions: Results, Sections 3.2-3.4; Figures 2-3; Tables 2-4; and Supplementary Tables S1-S5.

Revisions to the Discussion, Conclusion, and Limitations. In Sections 4.2 and 4.4, the mediation and sequential mediation findings are now interpreted as indirect associations involving MT, rather than as evidence of a psychological-resilience mechanism. Where research on resilience is retained in Section 4.2, it is used only as complementary support for a broader resource-based perspective, with resilience and MT explicitly treated as distinct constructs. In Section 4.6, because the mediator is no longer conceptualized as psychological resilience, we removed the previous limitation concerning the use of the SMTQ instead of a dedicated psychological-resilience measure. The Conclusion has also been revised by replacing PR with MT and aligning the summary and implications with the revised construct interpretation.

Revisions: Discussion, Sections 4.2 and 4.4 (Lines 528–535), and 4.6; Conclusion (Lines 669-681).

These revisions align the mediator’s terminology, conceptual definition, measurement, empirical reporting, and theoretical interpretation throughout the manuscript. We appreciate the reviewer’s guidance, which substantially improved the construct clarity and interpretive precision of the study.

B.Clarification of the SMTQ scoring key

The omission of Item 10 from the revised description reflects the correction of a textual error in the original manuscript rather than a change in the scoring procedure. The original SMTQ contains 14 items, whereas the culturally adapted Chinese version administered in the present study contains 12 items. Because original Items 8 and 11 are not retained in the 12-item Chinese version, the subsequent items were renumbered. Specifically, the negatively worded concentration item, which is Item 10 in the original version, is Item 9 in the 12-item Chinese version. Therefore, the item numbering and reverse-scoring key of the original 14-item version cannot be directly applied to the 12-item version.

In the original manuscript, we mistakenly reported the reverse-scored item numbers of the 14-item version. In the 12-item Chinese version actually administered in this study, Items 2, 4, 7, 8, and 9 are negatively worded and were reverse-coded. Thus, the removal of Item 10 from the list of reverse-scored items in the revised manuscript reflects a correction of the original textual description. We apologize for not explaining the reason for this change in our previous response.

We rechecked the scoring procedure and confirmed that the dataset was scored according to the correct scoring key for the 12-item Chinese version. Therefore, this inconsistency was confined to the description in the original manuscript and did not affect the descriptive statistics, reliability estimates, CFA results, correlations, or PROCESS analyses.

Major Comment 2: The authors have undertaken the additional psychometric analyses requested in the previous review. The revised manuscript reports that the hypothesized five-factor model achieved acceptable fit and outperformed alternative models, and that AVE and HTMT results supported discriminant validity. The authors also added an unmeasured latent method construct model to supplement Harman’s single-factor test. These additions are directionally appropriate and represent a substantive response to my earlier comments.

However, the manuscript does not explain how the observed indicators in the CFA were constructed. The five measures contain a large number of individual items, yet the reported five-factor model has only 125 degrees of freedom. This suggests that the authors may have used subscale scores, item parcels, or another reduced-indicator approach, but the relevant procedure is not described. The authors should state clearly whether the CFA was conducted using individual items, scale dimensions, or parcels. If parcels were used, the parcel-construction procedure and its rationale should be reported, particularly because both academic burnout and athlete burnout are multidimensional constructs.

The reported baseline CFA and ULMC models also have exactly the same chi-square value, χ2=1127.001, despite differing by one degree of freedom. This is not necessarily impossible, but it is sufficiently unusual to require verification. The authors should confirm that the values were not duplicated inadvertently and briefly report the specification of the method factor, including whether its loadings were constrained to equality and whether the model converged without inadmissible estimates. The relevant Supplementary Tables S1–S3 should also be made fully accessible to reviewers. I do not consider it necessary to provide every line of raw software output, but the manuscript and supplement must contain enough information for readers to understand and evaluate the model specification.

The authors should additionally explain why the Harman test results changed between the original and revised manuscripts. The original manuscript reported ten factors with eigenvalues greater than one and a first-factor variance of 23.769%, whereas the revised manuscript reports nine factors and 25.214%. Because the sample remained the same, the change suggests that the set of included items, scoring, or data-processing procedure may have changed. This should be clarified so that the common-method assessment can be interpreted consistently.

Response: Thank you for identifying the lack of information concerning the construction of the CFA indicators and the specification of the ULMC model. We have now clarified both issues in the revised manuscript and Supplementary Materials.

First, the five-factor CFA was conducted using 18 observed indicators: three a priori dimension-level sum scores from each of the MSPSS, SMTQ, ABQ, and LBUS, together with the six individual cognitive-reappraisal items. Each dimension score was calculated according to the established structure and scoring rules of the corresponding validated subscale. Thus, these indicators were theory-based dimension-level composites rather than ad hoc or data-driven item parcels. This approach preserved the established multidimensional structures of the measures, particularly academic burnout and athlete burnout, while maintaining a parsimonious measurement model. The 18-indicator specification is also consistent with the reported 125 degrees of freedom. This information has been added to the Statistical Analysis section (Lines 303-309).

Second, we rechecked the originally reported ULMC analysis and confirmed that the identical chi-square value was not caused by inadvertent duplication. In the original ULMC model, all unstandardized method-factor loadings were constrained to equality, the variance of the method factor was fixed to 1 for identification, and its covariances with the five substantive factors were constrained to zero. Because the equality constraint introduced only one additional freely estimated loading parameter, the model had one fewer degree of freedom than the baseline CFA model. Although the software reported convergence, the common method loading was estimated as 0.000 with an extremely large standard error. The method factor therefore collapsed to a poorly determined zero-effect solution and produced no reduction in chi-square, explaining why the model yielded the same chi-square value as the baseline CFA. Accordingly, the equality-constrained model could not be regarded as a stable and substantively informative ULMC solution.

We therefore replaced the equality-constrained model with a less restrictive ULMC model in which the method-factor loadings were freely estimated on all 18 indicators. The method-factor variance was fixed to 1, and its covariances with the five substantive factors were constrained to zero. The revised model converged without negative variance estimates or standardized loadings exceeding 1.00 and showed improved fit, χ²(107) = 701.894, CFI = 0.971, TLI = 0.959, RMSEA = 0.054, and SRMR = 0.036. Inspection of the parameter estimates showed comparatively high method-factor loadings on the cognitive-reappraisal indicators, accompanied by markedly attenuated loadings on the substantive CR factor. This pattern indicated limited empirical separation between the variance attributed to the method factor and the substantive CR factor. We therefore did not attribute the improvement in model fit uniquely to common method variance. Instead, the revised manuscript now acknowledges nontrivial method-related shared covariance, particularly among the CR indicators, and notes that findings involving CR, especially the moderation effects, should be interpreted cautiously and replicated using temporally separated or multi-method designs. These revisions have been incorporated into Section 3.1(Lines 342-357) and the Limitations section (Lines 646-654 and Lines 655-661).

Supplementary Table S1 now reports the fit indices for the hypothesized five-factor CFA model, four alternative measurement models, and the freely loading ULMC model. Supplementary Table S2 presents composite reliability, AVE, construct correlations, and the square roots of AVE, whereas Supplementary Table S3 reports the HTMT ratios among the five constructs. We also added a concise summary of these results to Section 3.2 of the revised manuscript. Composite reliability values ranged from 0.770 to 0.916, AVE values ranged from 0.533 to 0.784, and HTMT ratios ranged from 0.379 to 0.727. For each construct, the square root of AVE exceeded all corresponding interconstruct correlations. Together, the composite reliability and AVE results supported convergent validity, whereas the Fornell–Larcker criterion and HTMT results supported discriminant validity. The complete results are presented in the revised Supplementary Materials.

Revisions: Results, Section 3.2 (Lines 369–373); Supplementary Tables S1–S3.

Third, the difference between the two Harman test results arose from the set of ERQ items included. In the original analysis, the expressive-suppression items, which were not part of the hypothesized model, were inadvertently included together with the cognitive-reappraisal items. The revised analysis included only the cognitive-reappraisal items, consistent with the construct examined in the present model. Apart from this adjustment to the included items, the sample, scoring procedures, data-processing criteria, and factor-extraction method remained unchanged. The revised analysis yielded nine factors with eigenvalues greater than 1, with the first factor accounting for 25.214% of the total variance. This result has been retained in the revised manuscript. We apologize for not explaining this change in our previous response.

Major Comment 3: This is a new concern arising from the revised analysis. The authors added sport item and sport level as covariates in response to concerns about sport-specific confounding, and the revised Methods section indicates that these variables were included together with gender, age, family structure, and residence. The response letter refers to the revised covariate specification around Line 303 and Table 4.

Table 4, however, appears to report only one coefficient for each of the variables family structure, residence, sport item, and sport level. The model degrees of freedom also appear consistent with each multicategory variable being entered as a single numeric predictor. If this interpretation is correct, the analysis imposes a linear and equally spaced ordering on categories that are not naturally continuous. For example, combat sports, ball sports, track and field, aquatic sports, and other sports do not constitute a meaningful numerical continuum. The same concern applies to family-structure and residence categories.

The authors should clarify exactly how these variables were coded in PROCESS. If appropriate indicator coding was already used, the reference categories and category-specific coefficients should be reported clearly in the table or Supplementary Materials. If integer codes were entered directly as single predictors, the models should be rerun using appropriate (k-1) dummy variables for nominal categories. Sport level may be treated as ordinal only if the authors can justify the assumed linear trend; otherwise, it should also be represented through indicator variables. Any required reanalysis should be reflected consistently in the mediation effects, interaction terms, confidence intervals, effect sizes, figures, and conclusions. The VIF and tolerance diagnostics should also be updated after the final coding specification is established.

Response: Thank you for raising this important methodological concern. We agree that treating multicategorical variables as single numeric predictors would impose inappropriate assumptions of linearity and equal intervals among categories.

Upon re-examination, we confirmed that family structure, residence, sport item, and sport level had previously been entered as single predictors using their original integer codes. We therefore recoded these variables using k-1 indicator variables and reran all relevant analyses. Gender was represented by one binary indicator, with male as the reference category. Family structure was represented by three indicators, with intact family as the reference category; residence by three indicators, with urban residence as the reference category; sport item by four indicators, with combat sports as the reference category; and sport level by three indicators, with National Level-2 Athlete as the reference category. Age remained a continuous covariate. Thus, no assumptions of linear ordering or equal spacing were imposed on the categorical variables.

Using the revised coding scheme, we reran the serial mediation model and the moderated serial mediation model, including all direct and indirect effects, moderated indirect effects, interaction terms, bootstrap confidence intervals, simple-slope analyses, and effect-size estimates. The revised model degrees of freedom are consistent with the final indicator coding. The overall pattern and statistical significance of the hypothesized associations remained unchanged, and the substantive conclusions were not altered. The corresponding tables, figures, and conclusions were checked and updated where necessary.

Multicollinearity diagnostics were also recalculated using the final coding specification. VIF values ranged from 1.016 to 1.686, and tolerance values ranged from 0.593 to 0.984, indicating no evidence of problematic multicollinearity.

The revisions have been incorporated throughout the manuscript. Specifically, the Statistical Analysis section (Section 2.2, Lines 318-321) was revised to clarify the coding procedures and reference categories. The serial mediation and moderated serial mediation analyses, corresponding tables, and supplementary materials (Tables S4 and S5) were updated based on the revised models.

Major Comment 4: The revised Discussion provides a much more balanced interpretation of the ATHB × CR interaction. In Lines 535–552, the authors explicitly acknowledge that cognitive reappraisal strengthened the positive association between athlete burnout and academic burnout and therefore should not be treated as a uniformly protective resource. The discussion of possible strategy exhaustion, reduced effectiveness under severe burnout, and overlap with repeated negative cognitive processing is thoughtful, provided that these explanations remain clearly identified as tentative.

This balanced interpretation is not yet fully reflected in the Abstract and Conclusion. The Abstract continues to state that CR “strengthens” the buffering effect of PSS and uses the result to support interventions combining social support enhancement with emotion-regulation training. It also states that PSS “predicted” AB despite the cross-sectional design. The Conclusion similarly tends to characterize higher CR as amplifying the protective role of PSS, without giving equal emphasis to the fact that higher CR also strengthened the positive ATHB–AB association.

The Abstract and Conclusion should explicitly state that the moderating role of CR was path-specific. Higher CR strengthened the negative PSS–AB association, but it also strengthened the positive ATHB–AB association. This dual pattern is theoretically important and should not be reduced to a general buffering interpretation. The authors should also continue revising residual causal or directional expressions such as “predicted,” “protective factor,” “reduces,” “prevents,” and “mechanism,” where these terms imply temporal or causal evidence. The serial mediation findings should be described as theory-consistent indirect associations rather than demonstrated processes of resource transformation. The practical recommendations may remain, but they should be framed as implications requiring longitudinal or experimental testing, rather than as interventions shown by the present study to be effective.

Response: Thank you for this important comment. We agree that the path-specific role of cognitive reappraisal (CR), the cross-sectional nature of the data, and the tentative status of the proposed explanations and practical implications needed to be represented more consistently throughout the manuscript. Accordingly, we made the following revisions:

First, the Abstract and Conclusion now give equal emphasis to the two significant moderation effects. Specifically, they state that, at higher levels of CR, the negative association between perceived social support (PSS) and academic burnout (AB) was stronger, but the positive association between athlete burnout (ATHB) and AB was also stronger, whereas the association between MT and AB did not differ significantly across CR levels. We therefore explicitly characterize the moderating role of CR as path-specific rather than uniformly buffering. These revisions have been incorporated into Abstract (Lines 18-21) and the Conclusion (Lines 673-677).

Second, we systematically reviewed causal and directional expressions throughout the manuscript and revised those that could imply temporal or causal evidence from the present cross-sectional findings. PSS is now described as being associated with, rather than predicting, AB. The mediation findings are described as direct and indirect associations, and the PSS → MT → ATHB → AB sequence is explicitly presented as a theory-driven statistical model rather than evidence of a causal chain or demonstrated resource-transformation process.

Third, the proposed explanations for the ATHB × CR interaction, including reduced reappraisal effectiveness under elevated burnout, strategy exhaustion, and overlap with rumination or repetitive negative processing, are now explicitly identified as speculative interpretations (see Lines 593-598).

Finally, the practical implications have been revised to avoid implying that the present study established intervention effectiveness. The manuscript now states that PSS, MT, ATHB, and CR-related training may inform future multicomponent support approaches, but that their effectiveness and boundary conditions require longitudinal or experimental evaluation. These revisions have been made in the Abstract (Lines 21–25) and the Conclusion (Lines 677–681).

Minor Comments

Minor Comment 1: The authors now clearly state that the two studies used the same cross-sectional survey and shared several variables, and they distinguish the earlier burnout-spillover model from the present resource-oriented model. The revisions around Lines 171–182 and 417–426 represent a substantial improvement and largely address my previous concern.

One further clarification would strengthen transparency. The authors should indicate whether the present moderated serial mediation model was specified before or after the analysis reported in the earlier publication, and whether any part of the current analysis should be regarded as exploratory. This does not require the authors to demonstrate formal preregistration or reconstruct a detailed historical timeline; a clear and candid statement would be sufficient.

Response: Thank you for this helpful suggestion. The moderated serial mediation model was developed after completion of the analyses reported in the earlier publication but before the analyses reported in the present study were conducted. The analyses were not formally preregistered. However, the focal model and Hypotheses H1–H8 were specified before the PROCESS analyses, and no component of the reported moderated serial mediation analysis was conducted as a post hoc exploratory analysis. This clarification has been added to Section 2.2 (Statistical Analysis) of the revised manuscript (Lines 325–327).

Minor Comment 2: The coefficient reported in the Abstract, β=−0.123, does not represent the total association between perceived social support and academic burnout. Rather, it represents the direct association after accounting for psychological resilience, athlete burnout, and the covariates. Table 3 reports the total effect as −0.325. The Abstract should therefore identify β=−0.123 explicitly as the direct effect or direct association, so that readers do not misinterpret it as the overall association between PSS and AB.

The manuscript should also distinguish more carefully between the interaction effect supporting H7 and the moderated indirect effects. H7 concerns whether cognitive reappraisal moderates the ATHB→AB path, and the most direct evidence for this hypothesis is the significant ATHB×CR interaction. By contrast, the conditional indirect effects of PSS on AB through ATHB at low and high levels of CR, together with the corresponding index of moderated mediation, address whether the entire PSS→ATHB→AB indirect pathway varies as a function of CR. These statistics are related, but they are not interchangeable forms of evidence for the same hypothesis.

Accordingly, the authors should report the path interaction, the conditional indirect effects, and the index of moderated mediation as conceptually distinct results. The moderated mediation index of −0.011 should be described as evidence that the PSS→ATHB→AB indirect association varies across levels of CR, rather than simply as additional support for H7. By contrast, the serial moderated mediation index of −0.017 corresponds more directly to H8, which concerns the conditional indirect association through the full PSS→PR→ATHB→AB pathway.

Response: Thank you for this important clarification. We agree with the reviewer and have revised both the Abstract and the Results section accordingly.

First, we revised the Abstract to explicitly identify the reported coefficient as the direct association between PSS and AB: “PSS was directly and negatively associated with AB (β = −0.122, p < 0.001).” Following the reanalysis using the revised dummy-coded covariate specification, the coefficient changed slightly from −0.123 to −0.122. The total association (β = −0.322) remains reported separately in Table 3.

Second, Section 3.4 has been revised to distinguish the evidence supporting H7 from the moderated indirect effects. The significant ATHB × CR interaction and the corresponding simple slope results are now presented as the direct evidence supporting H7. The conditional indirect effects of PSS on AB through ATHB and the index of moderated mediation (−0.011, 95% CI [−0.020, −0.004]) are reported separately as evidence that the PSS → ATHB → AB indirect pathway varied as a function of CR, rather than as additional support for H7. In contrast, the index of moderated mediation for the serial indirect effect (−0.017, 95% CI [−0.027, −0.007]) is explicitly linked to H8 because it indicates that the full PSS → MT → ATHB → AB serial indirect association varied as a function of CR. These revisions are presented in Lines 410–419.

Minor Comment 3: Although the overall language quality of the revised manuscript has improved, several clearly identifiable grammatical and formatting problems remain. For example, the phrase “CR significantly moderate” in Lines 391–393 contains an incorrect verb form, and the sentence “PSS significantly and negatively associated with AB” in Lines 621–622 is missing the auxiliary verb “was.” Lines 327–330, as well as several tables and figure notes, also contain inconsistent statistical formatting, including singular/plural errors in “p value,” spacing errors such as “p <0 .01,” mixed use of English and full-width Chinese punctuation, and duplicated periods.

The reference list also requires further checking. The same article by Shang and Yang appears to be listed repeatedly as References 34, 64, and 67. In addition, Lines 560–562 cite Reference 70 to support the statement that cognitive reappraisal did not significantly moderate the association between stress and mental health. However, the cited article concerns the mediating roles of interpersonal emotion regulation and social self-efficacy in the association between autistic traits and depression, and therefore does not directly support the claim made in the manuscript.

The authors should undertake a systematic English-language and reference audit after completing the methodological and statistical revisions. Particular attention should be paid to grammar, statistical symbols and spacing, figure-note formatting, duplicate references, and whether each cited source directly supports the corresponding statement in the text.

Response: Thank you for this careful review. We have conducted a systematic language, formatting, and reference audit throughout the manuscript. Specifically, we corrected the identified grammatical errors, including “CR significantly moderate” to “CR significantly moderated” and “PSS significantly and negatively associated with AB” to “PSS was significantly and negatively associated with AB.” We also standardized statistical reporting across the text, tables, and figure notes, including the singular/plural use of p value(s), spacing (e.g., p < 0.01), punctuation, and duplicated periods.

Third, the duplicate entries for Shang and Yang (2021), previously listed as References 34, 64, and 67, were consolidated into a single reference, and all subsequent reference numbers and corresponding in-text citations were updated. The relevant discussion was also revised to clarify that this study supports the adjacent association from social support through MT to ATHB, rather than the complete serial indirect pathway examined in the present study. Please see Lines 528–535.

We agree that the former Reference 70 did not support the original statement regarding the nonsignificant moderating role of cognitive reappraisal. The inappropriate citation and unsupported statement were therefore removed, and the discussion was revised using a more relevant, but explicitly described as partially analogous, study. Please see Lines 608- 611.

We also conducted an additional cross-check of the reference list to ensure consistency between the cited claims and their supporting sources.

Attachment Submitted filename: response_letter_for_review_comments_auresp_2.docx

# Peer review: Decision Letter 2

**DOI:** 10.1371/journal.pone.0361222.r005

**Authors:** Rogis Baker

11 Sep 2026

Dear Dr.  Xu,

Thank you for submitting your manuscript to PLOS One. After careful consideration, we feel that it has merit but does not fully meet PLOS One’s publication criteria as it currently stands. Therefore, we invite you to submit a revised version of the manuscript that addresses the points raised during the review process.

Please submit your revised manuscript by Oct 26 2026 11:59PM. If you will need more time than this to complete your revisions, please reply to this message or contact the journal office at plosone@plos.org. When you're ready to submit your revision, log on to https://www.editorialmanager.com/pone/ and select the 'Submissions Needing Revision' folder to locate your manuscript file.

- A letter that responds to each point raised by the academic editor and reviewer(s). You should upload this letter as a separate file labeled 'Response to Reviewers'.
- A marked-up copy of your manuscript that highlights changes made to the original version. You should upload this as a separate file labeled 'Revised Manuscript with Track Changes'.
- An unmarked version of your revised paper without tracked changes. You should upload this as a separate file labeled 'Manuscript'.

If you would like to make changes to your financial disclosure, please include your updated statement in your cover letter. Guidelines for resubmitting your figure files are available below the reviewer comments at the end of this letter.

If applicable, we recommend that you deposit your laboratory protocols in protocols.io to enhance the reproducibility of your results. Protocols.io assigns your protocol its own identifier (DOI) so that it can be cited independently in the future. For instructions see: https://journals.plos.org/plosone/s/submission-guidelines#loc-laboratory-protocols. Additionally, PLOS One offers an option for publishing peer-reviewed Lab Protocol articles, which describe protocols hosted on protocols.io. Read more information on sharing protocols at https://plos.org/protocols?utm_medium=editorial-email&utm_source=authorletters&utm_campaign=protocols.

As the corresponding author, your ORCID iD is verified in the submission system and will appear in the published article. PLOS supports the use of ORCID, and we encourage all coauthors to register for an ORCID iD and use it as well. Please encourage your coauthors to verify their ORCID iD within the submission system before final acceptance, as unverified ORCID iDs will not appear in the published article. *Only*  the individual author can complete the verification step; PLOS staff *cannot*  verify ORCID iDs on behalf of authors.

We look forward to receiving your revised manuscript.

Kind regards,

Rogis Baker, Ph.D

Academic Editor

PLOS One

Journal Requirements:

1. If the reviewer comments include a recommendation to cite specific previously published works, please review and evaluate these publications to determine whether they are relevant and should be cited. There is no requirement to cite these works unless the editor has indicated otherwise.

2. Please review your reference list to ensure that it is complete and correct. If you have cited papers that have been retracted, please include the rationale for doing so in the manuscript text, or remove these references and replace them with relevant current references. Any changes to the reference list should be mentioned in the rebuttal letter that accompanies your revised manuscript. If you need to cite a retracted article, indicate the article’s retracted status in the References list and also include a citation and full reference for the retraction notice.

[Note: HTML markup is below. Please do not edit.]

Reviewer's Responses to Questions

**Comments to the Author**

Reviewer #2: (No Response)

**********

2. Is the manuscript technically sound, and do the data support the conclusions?

Reviewer #2: Yes

**********

3. Has the statistical analysis been performed appropriately and rigorously?

Reviewer #2: Yes

**********

4. Have the authors made all data underlying the findings in their manuscript fully available?

The PLOS Data policy

Reviewer #2: Yes

**********

5. Is the manuscript presented in an intelligible fashion and written in standard English?

Reviewer #2: Yes

**********

Reviewer #2: Overall Assessment

The authors have satisfactorily addressed the principal methodological and interpretive concerns raised in the previous round. In particular, the construct terminology has been corrected, the multicategorical covariates have been recoded appropriately, and the mediation and moderated mediation results are now presented and interpreted more clearly. No major concerns remain. The following minor issues should be addressed before publication.

Minor Concerns

Q1:Lines 218–220 state that 2,115 questionnaires were distributed and 1,967 were returned, corresponding to a response rate of 93.00%. Lines 220–222 further report that 49 questionnaires were excluded and that 1,918 respondents were retained, but describe 97.51% as the “effective response rate.” The latter percentage represents the proportion of usable questionnaires among the questionnaires returned (1,918/1,967), whereas the proportion of usable questionnaires among all questionnaires distributed is 90.69% (1,918/2,115). Please revise the terminology at Lines 218–222 to distinguish clearly between the questionnaire return rate, the proportion of usable questionnaires among returned questionnaires, and the overall usable response rate.

Lines 220–221 also refer to “repetitive patterns” and “logical errors” as exclusion criteria. Please briefly clarify how these criteria were defined and applied during data screening.

Q2:At Line 190, H1 is formulated as the hypothesis that PSS is negatively correlated with AB. The zero-order correlation reported in Table 2 at Line 356 ((r=-0.327), (p<0.01)) provides the direct evidence relevant to this hypothesis. However, Lines 371–372 state that the adjusted direct association between PSS and AB (effect = −0.122, 95% CI [−0.170, −0.073]) supports H1.

These two statistics represent different quantities. Please revise Lines 371–372 so that the correlation reported in Table 2 is used as the primary evidence for H1. The coefficient reported in Table 3 should be identified separately as the direct association between PSS and AB after accounting for the proposed mediators and covariates.

3. Lines 257–263 explain that the ERQ comprises the cognitive reappraisal and expressive suppression dimensions, while only the CR subscale was included in the model. Lines 266–267 subsequently report Cronbach’s (Cronbach’s α=0.828) for the ERQ and (Cronbach’s α=0.894) for CR. It is unclear which items were included in the calculation of the first coefficient and why the reliability of the complete ERQ is relevant when only CR was analyzed.

Please clarify at Lines 266–267 which items were used to calculate each reliability coefficient. Because only the CR subscale was included in the reported analyses, its reliability estimate should be emphasized. The reliability estimate for the complete ERQ should be removed if it is not relevant to the present analysis.

4. At Line 5, the institutional affiliation is not capitalized consistently. Please conduct a final systematic language and statistical-formatting check across the main text, tables, figure captions, and notes.

**********

what does this mean?). If published, this will include your full peer review and any attached files.

If you choose “no”, your identity will remain anonymous but your review may still be made public.

**Do you want your identity to be public for this peer review?**  For information about this choice, including consent withdrawal, please see our Privacy Policy

Reviewer #2: No

**********

[NOTE: If reviewer comments were submitted as an attachment file, they will be attached to this email and accessible via the submission site. Please log into your account, locate the manuscript record, and check for the action link "View Attachments". If this link does not appear, there are no attachment files.]

To ensure your figures meet our technical requirements, please review our figure guidelines: https://journals.plos.org/plosone/s/figures

You may also use PLOS’s free figure tool, NAAS, to help you prepare publication quality figures: https://journals.plos.org/plosone/s/figures#loc-tools-for-figure-preparation.

NAAS will assess whether your figures meet our technical requirements by comparing each figure against our figure specifications.

# Author comment: Author response to Decision Letter 3

**DOI:** 10.1371/journal.pone.0361222.r006

20 Sep 2026

Dear Editor,

Thank you very much for your careful consideration of our manuscript and for giving us the opportunity to revise it. We have carefully addressed all comments and suggestions raised by the reviewers and have provided detailed point-by-point responses. We sincerely appreciate the time and effort devoted by you and the reviewers to improving our manuscript. We hope that the revised manuscript is now suitable for publication. Should any further revisions be required, we would be pleased to address them.

Sincerely,

The Authors

Dear Reviewer,

Thank you very much for your careful review of our manuscript and for your valuable comments and suggestions. Your feedback has been highly helpful in improving the scientific rigor, clarity, and overall quality of the manuscript. We have carefully considered and addressed each of your comments and revised the manuscript accordingly. All changes have been highlighted in the revised manuscript for ease of reference. We sincerely appreciate the time and effort you devoted to evaluating our work. Should any further revisions or clarifications be required, we would be pleased to address them.

Sincerely,

The Authors

Minor Concerns

Q1:Lines 218–220 state that 2,115 questionnaires were distributed and 1,967 were returned, corresponding to a response rate of 93.00%. Lines 220–222 further report that 49 questionnaires were excluded and that 1,918 respondents were retained, but describe 97.51% as the “effective response rate.” The latter percentage represents the proportion of usable questionnaires among the questionnaires returned (1,918/1,967), whereas the proportion of usable questionnaires among all questionnaires distributed is 90.69% (1,918/2,115). Please revise the terminology at Lines 218–222 to distinguish clearly between the questionnaire return rate, the proportion of usable questionnaires among returned questionnaires, and the overall usable response rate.

Lines 220–221 also refer to “repetitive patterns” and “logical errors” as exclusion criteria. Please briefly clarify how these criteria were defined and applied during data screening.

Response: Thank you for this clarification. We have revised the participant-screening description to distinguish clearly among the questionnaire return rate, the proportion of usable questionnaires among returned questionnaires, and the overall usable response rate. Specifically, 1,967 of the 2,115 distributed questionnaires were returned (return rate: 93.00%), and 1,918 were retained for analysis, corresponding to 97.51% of the returned questionnaires and an overall usable response rate of 90.69%.

We also clarified the exclusion criteria used during manual screening. “Repetitive patterns” defined as invariant responding within all multi-item scales included in the questionnaire“Logical errors” has been revised to logical inconsistencies and was operationalized as contradictory responses regarding sport-specific training, such as reporting no weekly sport-specific training while also reporting at least one training day per week or at least 5 hours of weekly sport-specific training. These revisions have been incorporated into the Methods section. (Lines 218–226).

Q2:At Line 190, H1 is formulated as the hypothesis that PSS is negatively correlated with AB. The zero-order correlation reported in Table 2 at Line 356 ((r=-0.327), (p<0.01)) provides the direct evidence relevant to this hypothesis. However, Lines 371–372 state that the adjusted direct association between PSS and AB (effect = −0.122, 95% CI [−0.170, −0.073]) supports H1.

These two statistics represent different quantities. Please revise Lines 371–372 so that the correlation reported in Table 2 is used as the primary evidence for H1. The coefficient reported in Table 3 should be identified separately as the direct association between PSS and AB after accounting for the proposed mediators and covariates.

Response: Thank you for this important clarification. We agree that the zero-order Pearson correlation reported in Table 2 provides the evidence directly relevant to H1, whereas the coefficient reported in Table 3 represents the direct association between PSS and AB after accounting for the proposed mediators and covariates. Accordingly, we have revised Sections 3.2 and 3.3. H1 is now evaluated using the zero-order Pearson correlation between PSS and AB reported in Table 2 (r = −0.327, p < 0.01). The coefficient reported in Table 3 is now described separately as the significant negative direct association between PSS and AB after accounting for MT, ATHB, and the covariates (direct effect = −0.122, 95% CI [−0.170, −0.073]). The previous statement that this adjusted direct effect supported H1 has been removed. (Lines 352–353 and 375–377)

Q3. Lines 257–263 explain that the ERQ comprises the cognitive reappraisal and expressive suppression dimensions, while only the CR subscale was included in the model. Lines 266–267 subsequently report Cronbach’s (Cronbach’s α=0.828) for the ERQ and (Cronbach’s α=0.894) for CR. It is unclear which items were included in the calculation of the first coefficient and why the reliability of the complete ERQ is relevant when only CR was analyzed.

Please clarify at Lines 266–267 which items were used to calculate each reliability coefficient. Because only the CR subscale was included in the reported analyses, its reliability estimate should be emphasized. The reliability estimate for the complete ERQ should be removed if it is not relevant to the present analysis.

Response: Thank you for this comment. We have removed the reliability estimate for the full ERQ (α = 0.828), as only the CR subscale was analyzed. The revised text clarifies that α = 0.894 was calculated using the six CR items (items 1, 3, 5, 7, 8, and 10). (Lines 270–272).

Q4. At Line 5, the institutional affiliation is not capitalized consistently. Please conduct a final systematic language and statistical-formatting check across the main text, tables, figure captions, and notes.

Response: Thank you for this careful observation. We have corrected and standardized the capitalization of the institutional affiliation. We also conducted a final systematic check of language and statistical formatting throughout the manuscript, including the main text, tables, figure captions, and notes.

Attachment Submitted filename: response_letter_for_review_comments_auresp_3.docx

# Peer review: Decision Letter 3

**DOI:** 10.1371/journal.pone.0361222.r007

**Authors:** Rogis Baker

7 Oct 2026

<p>The Impact of Perceived Social Support on College Athletes’ Academic Burnout: The Moderating Role of Cognitive Reappraisal within a Serial Mediation Model

PONE-D-26-07482R3

Dear Xu,

We’re pleased to inform you that your manuscript has been judged scientifically suitable for publication and will be formally accepted for publication once it meets all outstanding technical requirements.

Within one week, you’ll receive an e-mail detailing the required amendments. When these have been addressed, you’ll receive a formal acceptance letter and your manuscript will be scheduled for publication.

An invoice will be generated when your article is formally accepted. Please note, if your institution has a publishing partnership with PLOS and your article meets the relevant criteria, all or part of your publication costs will be covered. Please make sure your user information is up-to-date by logging into Editorial Manager at Editorial Manager® and clicking the ‘Update My Information' link at the top of the page. For questions related to billing, please contact billing support.

If your institution or institutions have a press office, please notify them about your upcoming paper to help maximize its impact. If they’ll be preparing press materials, please inform our press team as soon as possible -- no later than 48 hours after receiving the formal acceptance. Your manuscript will remain under strict press embargo until 2 pm Eastern Time on the date of publication. For more information, please contact onepress@plos.org.

Kind regards,

Rogis Baker, Ph.D

Academic Editor

PLOS One

Additional Editor Comments (optional):

Reviewers' comments:

Reviewer's Responses to Questions

**Comments to the Author**

Reviewer #2: All comments have been addressed

**********

2. Is the manuscript technically sound, and do the data support the conclusions?

Reviewer #2: Yes

**********

3. Has the statistical analysis been performed appropriately and rigorously?

Reviewer #2: Yes

**********

4. Have the authors made all data underlying the findings in their manuscript fully available?

The PLOS Data policy

Reviewer #2: Yes

**********

5. Is the manuscript presented in an intelligible fashion and written in standard English?

Reviewer #2: Yes

**********

Reviewer #2: (No Response)

**********

what does this mean?). If published, this will include your full peer review and any attached files.

If you choose “no”, your identity will remain anonymous but your review may still be made public.

**Do you want your identity to be public for this peer review?**  For information about this choice, including consent withdrawal, please see our Privacy Policy

Reviewer #2: No

**********

# Editor report: Acceptance letter

**DOI:** 10.1371/journal.pone.0361222.r008

**Authors:** Rogis Baker

PONE-D-26-07482R3

PLOS One

Dear Dr. Xu,

I'm pleased to inform you that your manuscript has been deemed suitable for publication in PLOS One. Congratulations! Your manuscript is now being handed over to our production team.

At this stage, our production department will prepare your paper for publication. This includes ensuring the following:

* All references, tables, and figures are properly cited

* All relevant supporting information is included in the manuscript submission,

* There are no issues that prevent the paper from being properly typeset

You will receive further instructions from the production team, including instructions on how to review your proof when it is ready. Please keep in mind that we are working through a large volume of accepted articles, so please give us a few days to review your paper and let you know the next and final steps.

Lastly, if your institution or institutions have a press office, please let them know about your upcoming paper now to help maximize its impact. If they'll be preparing press materials, please inform our press team within the next 48 hours. Your manuscript will remain under strict press embargo until 2 pm Eastern Time on the date of publication. For more information, please contact onepress@plos.org.

You will receive an invoice from PLOS for your publication fee after your manuscript has reached the completed accept phase. If you receive an email requesting payment before acceptance or for any other service, this may be a phishing scheme. Learn how to identify phishing emails and protect your accounts at https://explore.plos.org/phishing.

If we can help with anything else, please email us at customercare@plos.org.

Thank you for submitting your work to PLOS One and supporting open access.

Kind regards,

PLOS One Editorial Office Staff

on behalf of

Dr. Rogis Baker

Academic Editor

PLOS One
